# The genetic landscape of antibiotic sensitivity in *Staphylococcus aureus*

**DOI:** 10.1126/sciadv.aeb9875

**Published:** 2026-05-08

**Authors:** Wan Li, Menghan Liu, Panos Oikonomou, Sydney Blattman, Mirela Berisa, Falguni Paul, Julia Hettleman, Joseph Gonzalez, Qiaoyu Hu, Howe Chen, Saeed Tavazoie, Wenyan Jiang

**Affiliations:** ^1^ Department of Genetics and Genomic Sciences, Icahn School of Medicine at Mount Sinai, New York, NY 10029, USA.; ^2^ Department of Biological Sciences, Columbia University, New York, NY 10027, USA.; ^3^ Metabolomics Core, Icahn School of Medicine at Mount Sinai, New York, NY 10029, USA.; ^4^ Department of Systems Biology, Columbia University, New York, NY 10032, USA.; ^5^ Department of Biochemistry and Molecular Biophysics, Columbia University, New York, NY 10032, USA.; ^6^ Department of Microbiology, Icahn School of Medicine at Mount Sinai, New York, NY 10029, USA.

## Abstract

A comprehensive genetic landscape of antibiotic sensitivity in *Staphylococcus aureus* is lacking. Using genome-scale CRISPR-interference libraries, we systematically quantified global gene fitness across 10 antibiotics and uncovered hundreds of significant antibiotic-gene interactions. Essential genes dominated these interactions, a finding not revealed by transposon-based studies. CRISPR interference repression of transcriptional and translational processes desensitized bacteria to multiple antibiotics. In contrast, repression of cell wall synthesis/cell division (CC), DNA replication/DNA recombination (DD), coenzyme A biosynthesis, and riboflavin metabolism strongly sensitized bacteria to antibiotics. Network and genetic analyses further revealed synergistic genetic interactions (GIs) within these bioprocesses, including an extensive CC-DD subnetwork. Only a subset of CC-DD synergies was dependent on the cell division inhibitor SosA. Informed by these GIs, we identified multiple drug-drug combinations with potent synergistic activity against multidrug-resistant *S. aureus*. Our detailed profiling of drug-gene, gene-gene, and drug-drug interactions reveals important functional relationships among essential genes and defines a vulnerability landscape to guide drug target discovery and effective combination therapies.

## Teaser

CRISPRi profiling defines genome-scale vulnerability landscapes under antibiotics in *S. aureus*.

## INTRODUCTION

The rise of antimicrobial resistance (AMR) is one of the most pressing threats to global public health. In 2021, bacterial AMR was associated with 4.71 million deaths worldwide (*1*). Without effective intervention, AMR is projected to surpass cancer as the leading cause of death by 2050, with an estimated annual economic burden of US $3.5 billion (*2*). Among bacterial pathogens, *Staphylococcus aureus* stands out for its remarkable ability to withstand antibiotics and evade the human immune system, making it a leading cause of infection-related morbidity and mortality (*1*). *S. aureus* has many intrinsic factors that limit antibiotic efficacy. Previous studies using transposon insertion sequencing (Tn-seq) have allowed researchers to survey the contribution of every nonessential gene in the *S. aureus* core genome to bacterial fitness under antibiotic stress (*3*, *4*). These studies have shown that perturbation of diverse biological processes, including cell wall and membrane function (e.g., *mprF* and *tagO*), electron transport chain (ETC) (e.g., *qoxAB*), and two-component systems (e.g., *graRS* and *vraFG*) can strongly modulate antimicrobial sensitivity.

Traditional transposon insertion methodology exerts strong, irreversible perturbations, limiting their utility for studying phenotypes involving essential genes. Consequently, systems-level investigations of how essential genes influence antibiotic sensitivity in *S. aureus* have been lacking. Yet, this knowledge is critical both for advancing our understanding of microbial genetic architecture and for guiding antimicrobial strategies. Systems biology studies have shown that essential genes occupy central positions in genetic interaction (GI) networks in yeast (*5*) and exert markedly stronger effects on antibiotic sensitivity than nonessential genes in *Mycobacterium tuberculosis* (*6*). Insights from these studies could inform the development of effective combinatorial drug therapies that target multiple essential bioprocesses. These therapies offer multiple advantages, including synergistic efficacy, resistance repression, and reduced toxicity within human hosts (*7*–*12*). Combinatorial regimens have proven successful across many medical fields, including infectious diseases (*7*–*9*), HIV (*10*), cancer (*11*), and cardiovascular disease (*12*).

To investigate the effects of essential genes on bacterial phenotypes, we and other groups previously developed the CRISPR interference (CRISPRi) technology (*13*, *14*), which mediates mild transcriptional repression rather than strong gene disruption. Recent applications of CRISPRi in bacteria by us and others have already revealed previously unidentified roles essential genes play in antibiotic sensitivity (*6*, *15*–*18*), showing that the genetic landscapes profiled by Tn-seq are incomplete. For instance, repression of the essential two-component system MtrAB sensitizes *M. tuberculosis* to multiple antibiotics, including rifampicin, vancomycin, and linezolid (*6*). Using single- and dual-CRISPRi libraries in *S. aureus*, we showed that repression of the essential mevalonate and fatty acid synthesis pathways enhances and diminishes, respectively, intrinsic resistance to gentamicin (*15*).

Building upon our previous work on gentamicin (*15*), we applied our systems genomics approach to map the antibiotic sensitivity landscape of *S. aureus* across 10 antibiotics that target cell wall synthesis/membrane, DNA synthesis/replication, and protein translation. Leveraging CRISPR Adaptation-mediated Library Manufacturing (CALM), a CRISPRi functional genomics platform that we developed previously (*15*), we generated highly comprehensive genome-scale libraries in both methicillin-susceptible *S. aureus* (MSSA) and methicillin-resistant *S. aureus* (MRSA). Our screens revealed hundreds of significant antibiotic-gene interactions, with essential genes dominating these interactions. Repression of essential genes could either sensitize or desensitize *S. aureus* to antibiotics, a finding not revealed by previous transposon-based studies (*3*, *4*). Among the most vulnerable biological processes were cell wall synthesis/cell division (CC), DNA replication/DNA recombination (DD), coenzyme A biosynthesis, and riboflavin metabolism, as their repression significantly reduced bacterial fitness in antibiotics of various modes of action. The scale of our antibiotic-gene interaction dataset enabled the construction of an essential gene similarity network, which uncovered extensive synergistic GIs among essential genes within these processes. Last, we translated several previously unidentified synergistic GIs into antimicrobial combinations that exhibited potent killing against both MSSA and multidrug-resistant MRSA. Together, our comprehensive profiling of drug-gene, gene-gene, and drug-drug interactions illuminates critical functional relationships within the *S. aureus* core essential genome and provides a rational foundation for developing effective combinatorial antimicrobial therapies. Our work also places *S. aureus* among the few pathogens for which genome-scale CRISPRi-based characterization of antibiotic sensitivity has been achieved, alongside *M. tuberculosis* (*6*).

## RESULTS

**Fig. 1.**
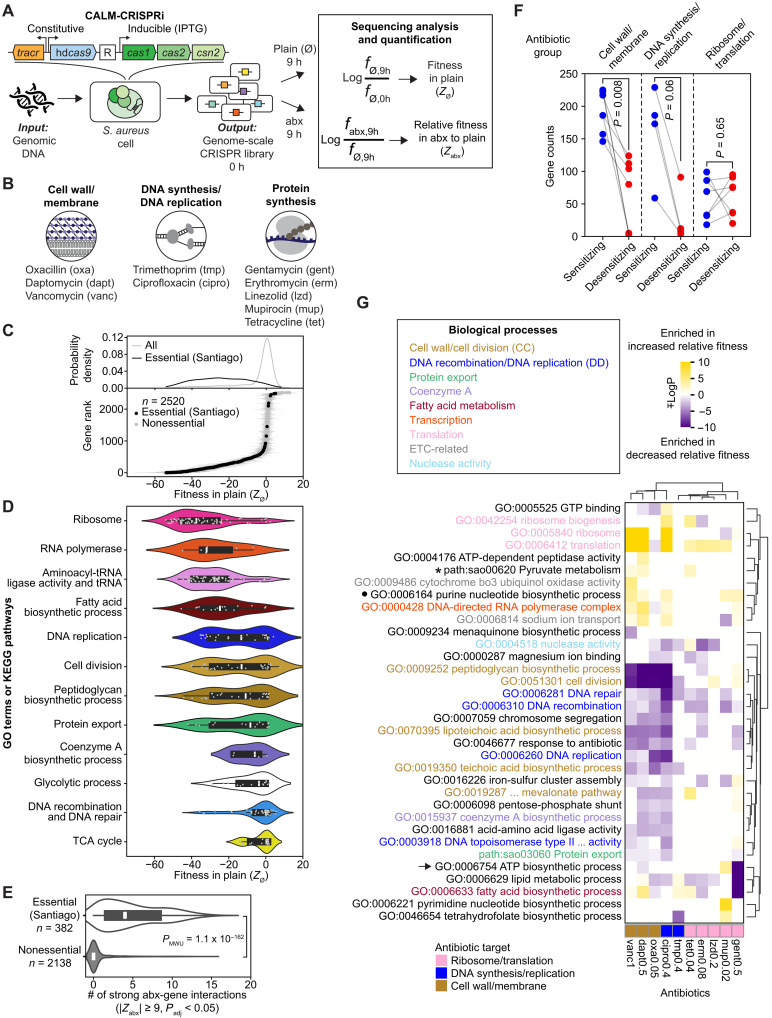
CALM-CRISPRi reveals genetic landscapes of essentiality and antibiotic sensitivity in *Staphylococcus aureus*. (**A**) CALM-CRISPRi enables genome-scale screening in *S. aureus*. *Tracr*, hd*Cas9* (hyper-dCas9), and R (crRNA) are constitutively expressed to mediate transcriptional repression. CRISPRi libraries were grown in plain TSB (Ø) or sublethal concentrations of 10 antibiotics (abx), followed by sequencing and quantification. (**B**) The 10 antibiotics used in this study. (**C**) Distribution of gene fitness in plain TSB (*Z*_Ø_). Bottom: *S. aureus* RN4220 genes ranked by mean fitness in TSB (*Z*_Ø_), quantified by CRISPRi from three biological replicates; error bars indicates SD. Of 2672 annotated genes, only genes targeted by at least two crRNAs were included (*n* = 2520) in analysis. Essential and nonessential genes, defined by the Tn-seq study of Santiago *et al.* (*22*), are shown as black and gray dots, respectively. Top: Distributions of essential genes (black) and all genes (gray). *Z*_Ø_ values are provided in table S2. (**D**) Distributions of gene fitness (mean *Z*_Ø_ from triplicates) for core essential biological processes. (**E**) Antibiotic-gene interactions by gene essentiality. Violin plots show the number of antibiotic conditions in which repression of essential and nonessential genes significantly altered relative fitness (|*Z*_abx_| ≥ 9, *P*_adj_ < 0.05). A Mann-Whitney *U* test was performed. (**F**) Numbers of significant sensitizing (blue) and desensitizing (red) antibiotic-gene interactions (|*Z*_abx_| ≥ 9, *P*_adj_ < 0.05) across antibiotic groups. Eighteen antibiotic conditions shown in fig. S3A were included in analysis. *P* values from Wilcoxon tests are shown. (**G**) Functional enrichment analysis, followed by clustering of significantly enriched nonredundant GO terms and KEGG pathways. ∓log(*P* values) for pathways significantly enriched (*P* < 0.001) among genes whose repression increased and decreased relative fitness in antibiotics (|*Z*_abx_| ≥ 9, *P*_adj_ < 0.05) are shown in yellow and purple, respectively. Full clustering results are shown in fig. S4 (A and C) and tables S6 and S8.

Harnessing the natural capacity of CRISPR adaptation—a bacterial immunization process that stores nucleic acid sequence information from invaders such as phages—the CALM methodology that we developed (*15*) can rapidly generate comprehensive genome-scale CRISPR RNA (crRNA) libraries that cover 95% of all targetable sites in the *S. aureus* genome, bypassing the conventional, resource-intensive steps of guide RNA (gRNA) synthesis and cloning. As outlined in Fig. 1A, *S. aureus* cells harboring an engineered CRISPR adaptation machinery convert genomic DNA into ultradense genome-scale crRNA libraries. The canonical crRNA, along with another small RNA encoded by *tracr*, is functionally equivalent to the engineered gRNA (*19*). Both crRNA and dCas9 are constitutively expressed. To profile the genetic landscape of antibiotic sensitivity, we cultured libraries generated in strains RN4220 (*20*) (MSSA) and JE2 (*21*) (a USA300 lineage of MRSA) in plain tryptic soy broth (TSB) media or TSB containing antibiotics targeting cell wall synthesis/membrane, DNA synthesis/replication, and protein translation (Fig. 1B). The 10 antibiotics were supplied at sublethal concentrations (Materials and Methods and fig. S1A), and bacterial cultures were grown for 9 hours, reaching early stationary phase.

The CRISPRi libraries generated by CALM were highly diverse, with nearly 90% of genes covered by three or more coding-strand–targeting (CT) crRNAs and a median of ≥10 CT crRNAs per gene (fig. S1B), conferring statistical robustness. We used CT crRNAs to quantify gene fitness, as they mediate strong transcriptional repression (*13*, *14*). For each crRNA, we quantified its fitness in plain media ($\text{log}\frac{f_{ø,9h}}{f_{ø,0h}}$) and the relative fitness in antibiotic compared to plain media ($\text{log}\frac{f_{\text{abx},9h}}{f_{ø,9h}}$) (Fig. 1A). Next, a gene score was calculated by averaging the values from all CT crRNAs targeting that gene. Last, we standardized the gene score by using a pool of null, intergenic crRNAs, yielding a *Z*-score (*Z*_Ø_ and *Z*_abx_) as the final fitness score (Fig. 1A and Materials and Methods). This standardization allows direct comparison of gene fitness effects across different samples and antibiotic treatments—for example, a *Z*-score of ±1 indicates that the fitness effect of a gene’s repression is 1 SD from the mean of the null distribution. Since *Z*_abx_ reflects the relative fitness in antibiotics compared to plain media, negative and positive *Z*_abx_ values indicate sensitization and desensitization of bacteria to antibiotics, respectively. *Z*-scores from independent biological replicates showed strong Pearson correlations: 0.89 < *r* < 0.97 in plain media (fig. S1, D and E) and 0.77 < *r* < 0.93 in antibiotic conditions (fig. S1F).

Our CALM-CRISPR libraries showed good performance in identifying essential genes: 0.91 < area under the curve (AUC) < 0.95 (fig. S1, G and H); genes quantified with low fitness (*Z*_Ø_) in plain media substantially overlapped with essential genes identified in the Tn-seq study of Santiago *et al.* (*22*) (Fig. 1C). The degree of essentiality quantified by CRISPRi varied widely across core essential biological processes, ranging from highly essential genes encoding the ribosome, RNA polymerase, tRNA ligase/tRNA, and fatty acid biosynthesis pathway, to less essential genes involved in coenzyme A biosynthesis, glycolysis, DNA recombination/DNA repair, and TCA cycle (Fig. 1D and fig. S2). A similar pathway-level differential essentiality profile was also found in *Mycobacterium* (*23*), suggesting that gene vulnerability may be at least partially conserved across microbial species.

### A genetic landscape of antibiotic sensitivity dominated by essential genes

Six hundred fifty of ~2600 annotated genes in the RN4220 genome showed significantly altered relative fitness (|*Z*_abx_| ≥ 9 and *P*_adj_ < 0.05) in at least one antibiotic condition compared to plain media, among a total of 10 antibiotics that we tested (fig. S3A). Hierarchical clustering of these genes formed two major groups. The lower group consisted of antibiotics that target cell wall/membrane and DNA replication, which are vancomycin, daptomycin, oxacillin, and ciprofloxacin. The upper group included mostly antibiotics targeting protein synthesis, with trimethoprim as an exception. Notably, we found that essential genes dominated the antibiotic-gene interactions. Perturbation of 66% of essential genes identified by by the Tn-seq studies of Santiago *et al.* (*22*) and Bae *et al.* (*24*) significantly altered relative fitness in antibiotics (black bars in fig. S3A). Overall, repression of essential genes led to strong changes in relative fitness (|*Z*_abx_| ≥ 9 and *P*_adj_ < 0.05) in a median of four antibiotic conditions, while repression of ~80% of the 2138 nonessential genes did not significantly alter relative fitness in any antibiotic condition (Fig. 1E). These results indicate that CRISPRi, with its ability to mildly perturb essential genes, captures antibiotic-gene interactions that are not accessible using strong loss-of-function Tn-seq approaches. Only 1.7 to 7.1% of nonessential genes significantly modulated relative fitness in antibiotic conditions (fig. S3B), and they were enriched in processes such as adenosine triphosphate (ATP) biosynthesis and ETC (fig. S4D). In addition, for antibiotics that target cell wall/membrane and DNA synthesis/replication, sensitizing interactions (i.e., genes whose repression decreased relative fitness in the presence of antibiotics) consistently outnumbered desensitizing interactions (i.e., genes whose repression increased relative fitness), with cell wall/membrane–targeting antibiotics reaching statistical significance (*P*_Wilcoxon_ = 0.008) (Fig. 1F and fig. S3C). In contrast, translation-targeting antibiotics did not show this trend.

To gain insights into the biological functions of genes that modulate bacterial fitness in the antibiotics, we performed functional enrichment analysis and clustering. This analysis identified significantly overrepresented Gene Ontology (GO) terms (Fig. 1G and fig. S4A) and Kyoto Encyclopedia of Genes and Genomes (KEGG) pathways (fig. S4C) among genes whose repression decreased (purple) or increased relative fitness (yellow) in antibiotics. As expected, inhibition of many biological processes sensitized bacteria to antibiotics with related modes of action. For instance, CC processes (brown labels in Fig. 1G) were highly enriched among genes whose repression reduced relative fitness in cell wall/membrane–targeting antibiotics such as oxacillin, daptomycin, and vancomycin. Likewise, DD processes (blue labels in Fig. 1G) were enriched among genes whose repression reduced relative fitness in ciprofloxacin, which target DNA replication. Because bacterial genes are organized in operons, functional enrichment can be confounded as a result of silencing nearby cotranscribed genes involved in different biological processes. To address this, we also performed a more conservative enrichment analysis using only the last gene in each operon (table S1). Although removing ~1000 genes reduced the sensitivity of the analysis, this last-gene-in-operon approach still produced consistent enrichment profiles, with antibiotic conditions showing an average Pearson correlation of 0.80 (fig. S4B) compared to profiles generated using all genes.

### Repression of CC and DD processes sensitizes bacteria to many antibiotics

**Fig. 2.**
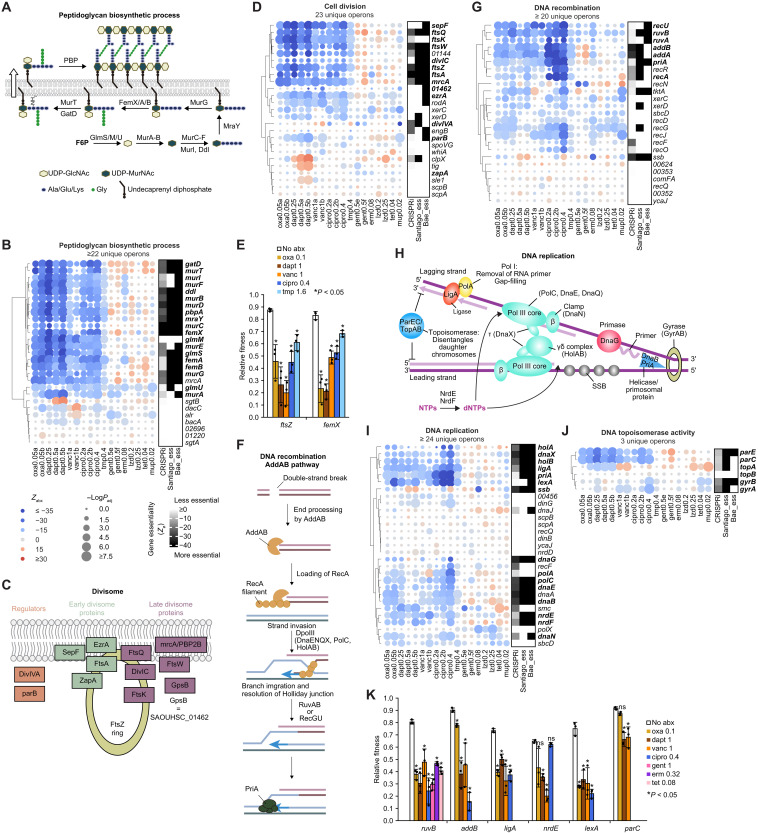
Repression of CC and DD processes sensitizes bacteria to many antibiotics. (**A**) Peptidoglycan biosynthetic pathway. (**B**) Left: Blue-red heatmap showing the relative fitness of genes encoding the peptidoglycan biosynthetic process (GO:0009252) across 10 antibiotics in *S. aureus* RN4220, quantified by CALM-CRISPRi. Antibiotic conditions with replicates are labeled with letters. Gene names are annotated by KEGG orthology (ko). *glmS* and *pbpA* were also included because of overannotation by GO terms. Genes that appear in (A) are bolded. Gene knockdowns that decreased or increased relative fitness in antibiotics are shown as blue and red circles, respectively. Circle size indicates −log(*P*_adj_), calculated using a Mann-Whitney *U* test comparing gene-specific crRNAs and null crRNAs (Materials and Methods). Right: Black-white heatmap showing gene essentiality quantified by CRISPRi (mean *Z*_Ø_ from triplicates) and qualified (binary) by the Tn-seq studies of Santiago *et al.* (*22*) and Bae *et al.* (*24*). (**C**) The divisome. (**D**) Same as (B) but showing genes encoding cell division (GO:0051301). *ftsK*, *mrcA*, and *zapA* were also included because of overannotation by GO terms. *Mur* genes already shown in (B) were omitted. (**E**) Pairwise competition assays (Materials and Methods) measuring relative fitness of *S. aureus* RN4220 carrying an IPTG-inducible CRISPRi targeting *ftsZ* or *femX* under indicated antibiotic conditions. Antibiotic conditions are shown in micrograms per milliliter. Data represent the means ± SD from three biological replicates. For each gene knockdown, paired *t* tests were performed between antibiotic and no-antibiotic conditions. (**F**) The AddAB (Gram-positive homolog of RecBCD) DNA recombination pathway. (**G**) Same as (B) but showing genes encoding DNA recombination (GO:0006310). *addA*, *addB*, and *recF* were also included because of overannotation by GO terms. (**H**) DNA replication. (**I** and **J**) Same as (B) but showing genes encoding DNA replication (GO:0006260) and DNA topoisomerase activity (GO:0003916), respectively. (**K**) Same as (E) but showing relative fitness of other genes.

Our CALM screens revealed that CC processes were among the most vulnerable, as their knockdown sensitized bacteria to many antibiotics that we tested (Fig. 1G and fig. S4A). These figures also show that in addition to the antibiotics that target cell wall/membrane, many CC processes were also significantly enriched among genes whose repression reduced relative fitness in antibiotics that target DNA synthesis/replication (i.e., trimethoprim and ciprofloxacin). At the gene level, we found that inhibition of a substantial portion of essential genes in peptidoglycan biosynthesis (GO:0009252) and cell division (GO:0051301) significantly decreased relative fitness in ciprofloxacin and trimethoprim (Fig. 2, A to D), a phenotype not captured by a previous Tn-study (*3*). To validate these CRISPRi screening results, we cloned CT crRNAs targeting representative essential gene hits, *ftsZ* and *femX*, with an isopropyl-β-D-thiogalactopyranoside (IPTG)-inducible dCas9 and quantified their fitness relative to that of a nontargeting control in antibiotics (“Pairwise competition assay” section in Materials and Methods). Whenever possible, we chose to validate genes that are the last in operons (fig. S5A) to avoid polar effects of CRISPRi. We used IPTG concentrations that moderately reduced the fitness of candidate cells to 50 to 90% of that of the nontargeting control under no antibiotic treatment, as excessive inhibition of essential genes led to strong growth defect (or death) even without antibiotics (fig. S5A). Our approach confirmed that mild inhibition of *ftsZ* and *femX* significantly reduced relative fitness in oxacillin, daptomycin, vancomycin, ciprofloxacin, and trimethoprim (Fig. 2E). Similarly, inhibition of other essential processes upstream of peptidoglycan synthesis, such as the mevalonate pathway (*25*) (fig. S4E) that synthesizes the precursor to undecaprenyl phosphate, and pathways encoding cell surface glycopolymers specific to Gram-positive bacteria, including teichoic acid and lipoteichoic acid biosynthetic processes (*26*), also sensitized bacteria to these five antibiotics (fig. S4, F and G).

DD processes were also highly vulnerable, as their repression sensitized bacteria to many antibiotics (Fig. 1G and fig. S4A). As expected, knockdown of genes encoding the two bacterial DNA homologous recombination (GO:0006310) pathways, RecFOR and AddAB [the RecBCD homolog in *S. aureus* (*27*); Fig. 2F], led to the greatest reduction in relative fitness in ciprofloxacin (bolded genes in Fig. 2G). Relative fitness in other antibiotics, especially those that target cell wall/membrane, also significantly decreased. For example, *Z*_abx_ values of *addAB*, encoding the Gram-positive RecBCD homolog, and those of *ruvAB*, encoding the essential Holliday junction DNA helicase, were all around −15 in oxacillin, daptomycin, and vancomycin (Fig. 2G). Repression of additional essential genes in DD (Fig. 2, H to J)—including those encoding the DNA replication machinery (e.g., *ligA*, *polC*, *dnaEX*, *holAB*, *priA*, and *dnaB*), the ribonucleoside-diphosphate reductase (*nrdEF*) that converts nucleotide triphosphates to deoxynucleotide triphosphates, the DNA repair regulator *lexA*, and DNA topoisomerase IV (*parEC*)—also reduced relative fitness in all or a subset of the three cell wall/membrane–targeting antibiotics. Using pairwise competition, we validated the reduced relative fitness of *ruvB*, *addB*, *ligA*, *nrdE*, *lexA*, and *parC* in most antibiotic conditions (Fig. 2K), with three conditions in *nrdE* and *parC* not reaching statistical significance. Notably, *ruvB* knockdown also sensitized bacteria to translation inhibitors including gentamicin, erythromycin, and tetracycline (Fig. 2, G and K). Supporting these results obtained with pairwise competition, repression of these six genes, except for *parC*, showed a clear reduction in their minimum inhibitory concentration (MIC) in oxacillin (fig. S5, B, I, J, O to R). Since *ruvB* and *ligA* precede genes of other functions in their respective operons (fig. S5A), we also conducted complementation experiments to confirm their roles in antibiotic sensitivity (fig. S5, S to V). Together, these findings revealed CC and DD as highly vulnerable bioprocesses where their inhibition sensitizes *S. aureus* to various antibiotics, especially those that target cell wall/membrane and DNA synthesis.

### Other prominent sensitizing antibiotic-gene interactions

**Fig. 3.**
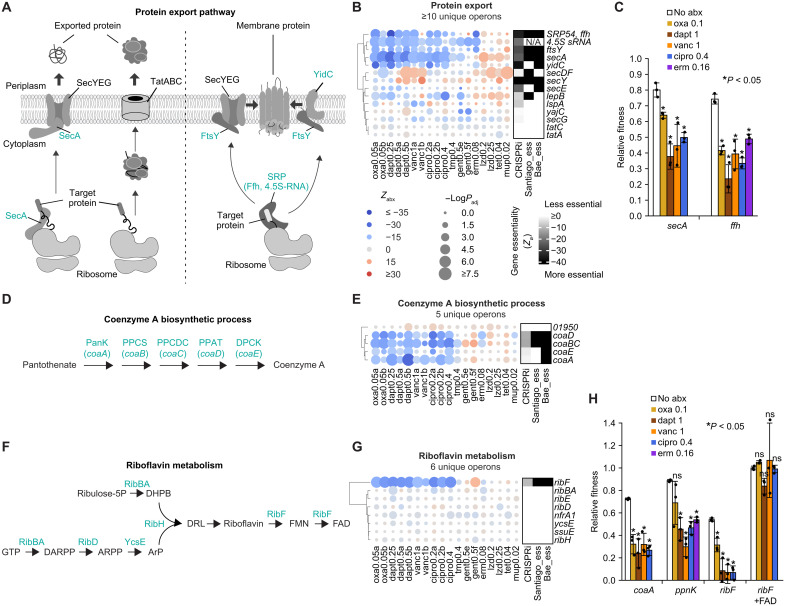
Repression of diverse biological processes sensitizes bacteria to antibiotics. (**A**) Protein export (left) and targeting to the membrane (right). Components involved in strong sensitizing interactions are labeled in green. (**B**) Left: Blue-red heatmap showing the relative fitness of genes (*Z*_abx_) encoding the protein export pathway (KEGG path:sao03060) across 10 antibiotics in *S. aureus* RN4220, quantified by CALM-CRISPRi. Antibiotic conditions with replicates are labeled with letters. Gene names are annotated by KEGG orthology (ko). The 4.5S sRNA was also added to the heatmap. Genes that appear in schematic in (A) are bolded. Gene knockdowns that decreased or increased relative fitness in antibiotics are shown as blue and red circles, respectively. Circle size indicates −log(*P*_adj_), calculated using a Mann-Whitney *U* test comparing gene-specific crRNAs and null crRNAs (Materials and Methods). Right: Black-white heatmap showing gene essentiality quantified by CRISPRi (mean *Z*_Ø_ from triplicates) and qualified (binary) by the Tn-seq studies of Santiago *et al.* (*22*) and Bae *et al.* (*24*). (**C**) Pairwise competition assays (Materials and Methods) measuring the relative fitness of *S. aureus* RN4220 carrying an IPTG-inducible CRISPRi targeting *secA* or *ffh* under indicated antibiotic conditions. Antibiotic conditions are shown in micrograms per milliliter. Data represent the means ± SD from three biological replicates. For each gene knockdown, paired *t* tests were performed between antibiotic and no-antibiotic conditions. (**D**) Coenzyme A biosynthesis process. (**E**) Same as (B) but showing genes encoding the coenzyme A biosynthetic process (GO:0015937). (**F**) Riboflavin metabolism. (**G**) Same as (B) but showing genes encoding the riboflavin metabolism (KEGG path:sao00740). (**H**) Same as (C) but showing the relative fitness of repression of *coaA*, *ppnK*, *ribF*, and *ribF* supplemented with 600 μM FAD.

Our functional enrichment analysis identified additional essential pathways whose inhibition strongly sensitized bacteria to antibiotics that target cell wall/membrane and DNA synthesis. Many of these pathways, including protein export (green label in Fig. 1G) and the biosynthesis of essential organic cofactors such as coenzyme A (purple label in Fig. 1G), flavin adenine dinucleotide (FAD), and nicotinamide adenine dinucleotide (NAD), were not captured by previous transposon studies (*3*, *4*). Protein integration and transport across the membranes is an essential process ubiquitous to all domains of life. In bacteria, transmembrane proteins are inserted into the membrane by the signal recognition particle (SRP) and the Sec translocation machinery and channels (*28*) (Fig. 3A). We found that inhibition of a subset of protein transport components, especially the core of SRP (4.5*S* RNA and Ffh) and the adenosine triphosphatase motor of the Sec translocation machinery (SecA), potentiated oxacillin, daptomycin, vancomycin, and ciprofloxacin (Fig. 3, B and C). Inhibition of biosynthetic processes of a number of essential organic cofactors central to cellular metabolism, such as coenzyme A, FAD, and NAD, also potentiated these four antibiotics (Fig. 3, D to G, and fig. S4H). We confirmed that knockdown of the enzymes responsible for producing these cofactors, including the essential *coaA*, *ribF*, and *ppnK*, indeed reduced relative fitness in these antibiotics (Fig. 3H), as well as the MICs of oxacillin (fig. S5, B, F, L, and M) and ciprofloxacin (fig. S5W). For bacteria with *ribF* knockdown, we also chemically rescued the phenotype by supplementing growth media with FAD (Fig. 3H), a key cofactor synthesized by RibF (Fig. 3F). This indicates that reduced FAD level due to *ribF* repression sensitizes bacteria to various antibiotic stress.

We also identified sensitizing interactions between genes and translation-targeting antibiotics. A notable hit was DNA recombination (Fig. 1G), and we confirmed that *ruvB* knockdown sensitized bacteria to multiple translation inhibitors (Fig. 2K), an interaction that has not been described in prior studies. By contrast, many other sensitizing interactions observed here are consistent with previously reported findings. For instance, strong sensitizing interactions between gentamicin and repression of ATP biosynthesis (arrows in Fig. 1G and fig. S4D), as well as fatty acid biosynthesis (maroon label in Fig. 1G), agree with results reported by us and others (*15*, *29*). We also observed sensitizing interactions involving nuclease activity (light blue label in Fig. 1G). Knockdown of genes encoding ribonucleases—such as *rnjA*, which mediates global mRNA decay (*30*), and *rnr*, which participates in RNA degradation and trans-translation (*31*, *32*), has been previously shown to reduce bacterial tolerance to ribosome-targeting antibiotics in *Escherichia coli* and *Bacillus subtilis*. Here, we demonstrated that knockdown of these genes also strongly sensitized *S. aureus* to multiple translation-targeting antibiotics (bolded genes in fig. S4I), including two- to fourfold reductions in MICs of erythromycin, linezolid, and mupirocin (fig. S5Y). *rnjA* knockdown also lowered the MIC of oxacillin by fourfold (fig. S5N). Collectively, Fig. 3 and fig. S4 (E to I) showcase diverse antibiotic-potentiating processes enriched in essential genes, establishing a genetic basis to guide combinatorial antimicrobial strategies (see section “Drug-drug synergies”).

### Desensitizing antibiotic-gene interactions

**Fig. 4.**
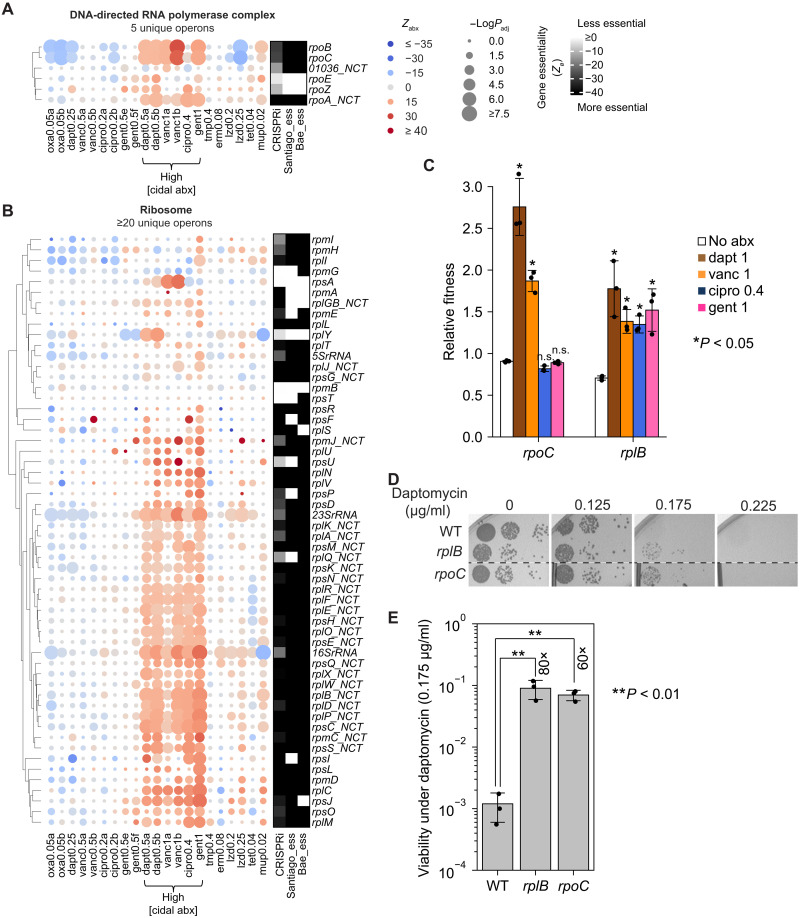
Repression of transcription and translation desensitizes bacteria to bactericidal antibiotics. (**A**) Left: Blue-red heatmap showing the relative fitness of genes (*Z*_abx_) encoding the DNA-directed RNA polymerase complex (GO:0000428) across 10 antibiotics in *S. aureus* RN4220, quantified by CALM-CRISPRi. Antibiotic conditions with replicates are labeled with letters. Unlike other conditions, “gent1” shows *Z*_abx_ of a library treated with gentamicin (1 μg/ml) for 4.5 hours from a previous study (*15*). Gene names are annotated by KEGG orthology (ko). Gene knockdowns that decreased or increased relative fitness in antibiotics are shown as blue and red circles, respectively. Genes labeled “NCT” were quantified using NCT crRNAs because of extreme essentiality (“Quantification using NCT crRNAs” in Materials and Methods). Circle size indicates −log(*P*_adj_), calculated using a Mann-Whitney *U* test comparing gene-specific crRNAs and null crRNAs (Materials and Methods). Right: Black-white heatmap showing gene essentiality quantified by CRISPRi (mean *Z*_Ø_ from triplicates) and qualified (binary) by the Tn-seq studies of Santiago *et al.* (*22*) and Bae *et al.* (*24*). (**B**) Same as (A), showing genes encoding the ribosome (GO:0005840). (**C**) Pairwise competition assays (Materials and Methods) measuring relative fitness of *S. aureus* RN4220 carrying an IPTG-inducible CRISPRi targeting *rpoC* or *rplB* under indicated antibiotic conditions. Antibiotic conditions are shown in micrograms per milliliter. Data represent the means ± SD from three biological replicates. For each gene knockdown, paired *t* tests were performed between antibiotic and no-antibiotic conditions. n.s., not significant. (**D**) Viability of WT RN4220 and strains carrying IPTG-inducible CRISPRi targeting *rplB* or *rpoC* under daptomycin. Ten-fold serial dilutions were spotted on tryptic soy agar (TSA) containing indicated daptomycin concentrations (0 to 0.225 μg/ml) and 0.01 mM IPTG to mildly repress target genes, followed by 18 hours of incubation at 37°C. (**E**) Quantification of bacterial viability under daptomycin (0.175 μg/ml) in (D), calculated as the ratio of CFUs on daptomycin to CFUs without daptomycin. Data represent the means ± SD from three biological replicates.

Repression of essential genes can not only sensitize but also desensitize bacteria to antibiotics. We found that genes encoding the transcription (e.g., GO:0000428) and translation machinery (e.g., GO:0006412) were prevalent among desensitizing interactions (orange and pink labels in Fig. 1G), especially in high concentrations of bactericidal antibiotics (dapt 0.5, vanc 1, cipro 0.4, and gent 1 in Fig. 4, A and B). Notably, even mild repression of many ribosomal genes by noncoding-strand–targeting (NCT) crRNAs significantly increased relative fitness in these antibiotics (genes labeled with NCT in Fig. 4B), complementing the incomplete data generated from strong CT crRNAs (fig. S6A and see the “Quantification using NCT crRNAs” section in Materials and Methods). Pairwise competition assays confirmed that inhibition of *rpoC* and *rplB*, encoding subunits of RNA polymerase and the ribosome, respectively, significantly increased bacterial fitness in daptomycin and vancomycin (Fig. 4C), with *rplB* inhibition also desensitizing bacteria to ciprofloxacin and gentamicin. Agar-based viability assays further showed that while cells with mild repression of *rplB* or *rpoC* were still killed by bactericidal antibiotics such as daptomycin, their survival was increased by ~80- and 60-fold, respectively, compared with the wild-type (WT) strain (Fig. 4, D and E). These results establish a genetic basis for prior findings that pharmacological inhibition of transcription or translation significantly reduces antibiotic lethality (*33*, *34*), potentially by slowing metabolism (*33*) and/or inducing bacterial persistence (*34*–*37*).

**Fig. 5.**
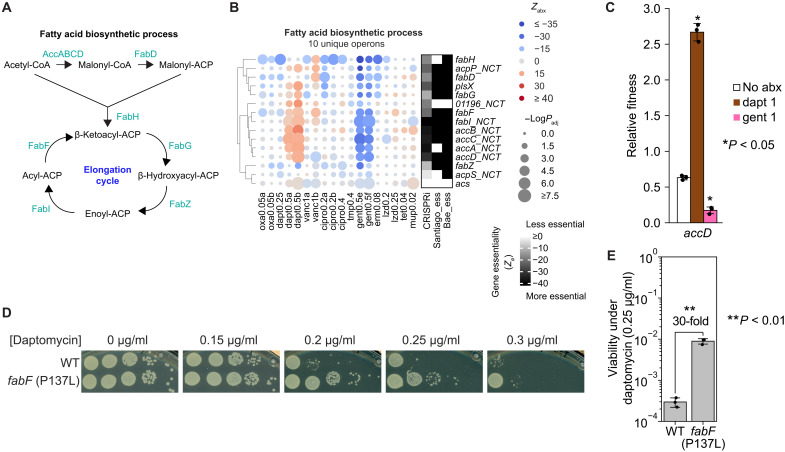
Fatty acid biosynthesis is associated with daptomycin sensitivity. (**A**) Fatty acid biosynthetic process in bacteria. (**B**) Left: Blue-red heatmap showing the relative fitness of genes (*Z*_abx_) encoding the fatty acid biosynthetic process (GO:0006633) across 10 antibiotics in *S. aureus* RN4220, quantified by CALM-CRISPRi. Antibiotic conditions with replicates are labeled with letters. Gene names are annotated by KEGG orthology (ko). Gene knockdowns that decreased or increased relative fitness in antibiotics are shown as blue and red circles, respectively. Genes labeled “NCT” were quantified using NCT crRNAs because of extreme essentiality (“Quantification using NCT crRNAs” in Materials and Methods). Circle size indicates −log(*P*_adj_), calculated using a Mann-Whitney *U* test comparing gene-specific crRNAs and null crRNAs (Materials and Methods). Right: Black-white heatmap showing gene essentiality quantified by CRISPRi (mean *Z*_Ø_ from triplicates) and qualified (binary) by the Tn-seq studies of Santiago *et al.* (*22*) and Bae *et al.* (*24*). (**C**) Pairwise competition assays (Materials and Methods) measuring the relative fitness of *S. aureus* RN4220 carrying an IPTG-inducible CRISPRi targeting *accD* under indicated antibiotic conditions. Antibiotic conditions are shown in micrograms per milliliter. Data represent the means ± SD from three biological replicates. For each gene knockdown, paired *t* tests were performed between antibiotic and no-antibiotic conditions. * indicates *P* < 0.05. (**D**) Viability of WT *S. aureus* RN4220 and an isogenic strain carrying a *fabF* (P137L) mutation under daptomycin. Ten-fold serial dilutions were spotted on TSA containing indicated daptomycin concentrations (0 to 0.3 μg/ml), followed by 24 hours of incubation at 37°C. (**E**) Quantification of bacterial viability under daptomycin (0.25 μg/ml) in (D), calculated as the ratio of CFUs on daptomycin (0.25 μg/ml) to CFUs without daptomycin. Data represent the means ± SD from three biological replicates.

While repression of fatty acid biosynthesis (Fig. 5A) sensitized bacteria to gentamicin, it unexpectedly desensitized bacteria to daptomycin (Fig. 5B), an effect that we validated using the pairwise competition assay (Fig. 5C). Clinical mutations in fatty acid biosynthesis associated with daptomycin resistance or tolerance have been reported infrequently. One study identified a nonsynonymous *fabF* mutation (P137L) in a clinical *S. aureus* isolate following prolonged daptomycin treatment (*38*). However, this isolate also carried an *mprF* mutation (T345I), which is known to confer moderate levels of daptomycin tolerance (*39*), as well as multiple additional genomic mutations, making the contribution of *fabF* (P137L) difficult to assess. To directly evaluate its effect, we introduced the *fabF* (P137L) mutation into RN4220 and found that it significantly increased bacterial survival under sublethal daptomycin by 30-fold (Fig. 5, D and E).

Repression of other essential bioprocesses, including pyruvate metabolism, electron transport, and purine nucleotide biosynthesis, also desensitized bacteria to multiple bactericidal antibiotics (asterisk symbol, gray labels, and dot symbol in Fig. 1G and fig. S6B). We reasoned that antibiotic-desensitizing mutations may be prevalent in laboratory-evolved mutants and clinical isolates as a consequence of repeated antibiotic exposure. Consistent with this idea, mutations disrupting multiple genes in these pathways—including *pyk* (*40*), *pdh* (*40*), *hem* (*41*), *mnh* (*42*), *prs* (*40*, *43*), and *gua* (*42*)—have been reported following laboratory evolution under antibiotic selection (fig. S6B). Next, to assess the prevalence of such mutations in clinical settings, we analyzed 4284 complete *S. aureus* genomes from the National Center for Biotechnology Information (NCBI) Pathogen Detection database and identified antibiotic-resistance mutations affecting multiple genes (fig. S6C). These included truncations of HemY (also known as HemG) involved in heme biosynthesis (*44*, *45*), truncations of proteins comprising Complex I of the ETC (*46*) (e.g., MnhA, MnhC, MnhD, and MnhE), and a nonsynonymous mutation compromising the function of *prs* involved in the purine nucleotide synthesis pathway (*47*). Mutations affecting *pur* genes are also abundant in *E. coli* clinical isolates (*48*) (fig. S6B). Together, our functional genomics screen identifies multiple essential biological pathways that bacteria exploit to acquire antibiotic tolerance/resistance, supported by strong evidence of function-impairing mutations observed in laboratory evolution experiments and clinical isolates.

### Antibiotic-gene interactions in MRSA JE2

We also performed CALM-CRISPRi screens in the MRSA strain JE2 under daptomycin and vancomycin, two frontline antibiotics used to treat MRSA infections. Functional enrichment analysis revealed largely similar antibiotic-pathway interaction landscapes between JE2 and RN4220, with Pearson correlation exceeding 0.85 (fig. S7A). For instance, biological processes such as DNA replication and DNA recombination were enriched for genes whose repression significantly reduced relative fitness in both daptomycin and vancomycin (fig. S7, A and B), corroborating earlier observations in RN4220. Despite these broad similarities, we identified notable strain-specific differences at the gene and pathway levels. In particular, multiple genes involved in peptidoglycan biosynthesis, teichoic acid biosynthesis, and DNA helicase activity exhibited reduced vulnerability to vancomycin in JE2 compared with RN4220 (fig. S7, A and C to E). These included the AddAB helicase/nuclease complex (bolded in fig. S7E), which plays a central role in DNA repair and recombination. Consistent with the screen results, repression of *addB* significantly sensitized RN4220, but not JE2, to vancomycin (fig. S7F). In contrast, a small subset of genes in DNA replication (bolded in fig. S7G) differentially sensitized JE2 to vancomycin, including *scpA*, which is crucial for chromosome segregation and condensation (*49*) and was validated using pairwise competition assays (fig. S7H). Together, these results demonstrate that while RN4220 and JE2 share similar global antibiotic-pathway interaction profiles, nuanced gene-level differences exist between the two strains. Future work applying functional genomics to a broader set of MSSA and MRSA lineages may further elucidate differential antibiotic sensitivities in *S. aureus* and reveal evolutionary trade-offs between genetic vulnerability and multidrug resistance.

### Gene similarity networks and GIs

**Fig. 6.**
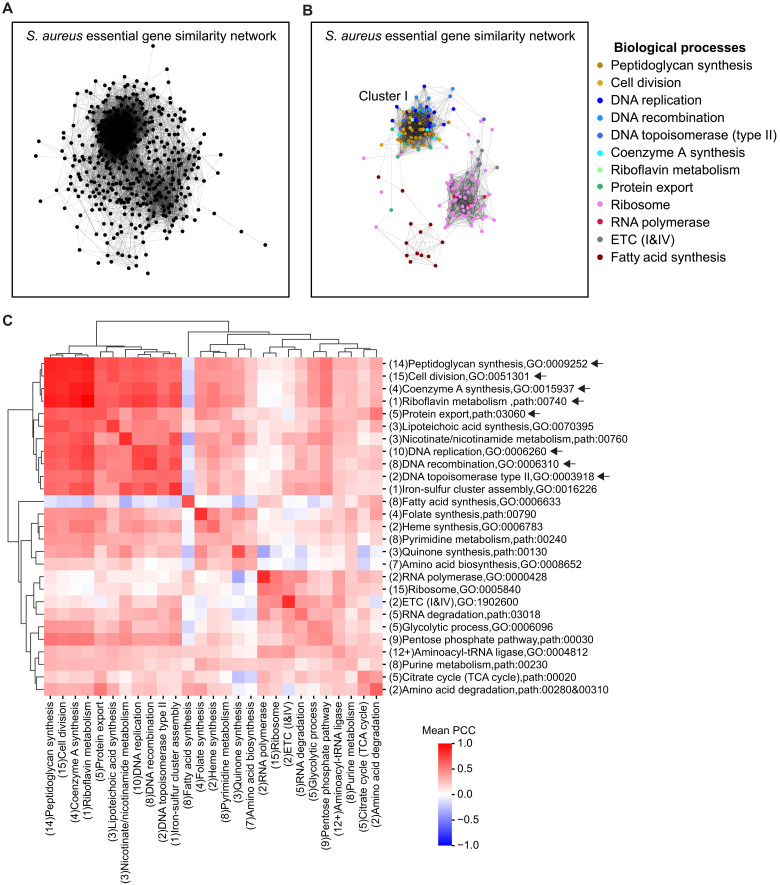
*S. aureus* essential gene similarity network. (**A**) Essential gene similarity network in *S. aureus* RN4220. Pairwise correlations between genes were calculated from the fitness profiles of 440 essential genes, defined as essential in either the Tn-seq studies of Santiago *et al.* (*22*) or Bae *et al.* (*24*), in 14 antibiotic conditions (Materials and Methods). Gene pairs with PCC of greater than 0.65 were connected by gray edges of fixed thickness. The PCC matrix and gene coordinates are provided in tables S12 and S13, respectively. (**B**) Same as (A) but displaying essential genes from selected biological processes and the connections among them (PCC > 0.65). (**C**) Hierarchical clustering of the pairwise mean PCCs among 27 biological processes (Materials and Methods and table S14). Numbers in parentheses indicate the number of unique operons containing essential genes within each process. Arrows indicate processes shown in cluster I in (B).

The genome-scale antibiotic-gene interaction profiles enabled network analysis, uncovering functional relationships among genes and processes. We constructed an *S. aureus* essential gene similarity network based on the interaction profiles of 440 genes and 14 antibiotic conditions (Fig. 6A). In a similarity network, genes displaying tightly correlated profiles form clusters, with the relative distance between clusters reflecting their functional relationships (*5*). Consistent with this, we found that genes within each individual biological process had high Pearson correlation coefficients (PCCs) with each other (Materials and Methods) and were densely interconnected (Fig. 6B). Furthermore, several processes, including CC, DD, coenzyme A synthesis, riboflavin metabolism, and protein export, formed a discernable supercluster, which we designated cluster I (Fig. 6B). In contrast, processes such as transcription (RNA polymerase), translation (ribosome), fatty acid biosynthesis, and ETC were distant from cluster I. To corroborate this network structure, we calculated the mean PCCs among essential genes within these processes (Materials and Methods). Hierarchical clustering of interprocess PCCs of a comprehensive set of core essential processes spanning diverse functions (Fig. 6C) recapitulated the gene-level functional distances captured in the similarity network (Fig. 6B).

Closely clustered genes and processes imply functional similarity. Genes with related functions tend to be enriched for synergistic GIs (*5*), a type of GI in which the combined perturbation of two genes results in a significantly greater fitness defect than expected from the individual perturbations. This is because multiple partial loss-of-function mutations in the same nonlinear pathway (especially essential ones) often push the pathway below a functional threshold, aggravating fitness.

**Fig. 7.**
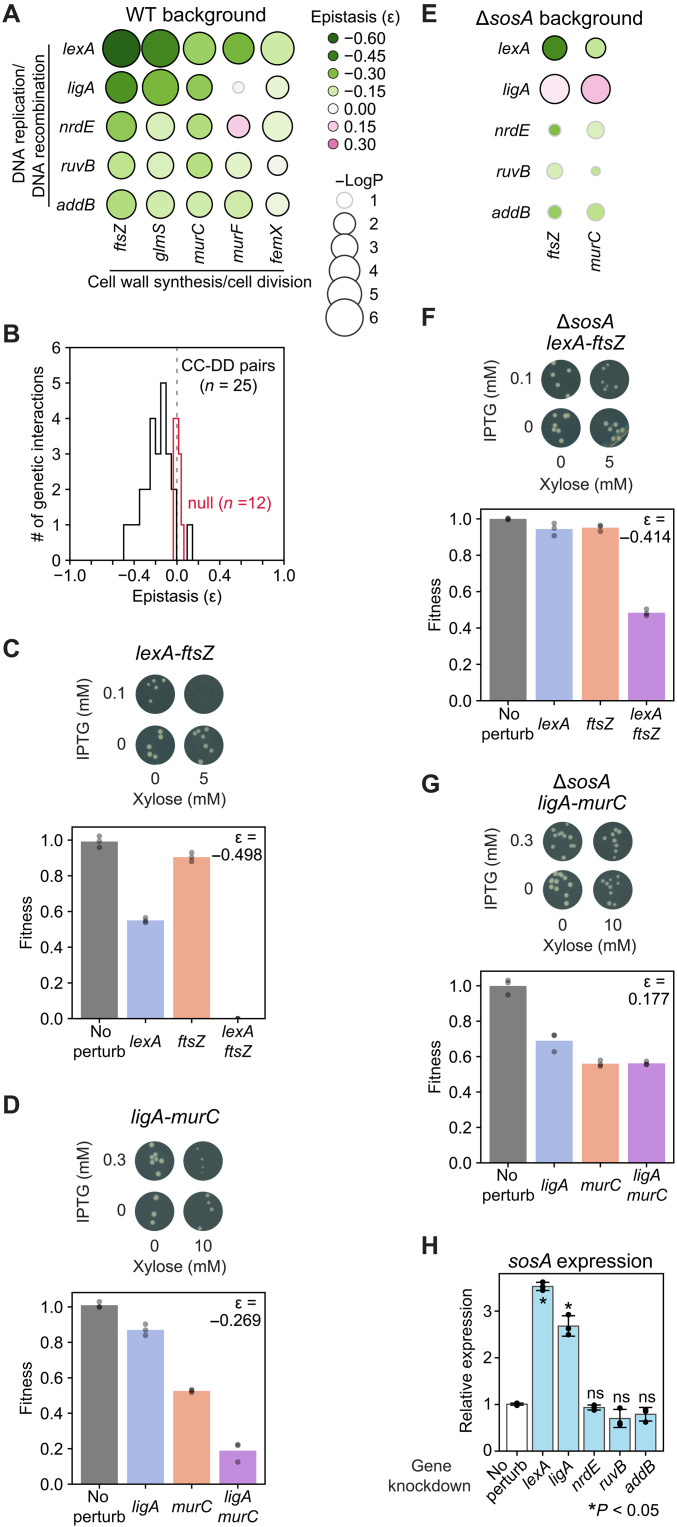
Synergistic GIs between CC and DD. (**A**) GI matrix between five CC and five DD genes in *S. aureus* RN4220, quantified as shown in fig. S8D. For each gene pair, epistasis is reported as the mean of three biological replicates. *P* values were calculated using a two-sample *t* test comparing triplicates of test gene-gene pair with those of the nontargeting control induced at the same IPTG and xylose concentrations (fig. S8F). Epistasis and *P* values are provided in table S16. (**B**) Distributions of GI (epistasis, ε) among 25 CC-DD gene pairs in (A) and baseline epistasis between plasmid backbones (null, *n* = 12) shown in fig. S8F. (**C**) GI between *lexA* and *ftsZ*. Top: Colonies of RN4220 cells carrying IPTG-inducible dCas9 targeting *lexA* and xylose-inducible asRNA targeting *ftsZ* plated on TSA containing indicated inducer concentrations. Bottom: Quantification of fitness (colony sizes in top panel) for bacteria targeting *lexA*, *ftsZ*, or both simultaneously, normalized to the no-perturbation control. Epistasis was calculated as ε_*lexA*,*ftsZ*_ = W_*lexA*, *ftsZ*_ - W*_lexA_* • W *_ftsZ_*, where W denotes fitness. ε is reported as the mean of three biological replicates. (**D**) Same as (C), showing the GI between *ligA* and *murC*. (**E**) GI matrix between CC-DD pairs in the Δ*sosA* background. Unlike (A), *P* values were calculated using a two-sample *t* test comparing the triplicates in the Δ*sosA* background with those in the WT background shown in (A). Circles with black edges indicate *P* < 0.05. See also fig. S8 (J and K). (**F** and **G**) same as (C), showing GIs in the Δ*sosA* background. (**H**) *sosA* expression quantified by reverse transcription quantitative polymerase chain reaction (means ± SD, *n* = 3). Genes were repressed by inducible CRISPRi for 1 hour before RNA extraction. A two-sample *t* test was performed between repressed gene and “no perturb” control.

The close proximity between genes involved in CC and those involved in DD within cluster I (Fig. 6B) suggested functional similarity and potential synergies between these processes. To test this hypothesis, we created a dual-plasmid system enabling inducible and orthogonal transcriptional repression (fig. S8A). By growing bacteria in varying concentrations of the two inducers, IPTG and xylose, we quantified fitness when pairs of CC and DD genes from 10 distinct operons (fig. S8B) were either individually or simultaneously repressed (fig. S8C). This allowed us to calculate GI, or epistasis (ε), which measures the deviation of the observed dual-perturbation effect from the expected effect based on individual perturbations (fig. S8D). We observed moderate to strong synergies (ε < −0.1 and *P* < 0.05) in 21 of 25 tested CC-DD combinations (Fig. 7, A and B), but no GIs in null controls (fig. S8, E to G). To contextualize the magnitude of these interactions, we referenced data from a comprehensive yeast global GI network (*5*), which showed that epistasis is generally infrequent (i.e., ε ≈ 0 for most GIs) and that |ε| = 0.1 represents more than 1 SD from the mean of ~600,000 GIs among essential genes (fig. S8H). As shown in Fig. 7A, we found that *lexA* strongly synergized with all five tested CC genes (−0.50 < ε < −0.15). These synergies were strong enough such that dual perturbations did not yield detectable colonies (Fig. 7C and *lexA* rows in fig. S8, C and D), even after prolonged incubation (first two panels in fig. S8I). Another example of strong synergy was observed between *ligA* and *murC* (Fig. 7D). On average, the five DD genes exhibited stronger synergies with *ftsZ* and early-stage peptidoglycan synthesis genes (*glmS* and *murC*) than with late-stage genes (*murF* and *femX*) (Fig. 7A). For several CC-DD pairs, the synergistic interactions were so pronounced that prolonged incubation still produced few or no colonies (arrows in fig. S8I). These results strongly indicate that simultaneous genetic perturbation of diverse CC and DD genes aggravates bacterial fitness, corroborating the CC-DD synergies observed in the context of antibiotic-gene interactions (Fig. 2).

What is the mechanism underlying CC-DD synergies? In *S. aureus*, DNA double-strand breaks trigger LexA degradation, which derepresses the SOS regulon (*50*), including the cell division inhibitor protein SosA (*51*), a functional analog of SulA. SulA binds to FtsZ and blocks cell division (*52*). We reasoned that repression of various DD processes could induce the SOS response, thereby activating SosA to inhibit cell division, which could explain the observed CC-DD synergies. To test this hypothesis, we generated a *sosA* deletion mutant and measured GIs between the five DD genes and two CC genes (*ftsZ* and *murC*). *sosA* deletion completely abolished the *ligA*-*ftsZ* and *ligA*-*murC* synergies (Fig. 7, E and G, and *ligA* row in fig. S8K). A strong effect was also observed for the *lexA*-*ftsZ* interaction: Simultaneous repression of the two genes in the Δ*sosA* background no longer prevented colony formation (Fig. 7F), a qualitative difference from the WT background (Fig. 7C), even though the quantified synergy was only modestly—yet significantly—reduced in the Δ*sosA* background (ε = −0.498 versus ε = −0.414, *P* < 0.05 in Fig. 7, C and F). In contrast, CC-DD interactions involving *nrdE*, *ruvB*, and *addB* showed ε values that were not statistically different between the WT and Δ*sosA* backgrounds (Fig. 7E). Together, these results indicate that *sosA* contributes to only a subset of CC-DD synergies, specifically those involving *ligA* and *lexA*. In contrast, CC-DD synergies involving *nrdE*, *ruvB*, and *addB* likely operate in a *sosA*-independent manner. Our reverse transcription quantitative polymerase chain reaction (qPCR) experiments showed that among the five DD genes, knockdown of only *ligA* or *lexA* led to significant upregulation of *sosA* expression (Fig. 7H), supporting the genetic results.

**Fig. 8.**
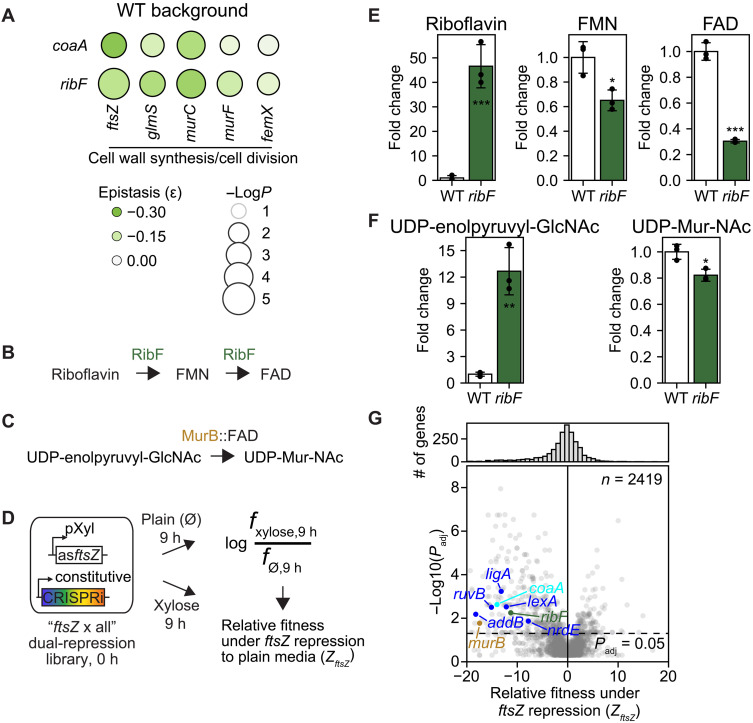
Other synergistic GIs involving CC. (**A**) GI matrix between five CC genes and *coaA* and *ribF* in *S. aureus* RN4220, quantified as shown in fig. S8 (L and M). For each gene pair, epistasis is reported as the mean of three biological replicates. *P* values were calculated using a two-sample *t* test comparing triplicates of test gene-gene pair with those of the nontargeting control induced at the same IPTG and xylose concentrations (fig. S8F). Epistasis and *P* values are provided in table S16. (**B**) RibF catalyzes the synthesis of FAD, an essential cofactor for flavoproteins. (**C**) MurB is a flavoprotein that converts UDP-enolpyruvyl-GlcNAc into UDP-Mur-NAc. (**D**) Generation of “*ftsZ* x all” dual-repression library and fitness quantification. *S. aureus* cells carrying a xylose-inducible (pXyl) antisense fragment targeting *ftsZ* (as*ftsZ*) and a constitutively expressed CALM-CRISPRi library were grown in plain or media containing xylose that mildly repressed *ftsZ*. Relative fitness of genes under *ftsZ* repression to plain media was quantified as Z*_ftsZ_*. (**E** and **F**) Quantification of riboflavin, FMN, FAD, UDP-enolpyruvyl-GlcNAc, and UDP-Mur-NAc in WT or *ribF*-repressed *S. aureus* cells (means ± SD) from three biological replicates by LC-MS/MS. A two-sample *t* test was performed between WT and *ribF*-repressed cells. *, **, and *** indicate *P* < 0.05, 0.01, and 0.001, respectively. (**G**) Volcano plot showing the fitness of genes under mild *ftsZ* repression relative to plain media, Z*_ftsZ_*. Z*_ftsZ_* values are shown in table S15.

Processes such as coenzyme A synthesis and riboflavin metabolism also clustered closely with CC (Fig. 6, B and C), suggesting synergistic GIs among them. This was validated by our dual-repression assays (Fig. 8A and fig. S8, L and M). To explore the mechanism underlying the synergies between CC genes and *ribF*, we noted that RibF catalyzes the synthesis of FAD (Fig. 8B), an essential cofactor for flavoproteins. MurB has been validated as a flavoprotein in *S. aureus* by multiple studies (*53*–*55*). Given MurB’s essential role in cell wall synthesis (Figs. 2A and 8C), its functional impairment due to RibF repression and FAD depletion likely contributes to the observed synergy between *ribF* and CC genes. To support this hypothesis, we generated an “*ftsZ* x all” dual-repression library and quantified the relative fitness of all genes under mild *ftsZ* repression (Fig. 8D). This global assay showed that among all known flavoprotein-encoding genes, only *murB* repression substantially reduced relative fitness when *ftsZ* was simultaneously repressed (fig. S9, A and B). Our metabolomics analysis further supported this hypothesis. RibF repression led to a significant accumulation of the enzyme’s substrate, riboflavin, and a concomitant decrease in its products, flavin mononucleotide (FMN) and FAD (Fig. 8E). Similarly, a significant accumulation of MurB’s substrate was accompanied by a decrease in its product (Fig. 8F). These results strongly indicate compromised MurB activity upon *ribF* repression. Last, the “*ftsZ* x all” global assay revealed that repression of *coaA*, *ribF*, and the five DD genes significantly reduced the relative fitness under mild *ftsZ* repression (Fig. 8G), further supporting their synergistic interactions with *ftsZ*. Collectively, our results uncovered synergistic GIs among essential bioprocesses such as CC, DD, and riboflavin metabolism and provided mechanistic insights into the basis of these interactions.

### Drug-drug synergies

**Fig. 9.**
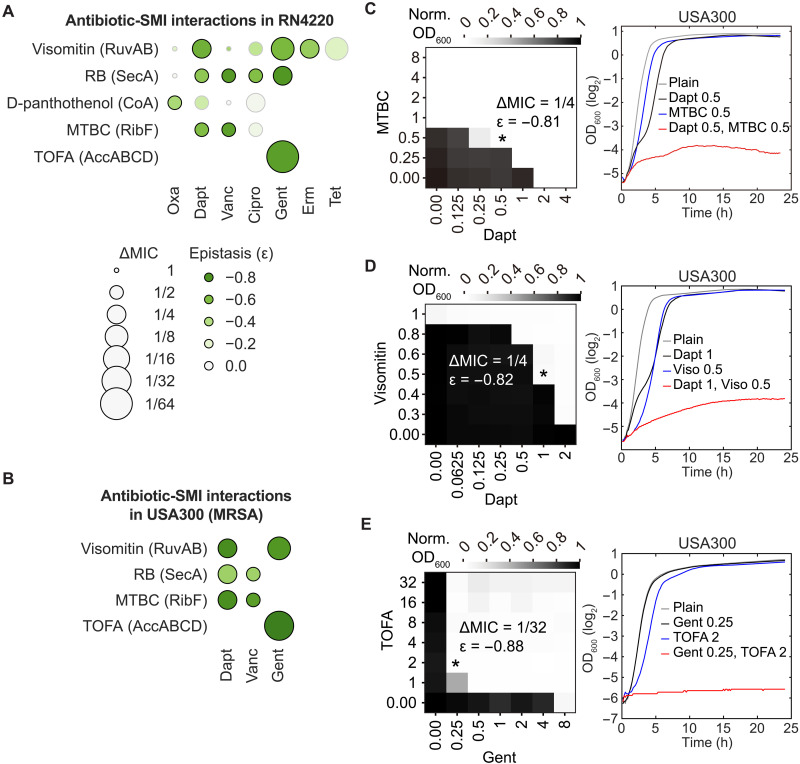
Drug-drug synergy. (**A**) Interactions between antibiotics (*x* axis) and SMIs (*y* axis) in *S. aureus* RN4220 quantified by checkerboard assays. Targets of SMIs are shown in parentheses. Changes in MIC (ΔMIC) and epistasis (ε) were quantified. Synergistic antibiotic-SMI combinations (ΔMIC ≤ ½ and ε ≤ −0.4) have black edges while other combinations have gray edges. RB, rose bengal; MTBC, methylbenzethonium chloride; TOFA, 5-(tetradecyloxy)-2-furoic acid. −log2(ΔMIC) and ε values are provided in table S17. (**B**) Same as (A) but showing antibiotic-SMI interactions in *S. aureus* USA300-LAC. (**C**) Left: Checkerboard assay of the daptomycin-MTBC combination in *S. aureus* USA300-LAC, showing bacterial growth at various antibiotic and SMI concentrations, normalized to growth in plain media (i.e., ${\text{OD}}_{\text{norm}}^{\text{abx},\;\text{SMI}}={\text{OD}}_{i}^{\text{abx},\;\text{SMI}}/{\text{OD}}_{i}^{\text{plain}}$) at 22 to 24 hours postinoculation. An asterisk (*) marks the well used to calculate the change in MIC (ΔMIC) and epistasis (ε) (Materials and Methods). Concentrations of the antibiotic and SMI are in micrograms per milliliter. Right: Four growth curves related to the asterisk-marked well: plain media (gray), antibiotic alone (black), SMI alone (blue), and antibiotic-SMI combination (red). (**D** and **E**) Same as (C) but showing the daptomycin-visomitin and gentamicin-TOFA combinations for *S. aureus* USA300-LAC, respectively.

To investigate whether we can translate synergistic GIs into viable combinatorial drug therapies, we identified small-molecule inhibitors (SMIs) targeting products of genes in cluster I (Fig. 6B), including RuvAB (*56*) (DNA recombination), SecA (*57*) (protein export), coenzyme A biosynthesis (*58*), and RibF (*59*) (riboflavin metabolism), and tested whether they could synergize with the antibiotics used in this study. Quantifying antibiotic-SMI interactions by solely measuring the changes in MIC (ΔMIC) at a terminal growth time point (~24 hours) could overestimate drug-drug interactions. To be more stringent, we also calculated epistasis (ε) between antibiotics and SMIs over an extended period of growth (Materials and Methods), and only considered a simultaneous reduction in MIC (ΔMIC ≤ ½) and strong negative epistasis (ε ≤ −0.4) to be synergistic antibiotic-SMI interactions. Of the 20 antibiotic-SMI combinations tested in *S. aureus* RN4220, 11 exhibited synergy (Fig. 9A and fig. S10, A to O). Critically, four SMIs also strongly synergized with antibiotics in *S. aureus* USA300-LAC (Fig. 9, B to E, and fig. S10, R to X), the parental strain of JE2 harboring multiple resistance determinants, including methicillin, erythromycin, and tetracycline. Daptomycin, an antibiotic commonly used for treating MRSA infections, strongly synergized with three SMIs targeting distinct processes (Fig. 9B), including methylbenzethonium chloride (MTBC) that targets RibF (Fig. 9C) and visomitin that targets RuvAB (Fig. 9D). Among all antibiotic-SMI interactions, the strongest synergy was observed between gentamicin and 5-(tetradecyloxy)-2-furoic acid (TOFA) (Fig. 9E), which targets the AccABCD complex (*60*) in the fatty acid synthesis pathway (Fig. 5A). This finding is consistent with our antibiotic-gene interaction results in RN4220 (Fig. 5, B and C) and our previous work (*15*). Together, these results demonstrate the power of CRISPRi functional genomics in identifying antibiotic-synergizing essential gene targets and the potential to translate them into effective drug combinations. However, we caution that drug-drug synergy can also arise from drug pleiotropy or from changes in cellular permeability that alter intracellular drug concentrations without affecting their targets (*61*, *62*). Consequently, observed drug-drug interactions may not always reflect GIs identified through functional genomics studies.

Last, it is noteworthy that many of our identified drug combinations, such as those involving MTBC (Fig. 9C) and visomitin (Fig. 9D), displayed stronger in vitro synergy than vancomycin/β-lactam (ΔMIC = ½, ε = −0.08 in fig. S10P) or daptomycin/β-lactam (ΔMIC = ^1^/4, ε = −0.11 in fig. S10Q), drug combinations used in the clinic to treat *S. aureus* bacteremia (*63*–*66*). However, the clinical use of antibiotic combinations is driven not only by drug-drug synergy but also by additional considerations such as resistance suppression and pharmacological properties.

## DISCUSSION

Motivated by the scarcity of systems-level studies examining gene vulnerability under antibiotic stress in *S. aureus*, we quantified global gene fitness across 10 antibiotics with various modes of action using our previously developed CALM-CRISPRi functional genomics platform. Our approach revealed a comprehensive set of biological processes affecting bacterial fitness under antibiotic stress (Fig. 1G). Essential genes dominated the hundreds of significant antibiotic-gene interactions that we identified (Fig. 1G and fig. S3), a finding not manifested by previous transposon-based studies. Recent CRISPRi work in *M. tuberculosis* and GI study in yeast also found that essential genes participated in significantly more antibiotic-gene (*6*) and gene-gene interactions (*5*) than nonessential genes, strengthening our findings in *S. aureus*. We note that while CRISPRi is well suited for quantifying the fitness effects of essential genes, recently developed inducible Tn-seq approach now allows sensitive measurement of essential-gene fitness as well (*67*). Moreover, Tn-seq remains a powerful method for identifying conditionally essential genes (*68*, *69*).

Inhibition of diverse essential biological processes can both sensitize and desensitize bacteria to antibiotics. Upon perturbation, essential bioprocesses such as CC, DD, coenzyme A biosynthesis, protein export, and riboflavin metabolism were among the most vulnerable under multiple antibiotic conditions, especially those targeting cell wall/membrane and DNA synthesis. Among these processes, we found genes such as *ruvB* and *rnjA* whose repression sensitized *S. aureus* to additional antibiotics that target translation (Fig. 2, G and K, and figs. S4I and S5Y), highlighting these gene products as attractive targets for combinatorial antimicrobial therapy. Perturbation of essential genes could also desensitize bacteria to antibiotics. Notably, CRISPRi repression of genes involved in transcription and translation antagonized bactericidal killing by many antibiotics, establishing a genetic basis for prior findings that pharmacological inhibition of transcription and translation slows cellular metabolism and reduces antibiotic lethality (*33*, *34*). We also found that repression of fatty acid biosynthesis and a clinical mutation affecting *fabF* desensitized bacteria to daptomycin (Fig. 5). While mutations in lipid biosynthetic enzymes such as *pgsA*, *cls*, and *mprF* have been associated with daptomycin resistance (*70*), alterations in fatty acid biosynthesis have not, to our knowledge, been linked to this phenotype, warranting further mechanistic investigation. Repression of pyruvate metabolism, ETC, and purine nucleotide biosynthesis pathways also desensitized bacteria to multiple antibiotics. This effect was further supported by our identification of abundant function-impairing mutations in these pathways in both laboratory evolution experiments and clinical isolates (fig. S7, B and C). Together, our CRISPRi screen systematically defines pathway-level vulnerabilities and tolerance mechanisms across antibiotic conditions, linking bacterial essential pathway perturbations to adaptive responses observed in both laboratory clinical settings.

The genome-scale antibiotic-gene interaction profiles enabled us to construct an essential gene similarity network (Fig. 6, A and B), which revealed important functional relationships among diverse biological processes and their underlying mechanisms. We observed extensive synergistic interactions between diverse CC and DD processes (Fig. 7, A and B). While previous mechanistic studies (*52*, *71*) focused on characterizing the connections between cell division (e.g., *sulA* and *ftsZ*) and DNA replication (e.g., *dnaA* and *dnaB*), the scale of our approach identified a more extensive CC-DD network encompassing genes in various stages of cell wall synthesis (e.g., *glmS* and *murC*), nucleotide synthesis (e.g., *nrdE*), and DNA recombination (e.g., *addB* and *ruvB*). Critically, our dual-perturbation assay provided direct experimental evidence that simultaneous transcriptional repression of these essential CC and DD genes aggravated bacterial fitness. Our genetic analysis further demonstrated that CC-DD synergies could be either dependent on, or independent of SosA, a functional analog of the cell division inhibitor SulA in *S. aureus* (Fig. 7E). SosA contributed significantly to CC-DD synergies involving *lexA* or *ligA*, which are genes whose perturbation also strongly induced SosA (Fig. 7H). In contrast, deletion of *sosA* did not significantly reduce CC-DD synergies involving the other three tested DD genes: *nrdE*, *ruvB*, and *addB*, suggesting that other pathways may underlie these interactions. An alternative explanation is that chromosome replication and cell division are two highly coordinated and essential processes in bacteria (*71*–*73*), and multiple partial loss-of-function mutations within this coordination may simply push the system below a functional threshold and aggravate fitness without necessarily involving additional pathways. An essential gene similarity network constructed in *B. subtilis* based on chemical-genomic screens (35 compounds × 289 genes) also revealed close proximity between CC and DD genes (*74*), supporting our results in *S. aureus*. Unlike these findings in bacteria, the comprehensive yeast GI study did not report prominent CC-DD synergies (*5*). This seems to be congruous with a fundamental distinction in eukaryotes, where chromosome replication (S phase) and cell division (M phase) are temporally separated. In addition to the extensive CC-DD synergies, we identified a strong synergy between RibF, which synthesizes the essential cofactor FAD, and the CC process (Fig. 8A), mediated by MurB—an essential flavoprotein involved in cell wall biosynthesis (Fig. 8F and fig. S9A).

The findings from our antibiotic-gene and gene-gene interaction profiling define a gene vulnerability landscape that can guide drug target discovery and the development of effective combination therapies against *S. aureus*. Of the 20 antibiotic-SMI combinations that we tested, 11 exhibited synergistic killing in vitro (Fig. 9, A and B), many of which showed stronger synergy than antibiotic combinations currently used in the clinic, such as daptomycin/β-lactam (*63*, *66*). Notably, antibiotic-SMI combinations involving visomitin, MTBC, and TOFA were highly synergistic when tested against MRSA USA300. Visomitin and MTBC target RuvAB and RibF, respectively, which are molecular targets that do not have approved antibiotics. Another potentially attractive drug target with no known inhibitors is ribonuclease J1, encoded by *rnjA*. Our results (figs. S4I and S5, N and Y), along with a previous study (*30*), show that *rnjA* repression sensitizes *S. aureus* and *B. subtilis* to antibiotics of various modes of action, including those targeting the cell wall/membrane, DNA synthesis, and translation. Collectively, these findings provide a strong rationale for developing and optimizing antimicrobial agents against these targets and for deploying them in combination therapies as a viable antimicrobial strategy.

Our small-scale similarity network analysis, based on 440 essential genes and 14 antibiotic conditions, uncovers important GIs within the core essential genome, as well as rational drug targets and drug synergies. These findings motivate more comprehensive investigations of GIs across the entire genome, which may further advance our understanding of bacterial genetic architecture and cellular organization and accelerate antimicrobial discovery. Excitingly, two recent studies used CRISPRi-based functional genomics to investigate GIs in *Streptococcus pneumoniae* (*75*), encompassing ~50% of possible digenic combinations, and in *B. subtilis* (*76*), focusing on digenic combinations involving genes in CC. We hope that our work, along with these GI studies, will inspire future efforts to explore biological principles in diverse bacterial species using high-throughput CRISPR-based genomics.

## MATERIALS AND METHODS

### Bacterial strains and reagents

Cultivation of *S. aureus* strains RN4220 (*20*), JE2 (*21*), and USA300-LAC (*21*) were carried out in TSB medium (BD) at 37°C with shaking (rpm 220), or on tryptic soy agar (TSA) plates. Whenever necessary, medium was supplemented with appropriate antibiotics to select for plasmid transformation. In these cases, the concentrations of antibiotics were as follows: chloramphenicol, 5 μg/ml; erythromycin, 10 μg/ml; tetracycline, 5 μg/ml. Antibiotics, SMIs, and key reagents used in the study are shown in table S20.

### Plasmid cloning

Plasmid pWJ584 was constructed by Gibson assembly of two PCR products. One PCR was performed using plasmid pWJ402 as template and primers W1956 and W1948. Another PCR was performed using pWJ420 as template and primers W1957 and W1961.

Plasmid pWJ587 (chloramphenicol-resistant) was constructed by Gibson assembly of two PCR products. One PCR was performed using plasmid pWJ418 as template and primers W1341 and W1342. Another PCR was performed using pWJ444 as template and primers W1971 and W1972. The canonical CRISPR array has the structure “repeat-spacer-repeat.” pWJ587 contained two Bsa I cleavage sites between the two repeats, facilitating spacer cloning. Spacer is the DNA precursor to crRNA.

Plasmid pWJ676 was constructed by Gibson assembly of three PCR products. The first PCR was performed using pT181 as template and primers W2326 and W666. The second PCR was performed using pWJ584 as template and primers W2327 and W2328. The third PCR was performed using pWJ584 as template and primers W2329 and W2331.

Similar to previous studies (*14*, *15*), CRISPR spacer cloning was performed by ligation of annealed oligonucleotide pairs and Bsa I–digested parent vector, pWJ444 (constitutive dCas9) or pWJ587 (IPTG-inducible dCas9). Plasmids pWJ402, pWJ418, pWJ420, and pWJ444 were previously published (*15*).

pWJ700 harbored a single repeat as opposed to repeat-spacer-repeat but was otherwise isogenic to pWJ587. To obtain pWJ700, we streaked RN4220 cells harboring an IPTG-inducible *ftsZ*-targeting CRISPRi plasmid, pWJ628, on TSA containing chloramphenicol (10 μg/ml) and 1 mM IPTG. As *ftsZ* is an essential gene, it was straightforward to isolate escaper cells containing mutated plasmid that had lost *ftsZ*-targeting spacer through homologous recombination of the flanking repeats. The CRISPR repeat region of pWJ700 was confirmed by Sanger sequencing.

To genetically complement *ruvB* and *ligA*, synonymous alleles resistant to CRISPRi targeting were expressed from a second plasmid. Plasmid pWL15, a *ruvB* complementation plasmid, was constructed via Gibson assembly of three PCR products. The first PCR product was generated using plasmid pWJ418 as template with primers W2286 and W2287. The second PCR product was generated using RN4220 genomic DNA as template with primers WL1 and WL4. The third PCR product was generated using RN4220 genomic DNA as template with primers WL2 and WL3. Similarly, plasmid pWL25, a *ligA* complementation plasmid, was constructed through Gibson assembly of three PCR products. The first PCR product was generated using plasmid pWJ418 as template with primers W2286 and W2287. The second PCR product was generated using RN4220 genomic DNA as template with primers WL5 and WL8. The third PCR product was generated using RN4220 genomic DNA as template with primers WL6 and WL7.

To perform GI assays, it was necessary to change the resistance of pWJ587-based plasmids targeting various genes from chloramphenicol to tetracycline. This was achieved by Gibson assembly of two PCR products. One PCR was performed using plasmid pT181 as template and primers W1311 and W1312. Another PCR was performed using the pWJ587-based plasmid as template and primers W2394 and W2395. For example, the chloramphenicol-resistant cassette of pWJ700 (single-repeat) was changed to tetracycline in this manner, generating pWL17.

Antisense RNA fragments targeting genes of interest were cloned after a xylose-inducible promoter in a pC194-based plasmid, pEPSA5 (*77*). To do so, pEPSA5 was linearized by EcoRI. Next, PCR was performed to amplify an antisense fragment targeting genes of interest in RN4220. The two fragments were ligated by Gibson assembly.

Sequences of CRISPR spacers, antisense fragments, and oligos used for cloning are listed in table S21. All PCRs and Gibson assembly reactions were performed using Q5 High-Fidelity DNA Polymerase and NEBuilder HiFi DNA Assembly Master Mix supplied by NEB, respectively.

### Construction of the Δ*sosA* and *fabF* P137L mutant strains

To delete the *sosA* (SAOUHSC_01334) in *S. aureus* RN4220, we took an allelic exchange approach facilitated by CRISPR counterselection. First, ~1-kb regions upstream and downstream of *sosA* were amplified by PCR using primer pairs WL97/WL98 and WL99/WL100, respectively. The plasmid backbone of vector pWJ244 (*78*) was amplified using primers W1005 and W1055. The three fragments were assembled via Gibson assembly to generate the allelic exchange plasmid pWL85, which was then transformed into *E. coli* DH5α for propagation and subsequently purified. Next, pWL85 was introduced into *S. aureus* RN4220, and transformants were selected on TSA containing chloramphenicol (10 μg/ml) at 37°C. As pWL85 did not contain *S. aureus* origin of replication, chloramphenicol-resistant clones were exclusively integrants. Next, to promote plasmid excision, we infected integrants with a temperature-sensitive, erythromycin-resistant phagemid pWJ326, which carries a CRISPR-Cas9 system targeting the chloramphenicol-resistant cassette on pWL85. Infected cells were plated on TSA containing erythromycin (5 μg/ml) and incubated at 30°C to select for plasmid excision. Last, successful deletion of *sosA* was confirmed by PCR using primer pairs WL101/W1043 and W614/WL102, and further validated by Sanger sequencing. The resulting RN4220Δ*sosA* strain was designated LIVA6.

To generate a *fabF* (SAOUHSC_00921) P137L point mutant in *S. aureus* RN4220, we used the same allelic exchange strategy described above. First, ~1-kb regions upstream and downstream of the point mutation were amplified by PCR using primer pairs WL120/WL121 and WL122/WL123, respectively. The plasmid backbone of vector pWJ244 was amplified using primers W1005 and W1055. The three fragments were assembled via Gibson assembly to generate the allelic exchange plasmid pWL93, which was then transformed into *E. coli* DH5α for propagation and subsequently purified. Next, the suicidal pWL93 was introduced into *S. aureus* RN4220, and integrants were selected on TSA containing chloramphenicol (10 μg/ml) at 37°C. Next, to promote plasmid excision, we infected integrants with a temperature-sensitive, erythromycin-resistant phagemid pWJ326, which carries a CRISPR-Cas9 system targeting the chloramphenicol-resistant cassette on pWL93. Infected cells were plated on TSA containing erythromycin (5 μg/ml) and incubated at 30°C to select for plasmid excision. Last, successful *fabF* P137L mutation was confirmed by PCR using primer pairs WL130/WL131 and further validated by Sanger sequencing. The resulting RN4220 *fabF* P137L strain was designated LIVA12.

### Determining sublethal antibiotic concentrations for CALM-CRISPRi screens

To determine the sublethal concentration of each antibiotic for the CALM-CRISPRi screens, we measured the growth of *S. aureus* in TSB containing a range of antibiotic concentrations. Figure S1A shows the twofold titration curves for bactericidal antibiotics (left column) and bacteriostatic antibiotics (right column). For each bactericidal antibiotic, we selected the highest concentration that still permitted viable cellular growth at 9 hours postinoculation [optical density at 600 nm (OD_600)_ > 1], which is the time point at which we harvest the CRISPRi library for sequencing analysis. Because the titration profiles differed across antibiotics, we selected two concentrations for several of them—daptomycin (0.25 and 0.5 μg/ml), vancomycin (0.5 and 1 μg/ml), ciprofloxacin (0.2 and 0.4 μg/ml), and gentamicin (0.5 and 1 μg/ml)—to maximize our ability to capture antibiotic-gene interactions. For bacteriostatic antibiotics, we chose concentrations that reduced exponential growth rates (that is, between OD_600_ of 0.125 and 0.5) to 50 to 83% of those measured in plain TSB.

### Generation of genome-scale CRISPRi libraries using CALM and subsequent selections in antibiotic conditions or xylose

Method for generating genome-scale CRISPRi libraries in *S. aureus* is similar to our previous study (*15*). The CALM-CRISPRi machinery is encoded on a single plasmid, pWJ584, which contains *tracr*, hd*cas9*, an empty CRISPR array (denoted as “R”), *cas1*, *cas2*, *csn2* (Fig. 1A), and a chloramphenicol-resistant cassette. The CRISPR adaptation machinery (*cas1*, *cas2*, and *csn2*) is under an IPTG-inducible promoter, pSpac. CRISPR components necessary for transcriptional repression (i.e., hdCas9, crRNA, and tracrRNA) are constitutively expressed. To start, a single colony of *S. aureus* RN4220 or JE2 strains harboring pWJ584 was grown overnight in 4 ml of TSB with chloramphenicol (5 μg/ml). Culture was diluted 1:200 in 15 ml of fresh TSB (no antibiotics) with 2 mM IPTG to induce the expression of Cas1, Cas2, and Csn2 and grown until OD_600_ reached 1.0 (typically 3 to 4 hours). To make competent cells, cells were pelleted and washed two times using one volume of sterile water at room temperature. Cells were ultimately resuspended in 1/100th volume of sterile water. It is crucial to perform these steps at room temperature and avoid cold shocking bacterial cells, as we observed that cells prepared at 4°C contained libraries with considerably more spacers matching the plasmid rather than the chromosome.

Competent cells (50 μl) were mixed with 2 μg of sheared genomic DNA (to an average of 150 bp) prepared from the same bacterial host and incubated 5 min at room temperature. Electroporation was performed using MicroPulser (Bio-Rad) with the default staph program (2 mm, 1.8 kV, and 2.5 ms). Notably, we found ~50% of the cells were killed by electroporation, which was moderate. After electroporation, cells were immediately resuspended in 500 μl of TSB and recovered at 37°C for 15 min with shaking. Next, 200 μl of recovered cells were transferred to 15 ml of prewarmed TSB with chloramphenicol (5 μg/ml) and recovered for an additional 5 hours at 37°C with shaking. During this recovery period, CRISPR adaptation and CRISPRi gene silencing occurred. The resulting culture, typically at 3 to 4 in OD_600_ (~3.0 or 10^9^ CFU/ml, constituted the genome-scale CRISPRi library we deemed as Time 0 hours in Fig. 1A. Typically, 7 ml of the library culture was pelleted, lysed, and amplified by PCR. Products were purified using either AMPure XP beads or Axygen AxyPrep MAG PCR Clean-Up Kit and sent for Illumina sequencing. We found the two types of beads to be interchangeable, as their size-selection performance was indistinguishable in our hands.

To profile the genetic fitness landscape of *S. aureus* in antibiotics, 7 ml of CALM-CRISPRi libraries were transferred to a 2-liter Erlenmeyer flask containing 700 ml of plain TSB (prewarmed at 37°C), or TSB containing sublethal concentrations of tested antibiotics, including oxacillin (0.05 μg/ml), daptomycin (0.25 and 0.5 μg/ml), vancomycin (1 μg/ml), ciprofloxacin (0.2 and 0.4 μg/ml), gentamicin (0.5 μg/ml), trimethoprim (0.4 μg/ml), erythromycin (0.08 μg/ml), tetracycline (0.04 μg/ml), linezolid (0.2 and 0.25 μg/ml), and mupirocin (0.02 μg/ml). Chloramphenicol was not added during this selection to avoid potential drug-drug interactions. Whenever daptomycin was used, media was also supplemented with 1 mM CaCl_2_. Cultures were incubated at 37°C with shaking (140 rpm) for 9 hours, reaching an OD_600_ of 4 to 5. Last, 7 ml of the culture was pelleted, lysed, amplified by PCR and sent for Illumina sequencing as described in section “*S. aureus* Harboring CRISPR Adaptation Machinery (hdcas9) Generates Single-spacer (1S) Libraries Targeting *S. aureus*” in a previous publication (*15*).

To generate the “*ftsZ* x all” dual-repression libraries, *S. aureus* RN4220 cells harboring pWJ633 (encoding an inducible antisense fragment targeting *ftsZ*) and pWJ676 (CALM) were used, following the same procedure as described for *S. aureus* carrying pWJ584. pWJ676 is identical to pWJ584, except that the chloramphenicol-resistance cassette is replaced with a tetracycline-resistance cassette. After constructing the “*ftsZ* x all” dual-repression library, 7 ml of the library culture was transferred to a 2-liter Erlenmeyer flask containing 700 ml of TSB containing chloramphenicol (5 μg/ml) (prewarmed at 37°C), or TSB containing chloramphenicol (5 μg/ml) plus 10 mM xylose. Chloramphenicol was used to maintain pWJ633, and xylose was used to induce antisense-*ftsZ* expression. Cultures were incubated at 37°C with shaking (140 rpm) for 9 hours, reaching an OD_600_ of 4 to 5, equivalent to ~7 generations. Last, 7 ml of the culture was pelleted, lysed, amplified by PCR and sent for Illumina sequencing as described in section “*S. aureus* Harboring CRISPR Adaptation Machinery (hdcas9) Generates Single-spacer (1S) Libraries Targeting *S. aureus*” in a previous publication (*15*).

### Sequencing analysis and gene fitness quantification

Illumina sequencing data was processed as described in section “Spacer identification and sequence alignment” under “Data Analysis of Single-spacer (1S) and “One-vs-all” Libraries” of a previous publication (*15*). Briefly, spacer sequences, which are the DNA precursors to crRNAs, were identified and aligned to reference genomes using a Burrows-Wheeler Alignment tool (*79*). For *S. aureus* RN4220, we used the *S. aureus* NCTC8325 reference genome (NCBI: NC_007795.1). A key genomic difference between RN4220 and its parental strain NCTC8325 is the absence of three prophage regions (SAOUHSC_01514 – SAOUHSC_01582, SAOUHSC_02015 – SAOUHSC_02089, and SAOUHSC_02164 – SAOUHSC_02239). For *S. aureus* JE2, we used *S. aureus* USA300_FPR3757 reference genome (NCBI: NC_007793.1), according to genome-scale alignment performed in a previous study (*21*).

For each sample, the number of reads for each spacer was recorded. Throughout this study, the terms “crRNA” and “spacer” are used interchangeably.

Our work quantified both gene fitness in plain TSB (*Z*_Ø_) and their relative fitness in antibiotics (*Z*_abx_). Quantification of gene fitness in plain TSB (*Z*_Ø_) is described as follows. For each CT crRNA, the normalized frequency of it, $f_{\text{sample}}^{\text{crRNA}}$, was calculated by dividing its reads by the total number of reads in that sample. The fitness of each crRNA in plain TSB media (Ø) was calculated as the log2-transformed fold change of frequency of crRNA measured at 9 hours to that measured at 0 hours

$${\text{log}}_{2}{\text{FC}}_{ø}^{\text{crRNA}}={\text{log}}_{2}\frac{f_{ø,9h}^{\text{crRNA}}}{f_{ø,0h}^{\text{crRNA}}}$$

crRNA positions with reads below 10 in both the numerator (i.e., 9-hour) and the denominator (i.e., 0-hour) were excluded from analysis. For each gene, we quantified ${\text{log}}_{2}{\text{FC}}_{ø}^{\text{gene}}$ by averaging all remaining CT crRNAs targeting that gene. To minimize potential off-target effects, we ranked all ${\text{log}}_{2}{\text{FC}}_{ø}^{\text{crRNA}}$s in ascending orders (i.e., from ${\text{log}}_{2}{\text{FC}}_{ø}^{{\text{crRNA}}_{1}}$ to ${\text{log}}_{2}{\text{FC}}_{ø}^{{\text{crRNA}}_{n}}$) and removed the CT crRNAs with the lowest and the highest values, unless the gene has three or fewer CT crRNAs. This gives

$${\text{log}}_{2}{\text{FC}}_{ø}^{\text{gene}}=\frac{{\text{log}}_{2}{\text{FC}}_{ø}^{{\text{crRNA}}_{2}}+{\text{log}}_{2}{\text{FC}}_{ø}^{{\text{crRNA}}_{3}}+\ldots +{\text{log}}_{2}{\text{FC}}_{ø}^{{\text{crRNA}}_{n-1}}}{n-2}$$

Subsequently, ${\text{log}}_{2}{\text{FC}}_{ø}^{\text{gene}}$ was standardized by *Z*-scoring, using pseudogenes made up by null crRNAs. Our CALM-CRISPRi platform routinely generated highly comprehensive crRNA libraries covering ~95% of all targetable sites in the genome, encompassing diverse genetic elements and intergenic regions. We considered sequences not annotated by NCBI reference genomes but are 100 bp upstream from operon start codons as intergenic regions. Approximately 4500 crRNAs targeting these intergenic regions were assigned as null crRNAs. For each treatment condition, 50 random null crRNAs were chosen to create a pseudogene, and fitness of the pseudogene, ${\text{log}}_{2}{\text{FC}}_{ø}^{\text{pseudo}}$, was calculated by averaging these null crRNAs. Next, a collection of 50 pseudogenes were created in this manner, and the mean and SD of 50 ${\text{log}}_{2}{\text{FC}}_{ø}^{\text{pseudo}}$s were calculated as $<{\text{log}}_{2}{\text{FC}}_{ø}^{\text{pseudo}}>$ and $\sigma_{ø}^{\text{pseudo}}$, respectively. This allowed us to calculate *Z*-scores for each gene

$$Z_{ø}^{\text{gene}}=\frac{{\text{log}}_{2}{\text{FC}}_{ø}^{\text{gene}}-<{\text{log}}_{2}{\text{FC}}_{ø}^{\text{pseudo}}>}{\sigma_{ø}^{\text{pseudo}}}$$

This standardized *Z*-score, serving as the final gene fitness score, allows samples to be compared across all tested conditions, including plain TSB media (Ø) and antibiotic treatments (abx). For each gene, a Mann-Whitney *U* test was performed between all CT crRNAs targeting the gene and the null crRNAs of the pseudogenes. The resulting *P* value is adjusted by the Benjamini-Hochberg method.

Similarly, the relative gene fitness in antibiotics compared to plain media can be also quantified as follows

$${\text{log}}_{2}{\text{FC}}_{\text{abx}}^{\text{crRNA}}={\text{log}}_{2}\frac{f_{\text{abx},\;9h}^{\text{crRNA}}}{f_{ø,9h}^{\text{crRNA}}}$$

$${\text{log}}_{2}{\text{FC}}_{\text{abx}}^{\text{gene}}=\frac{{\text{log}}_{2}{\text{FC}}_{\text{abx}}^{{\text{crRNA}}_{2}}+{\text{log}}_{2}{\text{FC}}_{\text{abx}}^{{\text{crRNA}}_{3}}+\ldots +{\text{log}}_{2}{\text{FC}}_{\text{abx}}^{{\text{crRNA}}_{n-1}}}{n-2}$$

$$Z_{\text{abx}}^{\text{gene}}=\frac{{\text{log}}_{2}{\text{FC}}_{\text{abx}}^{\text{gene}}-<{\text{log}}_{2}{\text{FC}}_{\text{abx}}^{\text{pseudo}}>}{\sigma_{\text{abx}}^{\text{pseudo}}}$$

Similarly, crRNA positions with reads below 10 in both the numerator (i.e., abx, 9-hour) and the denominator (i.e., Ø, 9-hour) were excluded from analysis. Throughout, $Z_{ø}^{\text{gene}}$ and $Z_{\text{abx}}^{\text{gene}}$ have been simplified as *Z*_Ø_ and *Z*_abx_, respectively.

In this study, we generated at least seven independent CALM-CRISPRi libraries in *S. aureus* RN4220. Libraries C2821, C2924, and C2998 (fig. S1E) were chosen as the three biological replicates to calculate mean *Z*_Ø_ values.

### Quantification using NCT crRNAs

We also quantified gene relative fitness in antibiotics using the mildly targeting NCT crRNAs (table S4). For many CT crRNAs targeting highly essential genes, such as those involved in ribosome, RNA polymerase, and fatty acid synthesis, their raw sequencing counts at 9-hour dropped to a very small number (<10 reads) in both plain media and antibiotic conditions (Fig. 1A), preventing reliable quantification of *Z*_abx_ (e.g., *P*_adj_ = 1 for many ribosomal genes in fig. S6A). In contrast, the mild NCT crRNAs did not severely deplete at 9 hours, offering a better dynamic range for quantifying the relative fitness of these highly essential genes in antibiotics. We used NCT crRNAs to quantify a portion of highly essential genes encoding the ribosome (Fig. 4B), RNA polymerase (*rpoA* and *SAOUHSC_01036* in Fig. 4A), and fatty acid synthesis (*acpPS*, *accABCD*, *fabI*, and *SAOUHSC_01196* in Fig. 3J).

The general flow of quantification is the same as before except that NCT, instead of CT crRNAs were used for each gene

$${\text{log}}_{2}{\text{FC}}_{\text{abx}}^{\text{NCT}\;\text{crRNA}}={\text{log}}_{2}\frac{f_{\text{abx},\;9h}^{\text{NCT}\;\text{crRNA}}}{f_{ø,9h}^{\text{NCT}\;\text{crRNA}}}$$

$$\begin{array}{c} {\text{log}}_{2}{\text{FC}}_{\text{abx}}^{\text{gene}\left(\text{NCT}\right)}\\ =\frac{{\text{log}}_{2}{\text{FC}}_{\text{abx}}^{\text{NCT}\;{\text{crRNA}}_{2}}+{\text{log}}_{2}{\text{FC}}_{\text{abx}}^{\text{NCT}\;{\text{crRNA}}_{3}}+\ldots +{\text{log}}_{2}{\text{FC}}_{\text{abx}}^{\text{NCT}\;{\text{crRNA}}_{n-1}}}{n-2} \end{array}$$

$$Z_{\text{abx}}^{\text{gene}\;\left(\text{NCT}\right)}=\frac{{\text{log}}_{2}{\text{FC}}_{\text{abx}}^{\text{gene}\;\left(\text{NCT}\right)}-<{\text{log}}_{2}{\text{FC}}_{\text{abx}}^{\text{pseudo}}>}{\sigma_{\text{abx}}^{\text{pseudo}}}$$

### Functional enrichment analysis

Functional enrichment analysis was performed using iPAGE, an information-theoretic pathway analysis tool (*80*) that we previously developed. We used both KEGG pathway (www.genome.jp/kegg-bin/show_organism?menu_type=pathway_maps&org=sao) and GO term annotations (https://biocyc.org/GCF_000013425/organism-summary) for *S. aureus* NCTC8325, a parental strain for RN4220. Overall, ~37 and ~83% of the RN4220 genome are annotated by KEGG pathways and GO terms, respectively. While *S. aureus* genes are poorly annotated by KEGG pathways (e.g., well known genes such as *ftsZ*, *lexA*, and *addAB* have no annotated pathways), GO terms tend to over-annotate, which can also lead to dilution of significant pathways. Discrete mode of iPAGE was used for enrichment analysis. Genes with *Z*_abx_ ≤ 9 and *P*_adj_ < 0.05 were categorized as strongly decreased relative fitness in antibiotics, while those with *Z*_abx_ ≥ 9 and *P*_adj_ < 0.05 were categorized as strongly increased relative fitness in antibiotics. For antibiotic conditions with biological replicates, mean *Z*_abx_ values were used for enrichment analysis.

### Pairwise competition assay

*S. aureus* RN4220 or JE2 cells harboring an IPTG-inducible dCas9, along with no spacer (pWJ700) and spacers targeting genes of interest were cloned. Whenever possible, we chose to validate genes that are the last in operons to avoid polar effects of CRISPRi. All spacers were cloned into the pWJ587 backbone with a chloramphenicol-resistant (cm^R^) marker.

Pairwise competition assays were performed to calculate the fitness of RN4220 or JE2 carrying spacers targeting genes of interest (candidates), each relative to the corresponding parental strain carrying no spacer (i.e., pWJ700), which served as the common competitor. First, candidate and the common competitor strains were streaked on TSA plates supplemented with chloramphenicol (5 μg/ml) and incubated at 37°C for 14 to 20 hours. Equal amount of candidate the common competitor cells were mixed by picking and resuspending single colonies in 1× phosphate-buffered saline (PBS) to an OD_600_ of 0.1 measured by plate reader. Mixed bacteria (2 μl) were inoculated into 200 μl of TSB containing chloramphenicol (5 μg/ml), IPTG, and testing antibiotics whenever necessary. Microplates were grown at 37°C with shaking (548 cpm) for 20 to 24 hours. After growth, 150 μl of bacteria in each condition was transferred to an 1.5-ml Eppendorf tube, pelleted, and washed with 1× PBS. As candidate genes were essential, it was necessary to choose IPTG concentrations that reduced the fitness of these genes by 50 to 90% relative to that of the common competitor under no antibiotic treatment.

To quantify the ratio of candidate and common competitor, pelleted cells were lysed, minipreped, and subjected to PCR. For each PCR, a 20-μl reaction mix was prepared by adding ~20 ng of plasmid DNA as template, 0.2 μM forward primer (W1201), 0.2 μM reverse primers (L401), and One*Taq* DNA polymerase (NEB). DNA bands were visualized on 2% agarose gel containing ethidium bromide. Intensity of DNA bands corresponding to spacers of interest (211 bp) and the common competitor (empty array, 145 bp) were quantified by ImageJ. An example of *ftsZ* is shown in fig. S1I. At the beginning and end of pairwise competition assay, we also spotted bacteria on TSA plates containing chloramphenicol (5 μg/ml) and incubated at 37°C for 14 to 20 hours. Combined with the ratios obtained from PCR, it allowed us to determine the initial and final population size of the candidate and the common competitor. Thus, fitness of candidate strain with spacer targeting gene *X* ($W_{X}$) relative to that of the common competitor (*81*, *82*) can be calculated as

$$W_{X}=\frac{\text{ln}\left(\frac{N_{X,f}}{N_{X,i}}\right)}{\;\text{ln}\left(\frac{N_{C,f}}{N_{C,i}}\right)}$$

where $N_{X}$ and $N_{C}$ are the population sizes of gene *X* strain and the common competitor, and subscripts *f* and *i* indicate the final and initial time points, respectively.

We also performed a mock pairwise competition between RN4220 carrying pWJ587 (WT) and RN4220 carrying pWJ700 (the common competitor) under plain and various antibiotic conditions. Essentially, two WT strains were being competed. As expected, the relative fitness of WT cells under various antibiotic conditions were not significantly different from that in plain media (fig. S1J).

### Checkerboard assays for measuring MIC

A single colony (10^6^ to 10^7^ CFUs) of *S. aureus* RN4220 cells carrying a chloramphenicol-resistant plasmid encoding IPTG-inducible dCas9 and a spacer targeting gene of interest were resuspended in 100 μl of 1× PBS. After serial dilution, ~50 to 200 CFUs were spotted on 5 × 4 TSA plates containing chloramphenicol (5 μg/ml) (to keep the CRISPRi plasmid targeting genes of interest) and various concentrations of IPTG and testing antibiotics. The checkerboard format was necessary as a range of IPTG concentrations was needed to capture the optimal transcriptional repression levels for different essential genes. Typically, TSA was supplemented with following IPTG concentrations: 0.003, 0.01, 0.03, and 0.1 mM.

To determine MIC values for essential genes, we first identified the highest IPTG concentration that still permitted colony formation in the absence of antibiotic. Next, we examined the row corresponding to this IPTG concentration on the checkerboard and identified the lowest antibiotic concentration at which plating efficiency decreased by ≥100-fold relative to the “no-antibiotic / no-IPTG” plate. This 100-fold cutoff was chosen to account for occasional CRISPR escape events. Sanger sequencing was routinely performed to confirm CRISPR escaper mutants.

To complement *ruvB*, RN4220 cells carrying dual plasmids pWJ658 (a chloramphenicol-resistant plasmid encoding IPTG-inducible dCas9 and a spacer targeting *ruvB*) and pWL15 (a tetracycline-resistant plasmid encoding IPTG-inducible *ruvB* containing synonymous mutations that negate CRISPRi targeting) were spotted on TSA plates containing chloramphenicol (5 μg/ml), tetracycline (2.5 μg/ml), appropriate amount of IPTG, and testing antibiotics. Similarly, *ligA* complementation was performed using RN4220 cells carrying plasmids pWJ653 and pWL25. TSA plates were incubated at 37°C for 24 or 48 hours as indicated in figures.

### Quantification of viability under antibiotic conditions

Ten-fold serial dilutions of *S. aureus* cells were spotted onto plain TSA or TSA supplemented with appropriate antibiotics. When necessary, TSA was supplemented with chloramphenicol (5 μg/ml) to maintain the plasmid-encoding CRISPRi system targeting genes of interest when necessary. Plates were incubated at 37°C for 18 to 24 hours. Viability was quantified as the ratio of CFUs on antibiotic-containing TSA to CFUs on plain TSA.

### Identifying mutations in NCBI Pathogen Detection database

The NCBI Pathogen Detection database (www.ncbi.nlm.nih.gov/pathogens/) was accessed in November 2025, and 4284 completely assembled *S. aureus* genomes were downloaded using the command <datasets download genome taxon “*Staphylococcus aureus*” --assembly-level complete --include gff3,genome,protein>. For genomes lacking protein sequence files (i.e., faa files), Prokka (*83*) was used to annotate the genomes and generate protein predictions. Truncated proteins were identified by detecting premature stop codons using getorf (*84*). For each protein of interest, sequences from all *S. aureus* genomes were aligned using MAFFT (*85*), with strain NCTC8325 used as the reference. Custom python scripts were then used to identify mutations at specific protein positions. Accession numbers and genomes with identified mutations are provided in table S18.

### Network analysis

To construct an essential gene similarity network in *S. aureus* RN4220 (Fig. 6, A and B), we analyzed genes that were targeted by at least two crRNAs, and were classified as essential by either Santiago’s Tn-seq study (*22*) or Bae’s (*24*) Tn-seq study. Pairwise PCCs between these essential genes were calculated on the basis of their relative fitness in 14 antibiotic conditions: oxa 0.05, dapt 0.25, dapt 0.5, vanc 1, cipro 0.2, cipro 0.4, tmp 0.4, gent 0.5, gent 1, erm 0.08, lzd 0.2, lzd 0.25, tet 0.04, and mup 0.02 (for antibiotic conditions with biological replicates, mean *Z*_abx_ values were used). This analysis generated a 440 × 440 gene-gene correlation matrix (table S12), which was used to construct the gene similarity network using the Kamada-Kawai algorithm. The threshold of PCC used was 0.65. To gain process-level insights, the mean of pairwise PCCs of essential genes from any two biological processes were calculated and subjected to hierarchical clustering (Fig. 6C).

### Gene-gene interaction measurement

To measure GIs, *S. aureus* strains RN4220 or RN4220Δ*sosA* carrying dual-plasmid systems were used. A chloramphenicol-resistant plasmid driving a xylose-inducible antisense RNA fragment was used to repress CC genes. Another tetracycline-resistant plasmid driving an IPTG-inducible dCas9 and crRNA was used to repress DD genes, *coaA*, and *ribF*. For each dual-gene combination, 5 to 10 single colonies of *S. aureus* RN4220 carrying dual-plasmid systems were resuspended in 1× PBS. Serial dilutions were prepared and appropriate numbers of CFUs were plated on TSA containing chloramphenicol (5 μg/ml), tetracycline (2.5 μg/ml), and various combinations of IPTG and xylose. TSA plates were incubated for 22 hours at 37°C, after which colony areas were quantified using ImageJ as a proxy for bacterial fitness. For each gene, we selected inducer concentrations that reduced fitness to 20 to 90% of unperturbed cells for subsequent quantification. For each gene pair (gene A and gene B), the fitness of individual gene perturbations and dual gene perturbation were normalized to that of cells with no perturbation, generating W_A_, W_B_, and W_A, B_, respectively. GI, or epistasis, was calculated as ε_A,B_ = W_A, B_ - W_A_ • W_B_. As controls, the fitness of bacteria carrying a nontargeting two-plasmid system (i.e., parental plasmids pC194 and pWL17) in the presence of various combinations of IPTG and xylose were also measured, allowing for the calculation of baseline epistasis. To determine the statistical significance of GIs, a two-sample *t* test between triplicates of test gene-gene pairs (ε_A,B_) and triplicates of nontargeting two-plasmid system induced in the same IPTG and xylose concentrations (ε_pC194,pWL17_) were performed.

### RNA extraction, reverse transcription, and qPCR

*S. aureus* RN4220 strains harboring an IPTG-inducible CRISPRi system targeting DD genes or a nontargeting control were grown overnight on TSA plates containing tetracycline (5 μg/ml) at 37°C. The next day, ~100 colonies were pooled and inoculated into fresh TSB supplemented with 1 mM IPTG to induce CRISPRi repression of target gene, followed by incubation with shaking (220 rpm, 37°C) for 1 hour. Approximately 10 units of cells (one unit is defined as 1 ml of bacterial culture at an OD_600_ of 1) were harvested by centrifugation at 5000*g* for 5 min at room temperature, resuspended in RNAlater Stabilization Solution (Invitrogen), incubated at room temperature for 30 min, and stored at 4°C until RNA extraction.

RNAlater-treated cell pellets were transferred into a 2-ml bead-beating tube preloaded with 0.1-mm glass beads. Cells were resuspended in 500 μl of Buffer RLT (RNeasy Mini Kit, Qiagen) freshly supplemented with 1% β-mercaptoethanol (10 μl/ml buffer) and vortexed briefly (5 to 10 s). Tubes were processed in a bead beater at 30.0 m/s for 30 s, four times, with samples kept on ice for 30 s between cycles to prevent overheating. Lysates were clarified by centrifugation at 13,000*g* for 1 min at 4°C, and the supernatants were carefully transferred to fresh ribonuclease-free tubes without disturbing beads or debris. The clarified lysates were then subjected to RNA purification using the RNeasy Mini Kit (Qiagen), followed by in-solution deoxyribonuclease I treatment to remove genomic DNA. RNA integrity was assessed using a TapeStation system (Agilent), and only samples with RNA integrity number (RIN) > 8 were used for downstream analyses.

cDNA was synthesized from 500 ng to 1 μg of total RNA using Maxima H Minus Reverse Transcriptase (Thermo Fisher Scientific) with random hexamer primers, according to the manufacturer’s protocol. To quantify the expression of *sosA* (SAOUHSC_01334), qPCR was performed using primers WL83 and WL84 and Power SYBR Green PCR Master Mix (Applied Biosystems) on a QuantStudio 6 Real-Time PCR System (Applied Biosystems). Primers W1643 and W1644 were used to amplify the housekeeping *gmk* (SAOUHSC_01176). For each sample, relative expression of *sosA* was calculated using ΔCt = Ct_sosA_ − Ct_gmk_. The fold change of *sosA* expression in experimental strain compared to control unperturbed strain was calculated as 2^−ΔΔCt^, where ΔΔCt = ΔCt_experimental_ − ΔCt_control_. All primers were validated for efficiency and linear range of amplification using standard qPCR approaches. Specificity was confirmed through melting curve analysis.

### Targeted metabolomic analysis by hydrophilic interaction liquid chromatography–tandem mass spectrometry

*S. aureus* JE2 carrying an IPTG-inducible CRISPRi system targeting *ribF* (pWJ643) or a nontargeting control (pWJ700) were grown overnight on TSA plates containing chloramphenicol (10 μg/ml) at 37°C. The next day, single colonies were picked and resuspended in PBS to a final OD_600_ of ~2.4. A total of 100 μl of the bacterial suspension was evenly spread on a TSA plate using a sterile loop and incubated at 37°C for 5 hours. After incubation, bacteria were collected from the plates using a sterile loop and resuspended in 1 ml of PBS. One unit of cells (defined as 1 ml of bacterial suspension at OD_600_ of 1) were harvested by centrifugation at 13,000 rpm for 2 min at room temperature and removing the supernatant.

To extract metabolites, cells were resuspended in 1 ml of ice-cold 80:20 (v/v) methanol:water. For each sample, ~2 × 10^7^ cells were lysed using ceramic bead tubes with 10 cycles of 15 s shaking at 600 m/s on an Omni Bead Ruptor homogenizer. Lysates were centrifuged at 20,000*g* for 20 min at 4°C, and the resulting supernatants were transferred to fresh tubes. Extracts were dried under vacuum using a Genevac EZ-2 Plus evaporator for 2 hours. Dried samples were reconstituted in 50 μl of 50% (v/v) acetonitrile, vortexed, incubated on ice for 20 min, and clarified by centrifugation at 20,000*g* for 20 min at 4°C before liquid chromatography–mass spectrometry (LC-MS) analysis. Residual double-stranded DNA content was quantified from an aliquot of each reconstituted extract using a NanoDrop spectrophotometer and used for sample normalization.

Metabolite detection (Riboflavin, FMN, FAD, UDP-GlcNAc-enolpyruvate, and UDP-MurNAc) was carried out with hydrophilic interaction liquid chromatography (HILIC) using an ultrahigh-performance liquid chromatography (Vanquish Horizon, Thermo Fisher Scientific) coupled to an Orbitrap IQ-X Tribrid mass spectrometer (Thermo Fisher Scientific) operated in heated electrospray ionization (HESI) mode. The column was a Waters Atlantis Premier BEH Z-HILIC (2.1 mm by 150 mm, 1.7 μm). The experimental samples (2 μl) were injected. The oven temperature was maintained at 30°C, and the flow rate was set to 0.2 ml/min. Chromatographic separations were performed by the following parameters: Solvent A consisted of water with 10 mM ammonium carbonate, and solvent B consisted of 95% acetonitrile and 5 μM medronic acid (InfinityLab deactivator additive, Agilent no. 5191-4506). A gradient run was set up as 0 to 10 min from 80 to 50% B, 10 to 11 min from 50 to 80% B, and 11 to 20 min re-equilibrate at 80% B. MS analyses were carried out with a HESI source operated in negative polarity mode. The source conditions were set as follows: spray voltage, static; sheath gas flow rate, 20 arbitrary units; aux gas flow rate, 4 arbitrary units; sweep gas flow rate, 3 arbitrary units; capillary temp, 300°C; vaporizer temp 200°C; RF lens 60%. Full-scan MS^1^ spectra were acquired in the Orbitrap analyzer at a resolution of 240,000, with an AGC target of 4 × 10^5^, maximum injection time set to auto, and a scan range of 100 to 800 mass/charge ratio (m/*z*) in profile mode. Targeted MS^2^ acquisition was subsequently performed using a parallel reaction monitoring (PRM) strategy. A predefined inclusion list containing the expected precursor ions corresponding to the [M–H]^−^ species of riboflavin (m/*z* 375.131), FMN (m/*z* 455.0973), FAD (m/*z* 784.1499), UDP-GlcNAc-enolpyruvate (m/*z* 676.0798), and UDP-MurNAc (m/*z* 678.0954) was used to trigger MS^2^ scans. Retention time scheduling for PRM acquisition was established on the basis of injections of authentic chemical standards when available and further refined by consistent detection of the target ions in pooled quality control samples analyzed throughout the batch. Precursor ions were isolated using a 1.0 m/*z* isolation window and fragmented by higher-energy collisional dissociation using normalized collision energies. MS^2^ spectra were acquired in the Orbitrap at a resolution of 120,000, with an AGC target of 2 × 10^5^, maximum injection time set to 200 ms, and a scan range of 100 to 800 m/*z* in profile mode. Mass accuracy was maintained within ±5 ppm for both precursor and fragment ions. For UDP-GlcNAc-enolpyruvate and UDP-MurNAc, metabolite identities were assigned on the basis of a combination of exact mass, chromatographic retention behavior, and diagnostic MS^2^ fragmentation patterns specific for diphosphate backbone, GlcNAc sugar fragments, and MurNac sugar-lactyl fragments. Riboflavin, FMN, and FAD were analyzed using the same chromatographic conditions and PRM-based MS^2^ acquisition strategy. For these metabolites, compound identities were confirmed by matching exact mass, retention time, and MS^2^ fragmentation patterns to those obtained from authentic chemical standards. Data analysis, including fragment ion extraction and peak area integration, was performed using Skyline-daily v24.1.1.398.

### Checkerboard assays for measuring antibiotic-SMI interactions

Synergy H1 microplate reader (Biotek) was used to perform checkerboard assays. Typically, Corning 3370 Clear Polystyrene 96-Well Microplates were used to harbor a 7 by 7 checkerboard containing 200 μl of TSB with varying concentrations of antibiotics and SMIs. To demonstrate the potentiation of antibiotics by SMIs, it was necessary to use the concentrations of antibiotics, but not those of the SMIs, in twofold serial dilutions. Five to 10 single colonies of *S. aureus* RN4220 or USA300-LAC cells were resuspended in 200 μl of 1× PBS. Resuspended cells (2 μl) were inoculated into 200 μl of media in each well of the checkerboard. Microplates were grown at 37°C with shaking (548 cpm) for 22 to 24 hours. The absorbance at 600 nm (OD_600_) was measured every 10 min.

We used fig. S10A as an example to illustrate how antibiotic-SMI interactions are quantified. First, for each time point, bacterial growth in various concentrations of antibiotic and SMI was normalized to bacterial growth in plain media (i.e., ${\text{OD}}_{\text{norm}}^{\text{abx},\;\text{SMI}}={\text{OD}}_{i}^{\text{abx},\;\text{SMI}}/{\text{OD}}_{i}^{\text{plain}}$). Panel (i) shows normalized bacterial growth at the terminal time point (22 to 24 hours postinoculation). An asterisk “*” labels the well where the change in MIC (ΔMIC) was determined. However, it is well known that terminal-point MIC determination can sometimes overestimate drug-drug interactions. To be more stringent, we adopted an additional epistasis metric from a recent large-scale drug-drug interaction study in bacteria (*62*), with modifications. Panel (ii) shows the four growth curves related to the well labeled with asterisk: plain media (gray), antibiotic alone (black), SMI alone (blue), and both antibiotic and SMI combined (red). For each time point along these growth curves, epistasis between antibiotic and SMI (ε^abx, SMI^) was calculated using a Bliss interaction model: $\varepsilon_{i}^{\text{abx},\;\text{SMI}}={\text{OD}}_{\text{norm}}^{\text{abx},\;\text{SMI}}-\left({\text{OD}}_{\text{norm}}^{\text{abx}}•{\text{OD}}_{\text{norm}}^{\text{SMI}}\right)$. For the well where ΔMIC was determined (i.e., “*”), we calculated ε^abx, SMI^ at 1-hour intervals, from 6 hours (when bacteria in plain media entered stationary phase) to 24 hours, the terminal time point. These values were averaged to yield the final ε, which quantifies the level of antibiotic-SMI interaction.

By quantifying ε^abx, SMI^ over an extended period of bacterial growth, the epistasis metric effectively compliments the biased MIC determination on the basis of a single time point. For example, D-pantothenol and cipro seemingly synergized as D-pantothenol reduced the MIC of cipro by fourfold in the 24-hour checkerboard result (fig. S10L). However, this proved to be an overestimation, as growth curves clearly revealed that bacteria only began growing in D-pantothenol (blue curve) at a late time point. Epistasis measurements showed that the average ε^abx, SMI^ from 6 to 24 hours was −0.06, indicating that the synergy was rather weak. We only considered a simultaneous reduction in MIC and a strong negative epistasis (ΔMIC ≤ ½ and ε ≤ −0.4) to be a synergistic antibiotic-SMI interaction.
