# Supplementary material for: The genetic landscape of antibiotic sensitivity in Staphylococcus aureus
Source: Sci Adv. 2026 May 8;12(19):eaeb9875. doi: 10.1126/sciadv.aeb9875 (PMC13162198; doi:10.1126/sciadv.aeb9875)
Supplement: Supplementary file 1 — Figs. S1 to S10 Legends for tables S1 to S21 [file sciadv.aeb9875_sm.pdf]

Science Advances
AAAS

Supplementary Materials for

The genetic landscape of antibiotic sensitivity in *Staphylococcus aureus*

Wan Li *et al.*

Corresponding author: Saeed Tavazoie, st2744@columbia.edu; Wenyan Jiang, wenyan.jiang@mssm.edu

Sci. Adv. **12**, eaeb9875 (2026)
DOI: 10.1126/sciadv.aeb9875

The PDF file includes:

Figs. S1 to S10
Legends for tables S1 to S21

Other Supplementary Material for this manuscript includes the following:

Tables S1 to S21

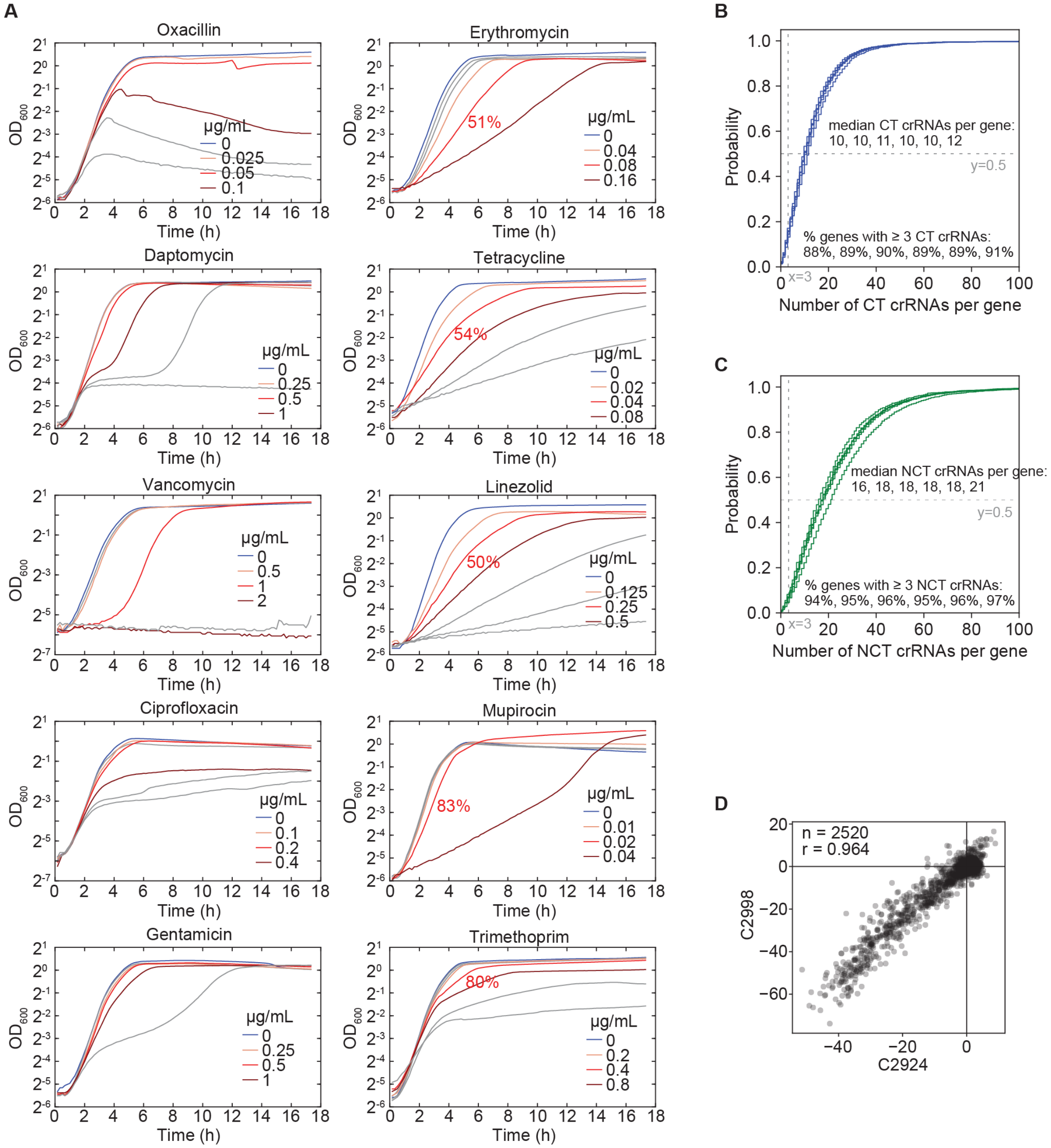

A
Oxacillin
µg/mL
0
0.025
0.05
0.1
Erythromycin
51%
µg/mL
0
0.04
0.08
0.16
Daptomycin
µg/mL
0
0.25
0.5
1
Tetracycline
54%
µg/mL
0
0.02
0.04
0.08
Vancomycin
µg/mL
0
0.5
1
2
Linezolid
50%
µg/mL
0
0.125
0.25
0.5
Ciprofloxacin
µg/mL
0
0.1
0.2
0.4
Mupirocin
83%
µg/mL
0
0.01
0.02
0.04
Gentamicin
µg/mL
0
0.25
0.5
1
Trimethoprim
80%
µg/mL
0
0.2
0.4
0.8
OD_{600}
Time (h)
B
Probability
median CT crRNAs per gene:
10, 10, 11, 10, 10, 12
y=0.5
% genes with ≥ 3 CT crRNAs:
88%, 89%, 90%, 89%, 89%, 91%
x=3
Number of CT crRNAs per gene
C
Probability
median NCT crRNAs per gene:
16, 18, 18, 18, 18, 21
y=0.5
% genes with ≥ 3 NCT crRNAs:
94%, 95%, 96%, 95%, 96%, 97%
x=3
Number of NCT crRNAs per gene
D
n = 2520
r = 0.964
C2998
C2924

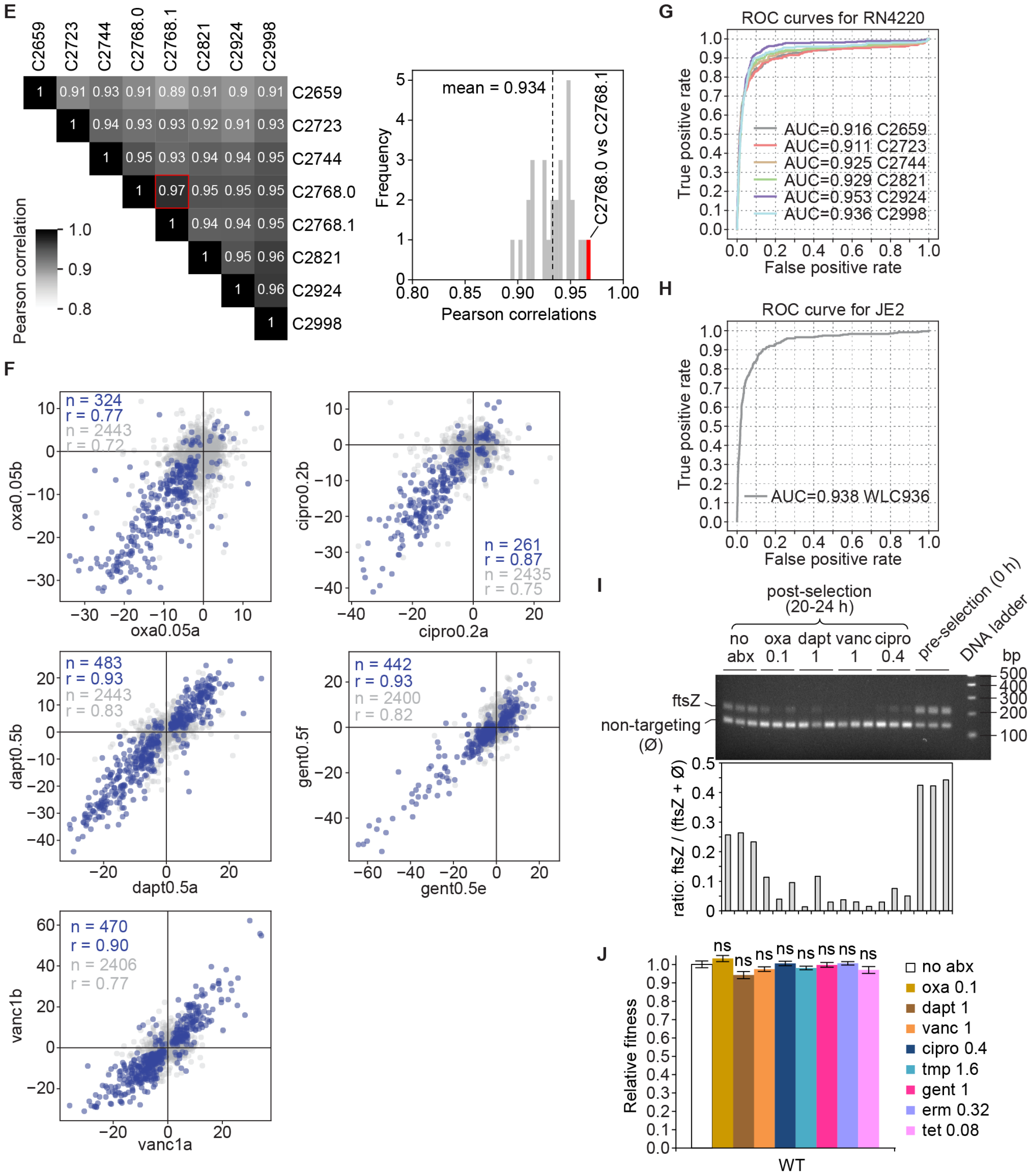

E
C2659 C2723 C2744 C2768.0 C2768.1 C2821 C2924 C2998
1 0.91 0.93 0.91 0.89 0.91 0.9 0.91 C2659
1 0.94 0.93 0.93 0.92 0.91 0.93 C2723
1 0.95 0.93 0.94 0.94 0.95 C2744
1 0.97 0.95 0.95 0.95 C2768.0
1 0.94 0.94 0.95 C2768.1
1 0.95 0.96 C2821
1 0.96 C2924
1 C2998
Pearson correlation
1.0
0.9
0.8
mean = 0.934
C2768.0 vs C2768.1
Frequency
Pearson correlations
G
ROC curves for RN4220
True positive rate
False positive rate
AUC=0.916 C2659
AUC=0.911 C2723
AUC=0.925 C2744
AUC=0.929 C2821
AUC=0.953 C2924
AUC=0.936 C2998
H
ROC curve for JE2
AUC=0.938 WLC936
F
n = 324
r = 0.77
n = 2443
r = 0.72
oxa0.05b
oxa0.05a
n = 261
r = 0.87
n = 2435
r = 0.75
cipro0.2b
cipro0.2a
n = 483
r = 0.93
n = 2443
r = 0.83
dapt0.5b
dapt0.5a
n = 442
r = 0.93
n = 2400
r = 0.82
gent0.5f
gent0.5e
n = 470
r = 0.90
n = 2406
r = 0.77
vanc1b
vanc1a
I
post-selection
(20-24 h)
no abx
oxa 0.1
dapt 1
vanc 1
cipro 0.4
pre-selection (0 h)
DNA ladder
bp
500
400
300
200
100
ftsZ
non-targeting
(Ø)
ratio: ftsZ / (ftsZ + Ø)
J
Relative fitness
ns
WT
no abx
oxa 0.1
dapt 1
vanc 1
cipro 0.4
tmp 1.6
gent 1
erm 0.32
tet 0.08

Fig. S1. Antibiotic conditions and quality controls. (A) Titration growth curves of *S. aureus* RN4220 grown in ten antibiotics used in this study. Concentrations most relevant to the CRISPRi screens are shown in warm colors. For erythromycin, tetracycline, linezolid, mupirocin, and trimethoprim, the relative exponential growth rates of cultures grown in selected antibiotic-containing media (red curves) were quantified and shown as percentages compared to those grown in plain TSB. **(B)** Cumulative distribution function plot of the number of coding-strand-targeting (CT) crRNAs per gene from six independent CALM-CRISPRi libraries made in RN4220. Libraries created by CALM-CRISPRi are highly comprehensive, with 88-91% of genes covered by 3 or more CT crRNAs, and a median CT crRNAs per gene being 10-12 from these replicates. **(C)** Same as (B) but showing the noncoding-strand-targeting (NCT) crRNAs. **(D)** Pearson correlation between the fitness scores of RN4220 genes ($Z_{Ø}$) from two independent CALM-CRISPRi libraries grown in TSB for 9 hours. Only genes targeted by at least 2 CT crRNAs were included in analysis. **(E)** Left: Pairwise Pearson correlations of the fitness scores of RN4220 genes ($Z_{Ø}$) from all seven independent CALM-CRISPRi libraries grown in TSB for 9 hours. Libraries C2768.0 and C2768.1 were made in the same experiment, serving as a batch control (red box) for other libraries generated in separate experiments. Right: Distribution of all Pairwise Pearson correlations on the left panel. Red bar indicates correlation between C2768.0 and C2768.1. **(F)** Pairwise Pearson correlations between the relative fitness scores of RN4220 genes in antibiotics (Z_{abx}) from two independent CALM-CRISPRi libraries. The numbers of genes passing quality control and their correlations are shown in gray in upper left or lower right corners. The numbers of genes with significantly altered fitness in antibiotics relative to TSB ($|Z_{abx}| \geq 9$, $P_{adj} < 0.05$) and their correlations are shown in blue. **(G)** ROC curves of six independent CALM-CRISPRi libraries generated in *S. aureus* RN4220 and grown in TSB for 9 hours. Gene essentiality determined by Santiago's Tn-seq study (*22*) was used as a reference. **(H)** ROC curve of CALM-CRISPRi library generated in *S. aureus* JE2 and grown in TSB for 9 hours. Gene essentiality determined by Coe's Tn-seq study (*4*) was used as a reference. **(I)** An example of the quantification of pairwise competition assays in triplicates. Equal amount of RN4220 with a spacer targeting *ftsZ*, and RN4220 with a non-targeting spacer (ie, the common competitor, Ø) were mixed, representing the pre-selected samples at 0 h. Mixed bacteria were inoculated into TSB and TSB containing various antibiotics (concentration shown as µg/mL) and grown for 20 - 24 hours at 37 °C. DNA from pre- and post-selected samples were used as templates for PCR amplifying the CRISPR spacer region and visualized on agarose gel (upper panel). The ratios of normalized intensity of *ftsZ* to total (*ftsZ* + Ø) were calculated and shown as bar plots (lower panel). **(J)** Pairwise competition assays (Methods) measuring the relative fitness of *S. aureus* RN4220 carrying an IPTG-inducible CRISPRi system that does not have a genomic target (ie, WT) in indicated antibiotic conditions. Essentially, two WT strains were being competed. Concentrations of antibiotics are shown in µg/mL. Error bars indicate the standard deviation from three biological replicates. For each antibiotic, a paired t-test was performed comparing the antibiotic-treated condition to the no-antibiotic control.

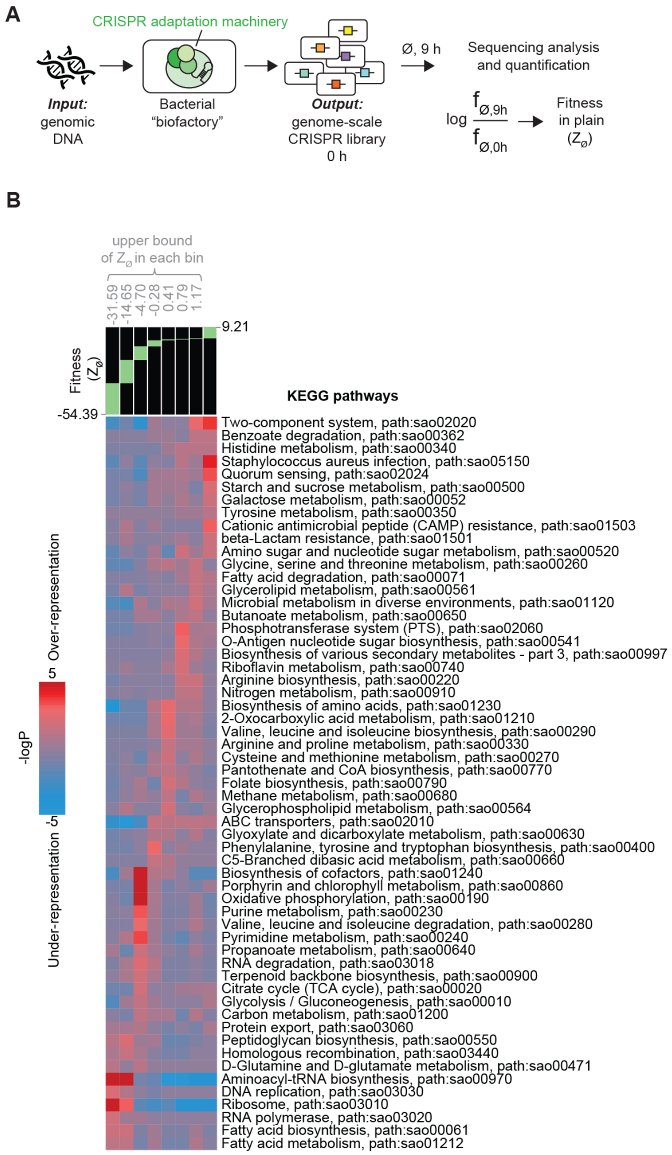

A
CRISPR adaptation machinery
Input:
genomic DNA
Bacterial "biofactory"
Output:
genome-scale CRISPR library
0 h
Ø, 9 h
Sequencing analysis and quantification
log f Ø,9h / f Ø,0h
Fitness in plain (ZØ)
B
upper bound of ZØ in each bin
-31.59
-14.65
-4.70
-0.28
0.41
0.79
1.17
9.21
-54.39
Fitness (ZØ)
KEGG pathways
-logP
5
-5
Over-representation
Under-representation
Two-component system, path:sao02020
Benzoate degradation, path:sao00362
Histidine metabolism, path:sao00340
Staphylococcus aureus infection, path:sao05150
Quorum sensing, path:sao02024
Starch and sucrose metabolism, path:sao00500
Galactose metabolism, path:sao00052
Tyrosine metabolism, path:sao00350
Cationic antimicrobial peptide (CAMP) resistance, path:sao01503
beta-Lactam resistance, path:sao01501
Amino sugar and nucleotide sugar metabolism, path:sao00520
Glycine, serine and threonine metabolism, path:sao00260
Fatty acid degradation, path:sao00071
Glycerolipid metabolism, path:sao00561
Microbial metabolism in diverse environments, path:sao01120
Butanoate metabolism, path:sao00650
Phosphotransferase system (PTS), path:sao02060
O-Antigen nucleotide sugar biosynthesis, path:sao00541
Biosynthesis of various secondary metabolites - part 3, path:sao00997
Riboflavin metabolism, path:sao00740
Arginine biosynthesis, path:sao00220
Nitrogen metabolism, path:sao00910
Biosynthesis of amino acids, path:sao01230
2-Oxocarboxylic acid metabolism, path:sao01210
Valine, leucine and isoleucine biosynthesis, path:sao00290
Arginine and proline metabolism, path:sao00330
Cysteine and methionine metabolism, path:sao00270
Pantothenate and CoA biosynthesis, path:sao00770
Folate biosynthesis, path:sao00790
Methane metabolism, path:sao00680
Glycerophospholipid metabolism, path:sao00564
ABC transporters, path:sao02010
Glyoxylate and dicarboxylate metabolism, path:sao00630
Phenylalanine, tyrosine and tryptophan biosynthesis, path:sao00400
C5-Branched dibasic acid metabolism, path:sao00660
Biosynthesis of cofactors, path:sao01240
Porphyrin and chlorophyll metabolism, path:sao00860
Oxidative phosphorylation, path:sao00190
Purine metabolism, path:sao00230
Valine, leucine and isoleucine degradation, path:sao00280
Pyrimidine metabolism, path:sao00240
Propanoate metabolism, path:sao00640
RNA degradation, path:sao03018
Terpenoid backbone biosynthesis, path:sao00900
Citrate cycle (TCA cycle), path:sao00020
Glycolysis / Gluconeogenesis, path:sao00010
Carbon metabolism, path:sao01200
Protein export, path:sao03060
Peptidoglycan biosynthesis, path:sao00550
Homologous recombination, path:sao03440
D-Glutamine and D-glutamate metabolism, path:sao00471
Aminoacyl-tRNA biosynthesis, path:sao00970
DNA replication, path:sao03030
Ribosome, path:sao03010
RNA polymerase, path:sao03020
Fatty acid biosynthesis, path:sao00061
Fatty acid metabolism, path:sao01212

C

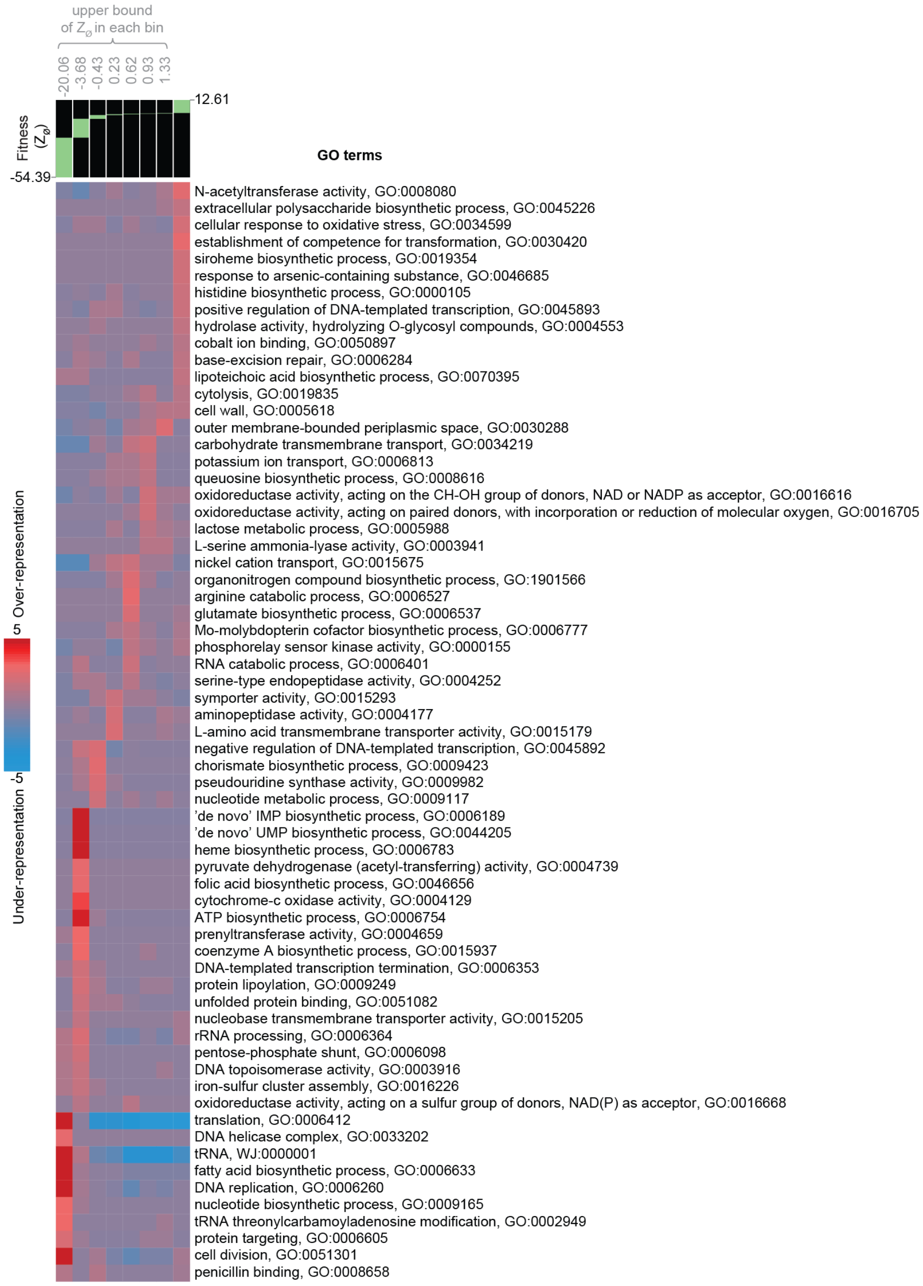

Fig. S2. Functional enrichment analysis of the gene essentiality landscape in *S. aureus* RN4220. (A) CALM-CRISPRi libraries of *S. aureus* RN4220 were grown in plain media, TSB, for 9 hours. Gene fitness in TSB ($Z_{\varnothing}$) was quantified by sequencing analysis (Methods). For each gene, the mean $Z_{\varnothing}$ was calculated from three biological replicates. **(B)** KEGG pathway enrichment analysis was performed using the mean $Z_{\varnothing}$ values of genes and the iPAGE tool (*79*). $Z_{\varnothing}$ of genes was ranked and binned. For each bin, the top black-green panel shows the lower and upper bounds of $Z_{\varnothing}$, and the bottom red-blue panel shows the degree of overrepresentation (red) and underrepresentation (blue) of genes belonging to the KEGG pathway. **(C)** Same as (B) except iPAGE enrichment analysis was done on GO terms.

A

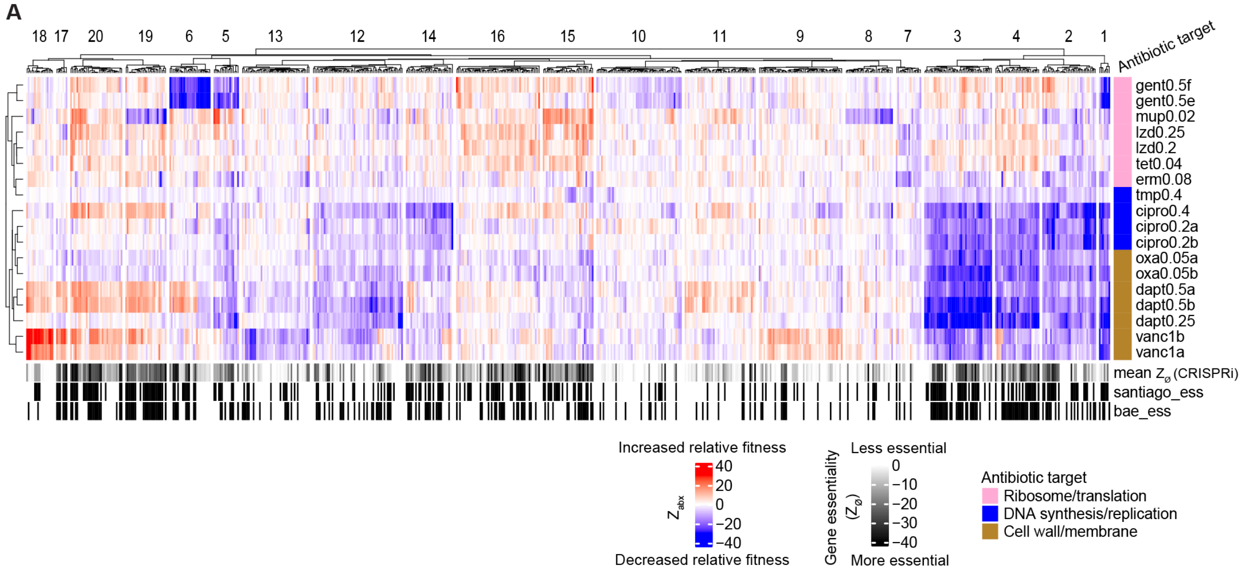

C

Antibiotic condition	Sensitizing abx-gene interactions	Desensitizing abx-gene interactions
dapt0.25	222	4
dapt0.5a	157	104
dapt0.5b	217	112
oxa0.05a	186	5
oxa0.05b	225	3
vanc1a	146	80
vanc1b	186	124
cipro0.2a	186	12
cipro0.2b	173	8
cipro0.4	229	91
tmp0.4	59	4
gent0.5e	86	36
gent0.5f	69	76
erm0.08	99	20
lzd0.2	18	37
lzd0.25	32	95
mup0.02	86	91
tet0.04	33	75

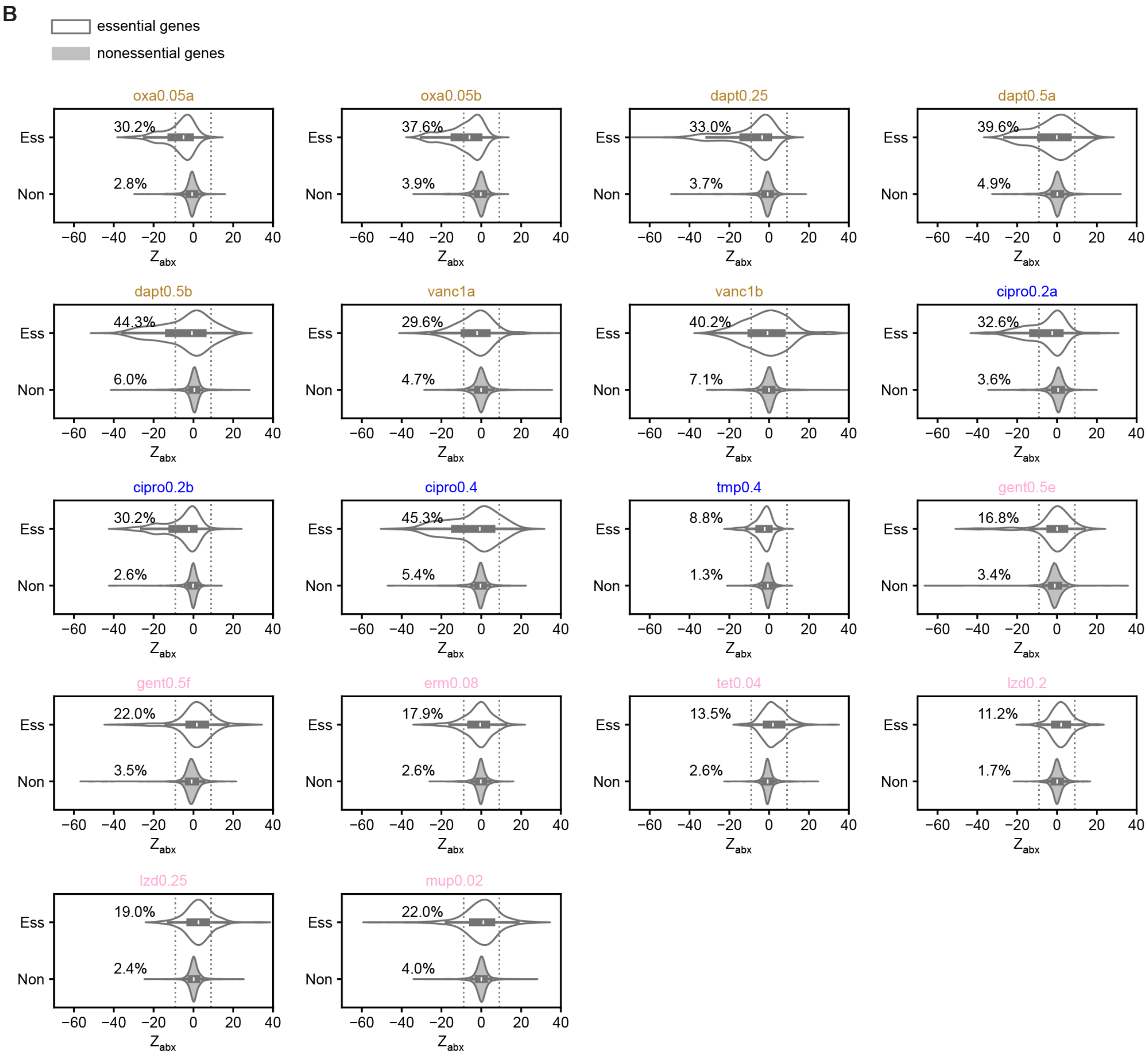

Fig. S3. Antibiotic-gene interactions in *S. aureus* RN4220. (A) Hierarchical clustering of 650 genes whose repression by CALM-CRISPRi significantly increased (red) or decreased (blue) relative fitness in at least one antibiotic condition ($|Z_{abx}| \geq 9$ and $P_{adj} < 0.05$). A total of 18 antibiotic conditions (including biological replicates) is shown. For each gene, its essentiality in plain TSB (black and white) was quantified as $Z_{Ø}$ by CRISPRi (mean of triplicates) in this study, and qualified (binary) by Santiago's (*22*) and Bae's (*24*) Tn-seq studies. Antibiotics are color coded by their targets. $Z_{Ø}$, Z_{abx}, and clustering are shown in Tables S2, S3, and S5, respectively. **(B)** Distributions of Z_{abx} for 382 essential genes identified by Santiago's Tn-seq (*22*) and 2,138 nonessential genes in 18 antibiotic conditions. In each panel, dotted lines indicate $|Z_{abx}| = 9$, and percentages of essential (ess) and nonessential (non) genes with $|Z_{abx}| \geq 9$ are indicated. **(C)** Numbers of sensitizing and desensitizing antibiotic-gene interactions for the 18 antibiotic conditions shown in Fig. 1F. Antibiotic conditions with replicates are labeled with letters.

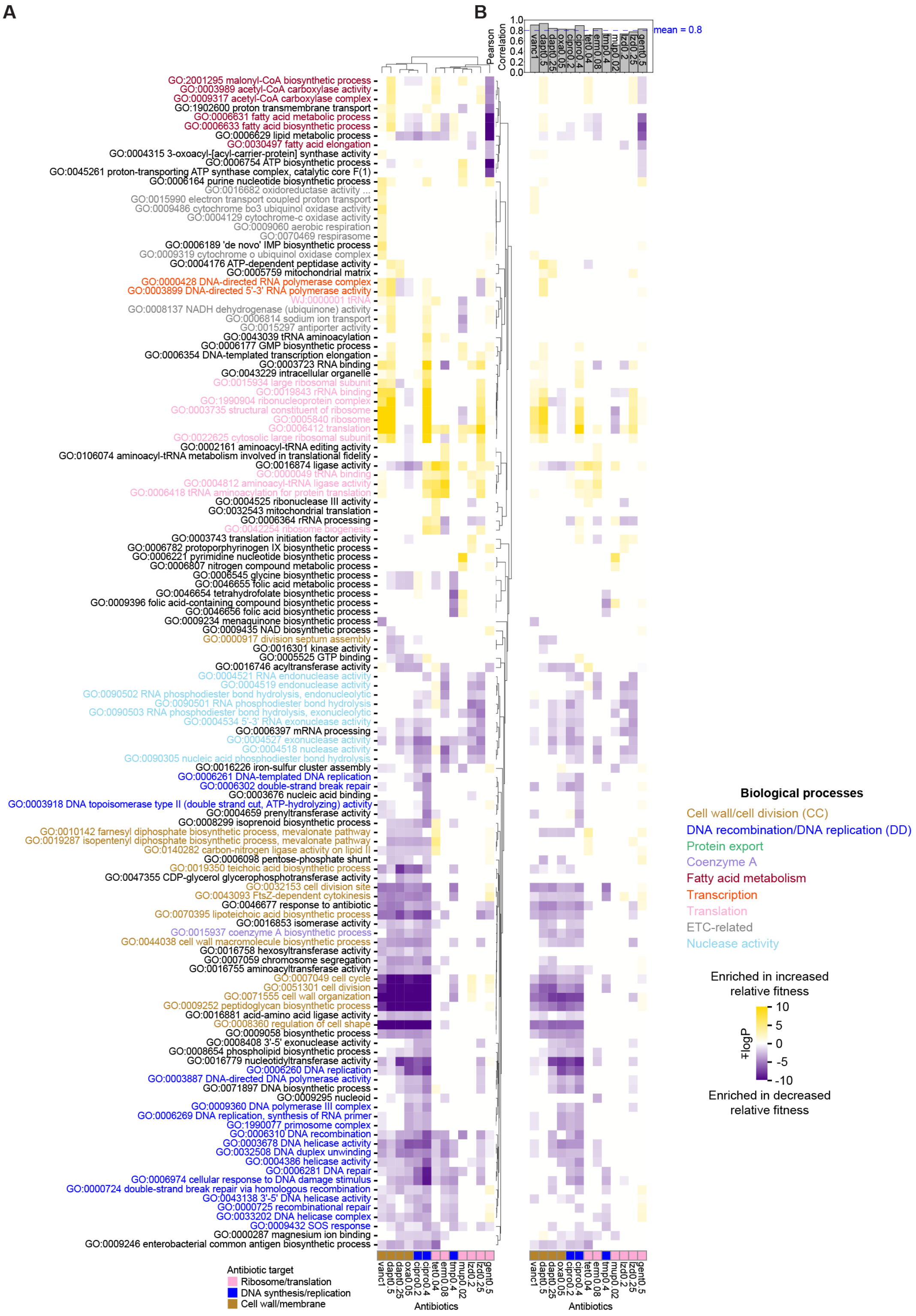

A
B
Pearson Correlation
1.0
0.8
0.6
0.4
0.2
0.0
mean = 0.8
vanc1
dapt0.5
dapt0.25
oxa0.05
cipro0.2
cipro0.4
tet0.04
erm0.08
tmp0.4
mup0.02
lzd0.2
lzd0.25
gent0.5
GO:2001295 malonyl-CoA biosynthetic process
GO:0003989 acetyl-CoA carboxylase activity
GO:0009317 acetyl-CoA carboxylase complex
GO:1902600 proton transmembrane transport
GO:0006631 fatty acid metabolic process
GO:0006633 fatty acid biosynthetic process
GO:0006629 lipid metabolic process
GO:0030497 fatty acid elongation
GO:0004315 3-oxoacyl-[acyl-carrier-protein] synthase activity
GO:0006754 ATP biosynthetic process
GO:0045261 proton-transporting ATP synthase complex, catalytic core F(1)
GO:0006164 purine nucleotide biosynthetic process
GO:0016682 oxidoreductase activity ...
GO:0015990 electron transport coupled proton transport
GO:0009486 cytochrome bo3 ubiquinol oxidase activity
GO:0004129 cytochrome-c oxidase activity
GO:0009060 aerobic respiration
GO:0070469 respirasome
GO:0006189 'de novo' IMP biosynthetic process
GO:0009319 cytochrome o ubiquinol oxidase complex
GO:0004176 ATP-dependent peptidase activity
GO:0005759 mitochondrial matrix
GO:0000428 DNA-directed RNA polymerase complex
GO:0003899 DNA-directed 5'-3' RNA polymerase activity
WJ:0000001 tRNA
GO:0008137 NADH dehydrogenase (ubiquinone) activity
GO:0006814 sodium ion transport
GO:0015297 antiporter activity
GO:0043039 tRNA aminoacylation
GO:0006177 GMP biosynthetic process
GO:0006354 DNA-templated transcription elongation
GO:0003723 RNA binding
GO:0043229 intracellular organelle
GO:0015934 large ribosomal subunit
GO:0019843 rRNA binding
GO:1990904 ribonucleoprotein complex
GO:0003735 structural constituent of ribosome
GO:0005840 ribosome
GO:0006412 translation
GO:0022625 cytosolic large ribosomal subunit
GO:0002161 aminoacyl-tRNA editing activity
GO:0106074 aminoacyl-tRNA metabolism involved in translational fidelity
GO:0016874 ligase activity
GO:0000049 tRNA binding
GO:0004812 aminoacyl-tRNA ligase activity
GO:0006418 tRNA aminoacylation for protein translation
GO:0004525 ribonuclease III activity
GO:0032543 mitochondrial translation
GO:0006364 rRNA processing
GO:0042254 ribosome biogenesis
GO:0003743 translation initiation factor activity
GO:0006782 protoporphyrinogen IX biosynthetic process
GO:0006221 pyrimidine nucleotide biosynthetic process
GO:0006807 nitrogen compound metabolic process
GO:0006545 glycine biosynthetic process
GO:0046655 folic acid metabolic process
GO:0046654 tetrahydrofolate biosynthetic process
GO:0009396 folic acid-containing compound biosynthetic process
GO:0046656 folic acid biosynthetic process
GO:0009234 menaquinone biosynthetic process
GO:0009435 NAD biosynthetic process
GO:0000917 division septum assembly
GO:0016301 kinase activity
GO:0005525 GTP binding
GO:0016746 acyltransferase activity
GO:0004521 RNA endonuclease activity
GO:0004519 endonuclease activity
GO:0090502 RNA phosphodiester bond hydrolysis, endonucleolytic
GO:0090501 RNA phosphodiester bond hydrolysis
GO:0090503 RNA phosphodiester bond hydrolysis, exonucleolytic
GO:0004534 5'-3' RNA exonuclease activity
GO:0006397 mRNA processing
GO:0004527 exonuclease activity
GO:0004518 nuclease activity
GO:0090305 nucleic acid phosphodiester bond hydrolysis
GO:0016226 iron-sulfur cluster assembly
GO:0006261 DNA-templated DNA replication
GO:0006302 double-strand break repair
GO:0003676 nucleic acid binding
GO:0003918 DNA topoisomerase type II (double strand cut, ATP-hydrolyzing) activity
GO:0004659 prenyltransferase activity
GO:0008299 isoprenoid biosynthetic process
GO:0010142 farnesyl diphosphate biosynthetic process, mevalonate pathway
GO:0019287 isopentenyl diphosphate biosynthetic process, mevalonate pathway
GO:0140282 carbon-nitrogen ligase activity on lipid II
GO:0006098 pentose-phosphate shunt
GO:0019350 teichoic acid biosynthetic process
GO:0047355 CDP-glycerol glycerophosphotransferase activity
GO:0032153 cell division site
GO:0043093 FtsZ-dependent cytokinesis
GO:0046677 response to antibiotic
GO:0070395 lipoteichoic acid biosynthetic process
GO:0016853 isomerase activity
GO:0015937 coenzyme A biosynthetic process
GO:0044038 cell wall macromolecule biosynthetic process
GO:0016758 hexosyltransferase activity
GO:0007059 chromosome segregation
GO:0016755 aminoacyltransferase activity
GO:0007049 cell cycle
GO:0051301 cell division
GO:0071555 cell wall organization
GO:0009252 peptidoglycan biosynthetic process
GO:0016881 acid-amino acid ligase activity
GO:0008360 regulation of cell shape
GO:0009058 biosynthetic process
GO:0008408 3'-5' exonuclease activity
GO:0008654 phospholipid biosynthetic process
GO:0016779 nucleotidyltransferase activity
GO:0006260 DNA replication
GO:0003887 DNA-directed DNA polymerase activity
GO:0071897 DNA biosynthetic process
GO:0009295 nucleoid
GO:0009360 DNA polymerase III complex
GO:0006269 DNA replication, synthesis of RNA primer
GO:1990077 primosome complex
GO:0006310 DNA recombination
GO:0003678 DNA helicase activity
GO:0032508 DNA duplex unwinding
GO:0004386 helicase activity
GO:0006281 DNA repair
GO:0006974 cellular response to DNA damage stimulus
GO:0000724 double-strand break repair via homologous recombination
GO:0043138 3'-5' DNA helicase activity
GO:0000725 recombinational repair
GO:0033202 DNA helicase complex
GO:0009432 SOS response
GO:0000287 magnesium ion binding
GO:0009246 enterobacterial common antigen biosynthetic process
Antibiotic target
Ribosome/translation
DNA synthesis/replication
Cell wall/membrane
Antibiotics
Biological processes
Cell wall/cell division (CC)
DNA recombination/DNA replication (DD)
Protein export
Coenzyme A
Fatty acid metabolism
Transcription
Translation
ETC-related
Nuclease activity
Enriched in increased relative fitness
±logP
10
5
0
-5
-10
Enriched in decreased relative fitness

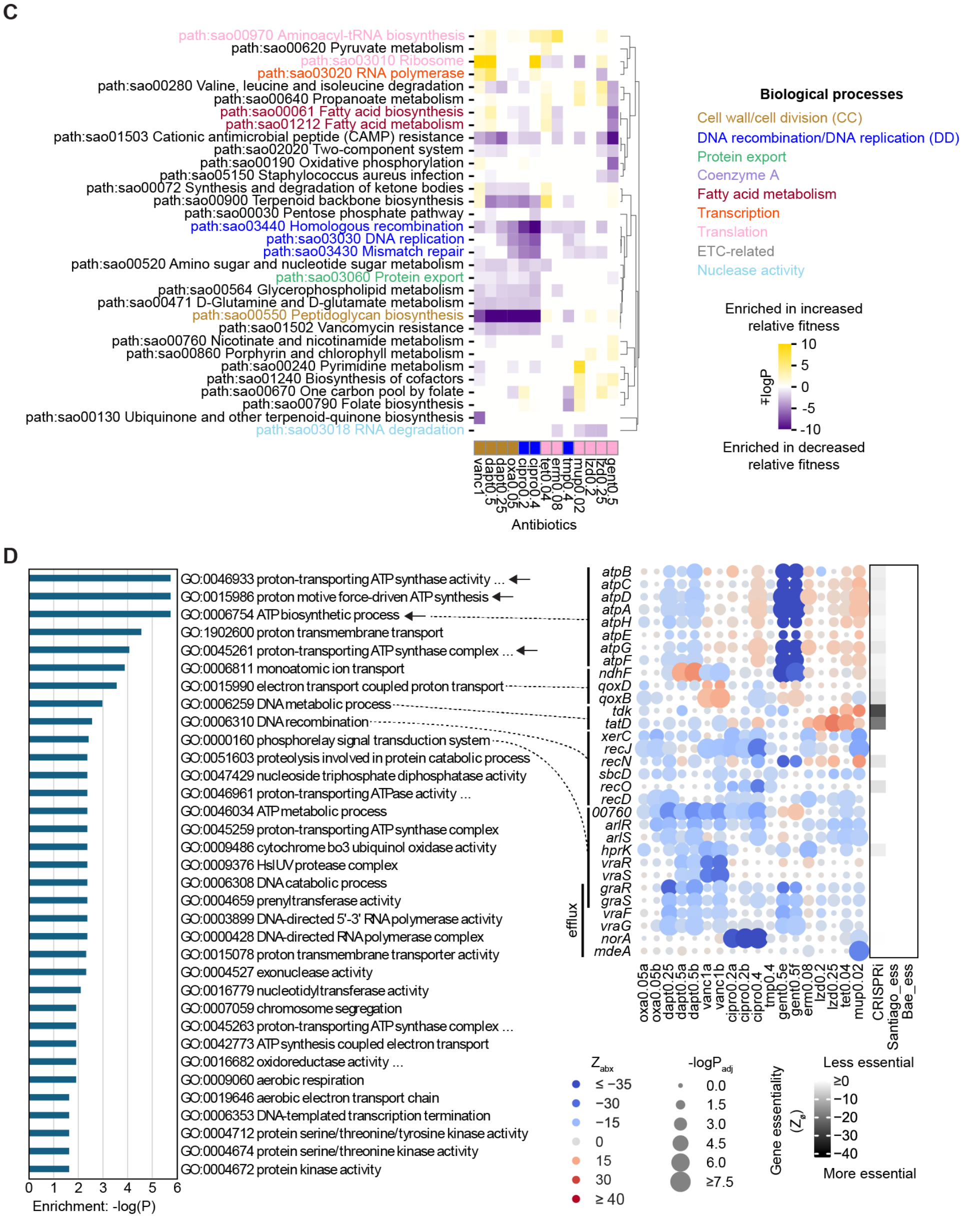

C
path:sao00970 Aminoacyl-tRNA biosynthesis
path:sao00620 Pyruvate metabolism
path:sao03010 Ribosome
path:sao03020 RNA polymerase
path:sao00280 Valine, leucine and isoleucine degradation
path:sao00640 Propanoate metabolism
path:sao00061 Fatty acid biosynthesis
path:sao01212 Fatty acid metabolism
path:sao01503 Cationic antimicrobial peptide (CAMP) resistance
path:sao02020 Two-component system
path:sao00190 Oxidative phosphorylation
path:sao05150 Staphylococcus aureus infection
path:sao00072 Synthesis and degradation of ketone bodies
path:sao00900 Terpenoid backbone biosynthesis
path:sao00030 Pentose phosphate pathway
path:sao03440 Homologous recombination
path:sao03030 DNA replication
path:sao03430 Mismatch repair
path:sao00520 Amino sugar and nucleotide sugar metabolism
path:sao03060 Protein export
path:sao00564 Glycerophospholipid metabolism
path:sao00471 D-Glutamine and D-glutamate metabolism
path:sao00550 Peptidoglycan biosynthesis
path:sao01502 Vancomycin resistance
path:sao00760 Nicotinate and nicotinamide metabolism
path:sao00860 Porphyrin and chlorophyll metabolism
path:sao00240 Pyrimidine metabolism
path:sao01240 Biosynthesis of cofactors
path:sao00670 One carbon pool by folate
path:sao00790 Folate biosynthesis
path:sao00130 Ubiquinone and other terpenoid-quinone biosynthesis
path:sao03018 RNA degradation
vanc1
dapt0.5
dapt0.25
oxa0.05
cipro0.2
cipro0.4
tet0.04
erm0.08
tmp0.4
mup0.02
lzd0.2
lzd0.25
gent0.5
Antibiotics
Biological processes
Cell wall/cell division (CC)
DNA recombination/DNA replication (DD)
Protein export
Coenzyme A
Fatty acid metabolism
Transcription
Translation
ETC-related
Nuclease activity
Enriched in increased relative fitness
10
5
0
-5
-10
∓logP
Enriched in decreased relative fitness
D
GO:0046933 proton-transporting ATP synthase activity ...
GO:0015986 proton motive force-driven ATP synthesis
GO:0006754 ATP biosynthetic process
GO:1902600 proton transmembrane transport
GO:0045261 proton-transporting ATP synthase complex ...
GO:0006811 monoatomic ion transport
GO:0015990 electron transport coupled proton transport
GO:0006259 DNA metabolic process
GO:0006310 DNA recombination
GO:0000160 phosphorelay signal transduction system
GO:0051603 proteolysis involved in protein catabolic process
GO:0047429 nucleoside triphosphate diphosphatase activity
GO:0046961 proton-transporting ATPase activity ...
GO:0046034 ATP metabolic process
GO:0045259 proton-transporting ATP synthase complex
GO:0009486 cytochrome bo3 ubiquinol oxidase activity
GO:0009376 HslUV protease complex
GO:0006308 DNA catabolic process
GO:0004659 prenyltransferase activity
GO:0003899 DNA-directed 5'-3' RNA polymerase activity
GO:0000428 DNA-directed RNA polymerase complex
GO:0015078 proton transmembrane transporter activity
GO:0004527 exonuclease activity
GO:0016779 nucleotidyltransferase activity
GO:0007059 chromosome segregation
GO:0045263 proton-transporting ATP synthase complex ...
GO:0042773 ATP synthesis coupled electron transport
GO:0016682 oxidoreductase activity ...
GO:0009060 aerobic respiration
GO:0019646 aerobic electron transport chain
GO:0006353 DNA-templated transcription termination
GO:0004712 protein serine/threonine/tyrosine kinase activity
GO:0004674 protein serine/threonine kinase activity
GO:0004672 protein kinase activity
0 1 2 3 4 5 6
Enrichment: -log(P)
atpB
atpC
atpD
atpA
atpH
atpE
atpG
atpF
ndhF
qoxD
qoxB
tdk
tatD
xerC
recJ
recN
sbcD
recO
recD
00760
arlR
arlS
hprK
vraR
vraS
graR
graS
vraF
vraG
norA
mdeA
efflux
oxa0.05a
oxa0.05b
dapt0.25
dapt0.5a
dapt0.5b
vanc1a
vanc1b
cipro0.2a
cipro0.2b
cipro0.4
tmp0.4
gent0.5e
gent0.5f
erm0.08
lzd0.2
lzd0.25
tet0.04
mup0.02
CRISPRi
Santiago_ess
Bae_ess
Zabx
≤ −35
−30
−15
0
15
30
≥ 40
-logPadj
0.0
1.5
3.0
4.5
6.0
≥7.5
Gene essentiality (Ze)
Less essential
≥0
−10
−20
−30
−40
More essential

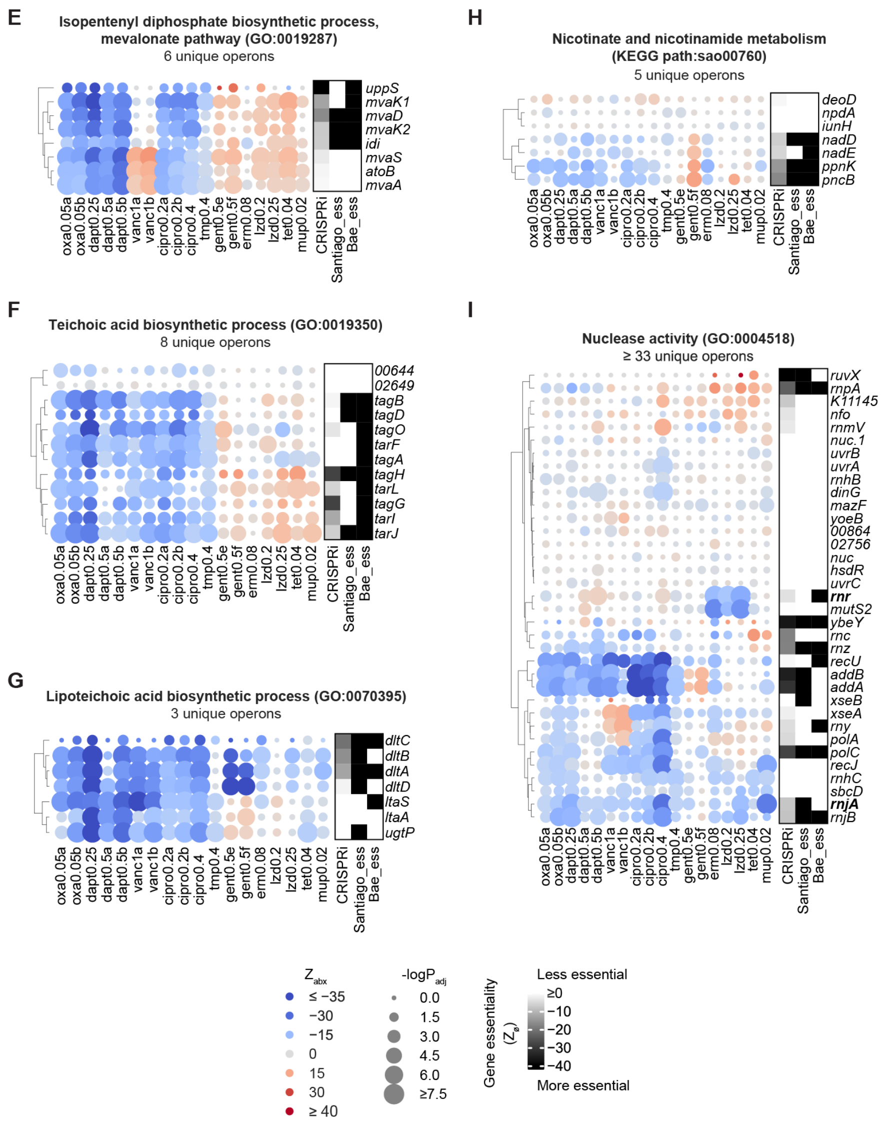
E
Isopentenyl diphosphate biosynthetic process, mevalonate pathway (GO:0019287)
6 unique operons
uppS
mvaK1
mvaD
mvaK2
idi
mvaS
atoB
mvaA
H
Nicotinate and nicotinamide metabolism (KEGG path:sao00760)
5 unique operons
deoD
npdA
iunH
nadD
nadE
ppnK
pncB
F
Teichoic acid biosynthetic process (GO:0019350)
8 unique operons
00644
02649
tagB
tagD
tagO
tarF
tagA
tagH
tarL
tagG
tarI
tarJ
I
Nuclease activity (GO:0004518)
≥ 33 unique operons
ruvX
rnpA
K11145
nfo
rnmV
nuc.1
uvrB
uvrA
rnhB
dinG
mazF
yoeB
00864
02756
nuc
hsdR
uvrC
rnr
mutS2
ybeY
rnc
rnz
recU
addB
addA
xseB
xseA
rny
polA
polC
recJ
rnhC
sbcD
rnjA
rnjB
G
Lipoteichoic acid biosynthetic process (GO:0070395)
3 unique operons
dltC
dltB
dltA
dltD
ltaS
ltaA
ugtP
oxa0.05a
oxa0.05b
dapt0.25
dapt0.5a
dapt0.5b
vanc1a
vanc1b
cipro0.2a
cipro0.2b
cipro0.4
tmp0.4
gent0.5e
gent0.5f
erm0.08
lzd0.2
lzd0.25
tet0.04
mup0.02
CRISPRi
Santiago_ess
Bae_ess
Z abx
≤ −35
−30
−15
0
15
30
≥ 40
-logP adj
0.0
1.5
3.0
4.5
6.0
≥7.5
Gene essentiality (Z e)
Less essential
≥0
−10
−20
−30
−40
More essential

Fig. S4. Biological processes that modulate antibiotic sensitivity in *S. aureus* RN4220. (A) Functional enrichment analysis by iPAGE (*79*) followed by hierarchical clustering of all significantly enriched GO terms. The ∓log(P-value) of GO terms significantly enriched ($P < 0.001$) for genes whose repression increased and decreased relative fitness ($|Z_{abx}| \geq 9$ and $P_{adj} < 0.05$) are shown in yellow and purple, respectively. Color code for biological processes is the same as Fig. 1G. We added a new term called "WJ:0000001 tRNA" as tRNA genes are annotated in neither GO terms nor KEGG pathways. Clustering of all GO terms is shown in Table S6. **(B)** Bottom: Same as (A) except that enrichment analysis was performed using only the last gene in each of the 1691 operons annotated by BioCyc (http://biocyc.org/). Rows and columns are in the same order as those in (A). Clustering of all GO terms is shown in Table S7. Top: Pearson correlation coefficient for each antibiotic condition between enrichment analyses performed using full gene set and the last gene in each operon. **(C)** Same as (A) except showing clustering of all significantly enriched KEGG pathways. Antibiotic columns are in the same order as those in (A). Clustering of all KEGG pathways is shown in Table S8. **(D)** Biological processes that were enriched for nonessential genes whose repression significantly altered relative fitness in antibiotics. Left: name of biological processes and their level of enrichment, shown as -log(P), among all nonessential genes that significantly altered relative fitness in antibiotic conditions. Right: blue-red heatmap showing the relative fitness of genes from select Gene Ontology terms in ten antibiotics as quantified by CALM-CRISPRi screens (Z_{abx}). Antibiotic conditions with replicates are labeled with letters. Gene names are annotated by KEGG orthology (ko). Gene knockdowns that decreased and increased relative fitness in antibiotics are shown in blue and red circles, respectively. Size of circle indicates the negative of log-transformed adjusted P-value, calculated by a Mann-Whitney U test between gene-specific crRNAs and null crRNAs (Methods). Gene essentiality quantified by CRISPRi (mean of $Z_{\emptyset}$ from triplicates) and qualified (binary) by Santiago's (*22*) and Bae's (*24*) Tn-seq studies are shown in black-white heatmap. **(E)** Same as the right panel of (D) but showing genes in the isopentenyl diphosphate biosynthetic process, mevalonate pathway (GO:0019287). *atoB*, *mvaS*, *mvaA*, *idi* and *uppS* were also added to the heatmap due to overannotation by GO terms. **(F)** Same as the right panel of (D) but showing genes in teichoic acid biosynthetic process (GO:0019350). *tagGHO* were also added to the heatmap due to overannotation by GO terms. **(G to I)** Same as the right panel of (D) but showing genes in other biological processes.

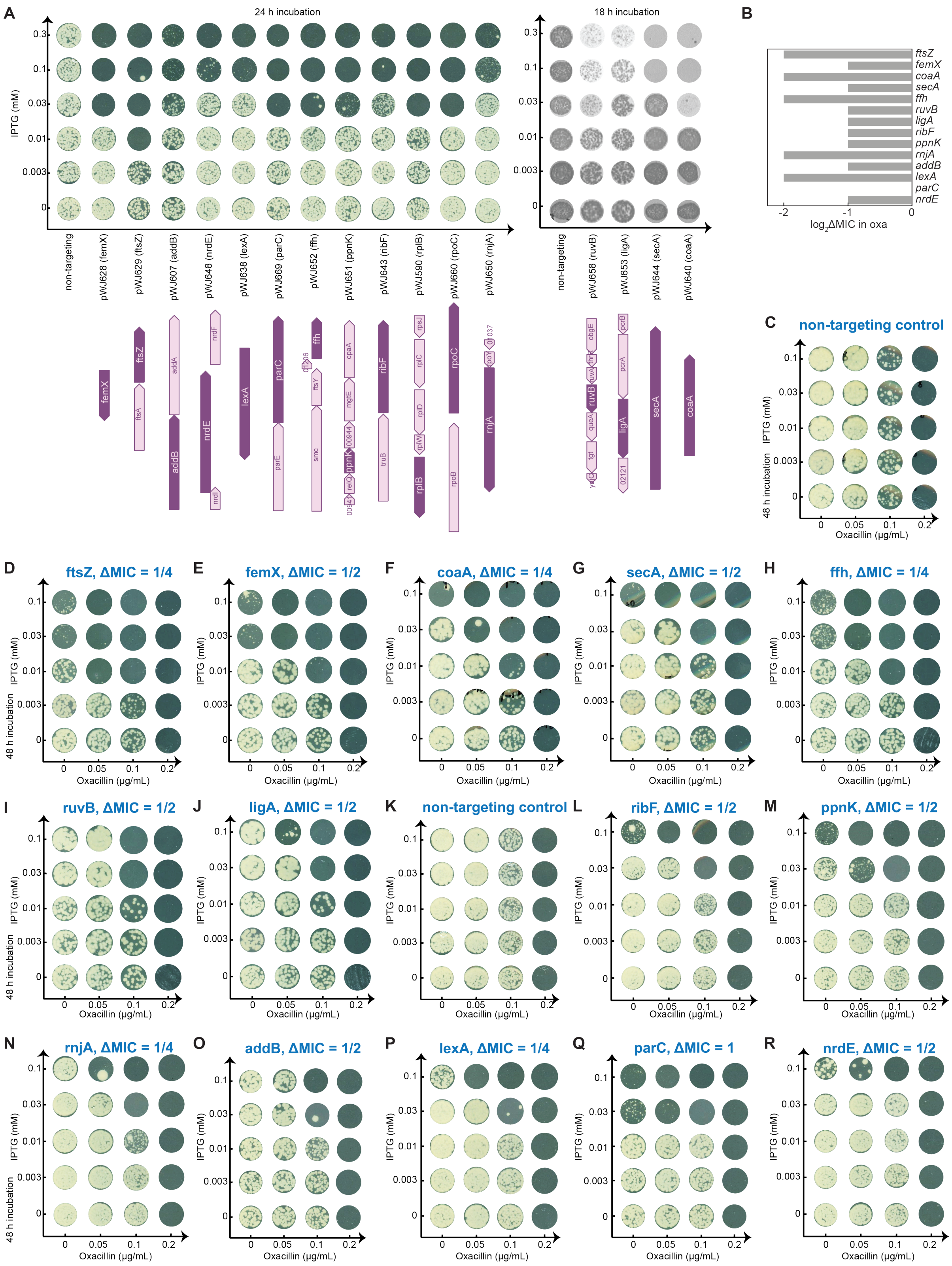

A
24 h incubation
18 h incubation
IPTG (mM)
non-targeting
pWJ628 (femX)
pWJ629 (ftsZ)
pWJ607 (addB)
pWJ648 (nrdE)
pWJ638 (lexA)
pWJ669 (parC)
pWJ652 (ffh)
pWJ651 (ppnK)
pWJ643 (ribF)
pWJ590 (rplB)
pWJ660 (rpoC)
pWJ650 (rnjA)
pWJ658 (ruvB)
pWJ653 (ligA)
pWJ644 (secA)
pWJ640 (coaA)
B
ftsZ
femX
coaA
secA
ffh
ruvB
ligA
ribF
ppnK
rnjA
addB
lexA
parC
nrdE
log2ΔMIC in oxa
C
non-targeting control
48 h incubation
Oxacillin (µg/mL)
D
ftsZ, ΔMIC = 1/4
E
femX, ΔMIC = 1/2
F
coaA, ΔMIC = 1/4
G
secA, ΔMIC = 1/2
H
ffh, ΔMIC = 1/4
I
ruvB, ΔMIC = 1/2
J
ligA, ΔMIC = 1/2
K
non-targeting control
L
ribF, ΔMIC = 1/2
M
ppnK, ΔMIC = 1/2
N
rnjA, ΔMIC = 1/4
O
addB, ΔMIC = 1/2
P
lexA, ΔMIC = 1/4
Q
parC, ΔMIC = 1
R
nrdE, ΔMIC = 1/2

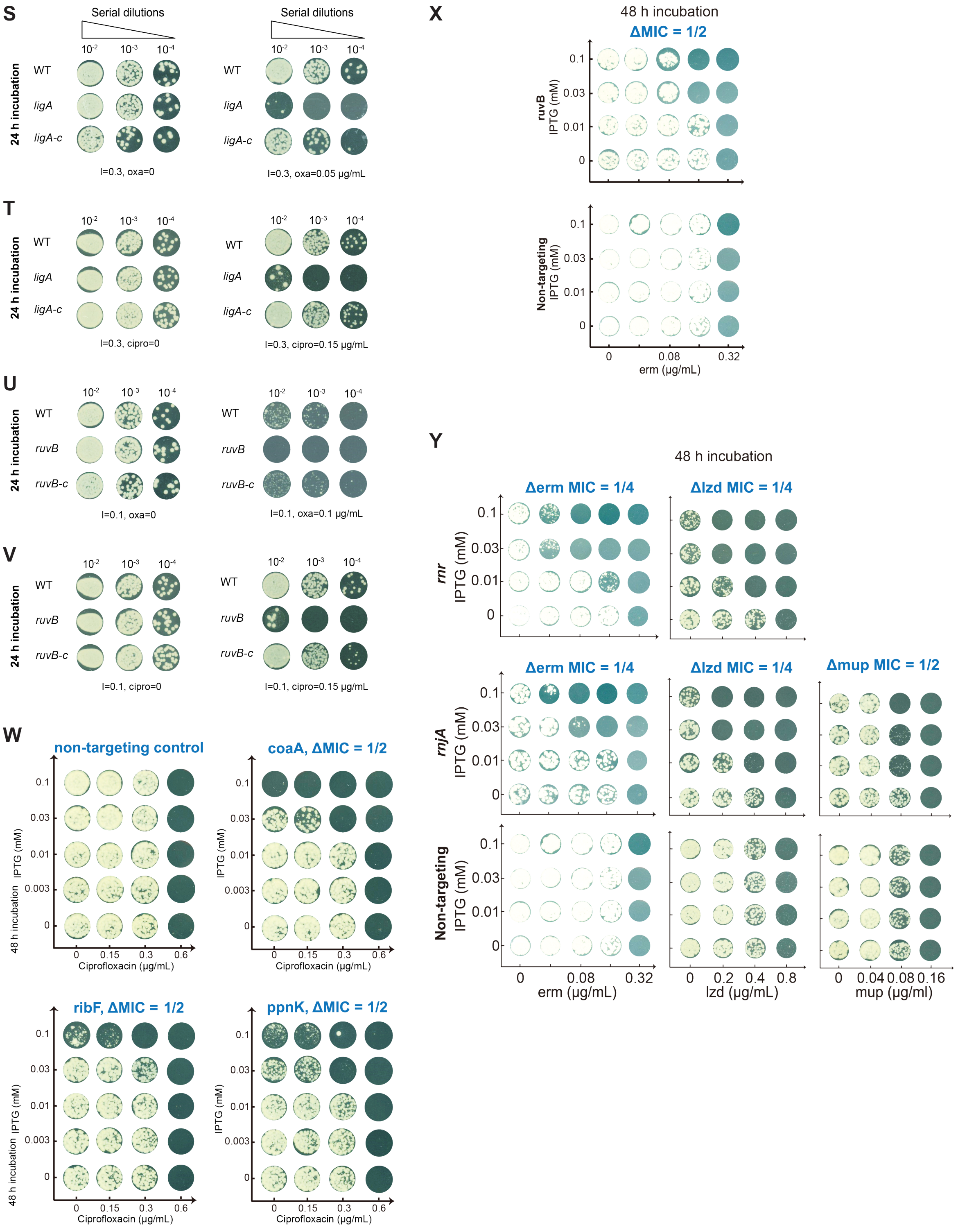
S
Serial dilutions
10-2 10-3 10-4
24 h incubation
WT
ligA
ligA-c
I=0.3, oxa=0
I=0.3, oxa=0.05 μg/mL
T
I=0.3, cipro=0
I=0.3, cipro=0.15 μg/mL
U
ruvB
ruvB-c
I=0.1, oxa=0
I=0.1, oxa=0.1 μg/mL
V
I=0.1, cipro=0
I=0.1, cipro=0.15 μg/mL
W
non-targeting control
coaA, ΔMIC = 1/2
ribF, ΔMIC = 1/2
ppnK, ΔMIC = 1/2
48 h incubation
IPTG (mM)
0.1
0.03
0.01
0.003
0
0 0.15 0.3 0.6
Ciprofloxacin (μg/mL)
X
48 h incubation
ΔMIC = 1/2
ruvB
Non-targeting
0 0.08 0.32
erm (μg/mL)
Y
48 h incubation
Δerm MIC = 1/4
Δlzd MIC = 1/4
Δmup MIC = 1/2
rnr
rnjA
0 0.2 0.4 0.8
lzd (μg/mL)
0 0.04 0.08 0.16
mup (μg/ml)

Fig. S5. Gene essentiality and checkerboard MIC assays on solid agar. (A) *S. aureus* RN4220 carrying an IPTG-inducible CRISPRi system targeting various essential genes or a non-targeting CRISPRi system grown on TSA supplemented with varying concentrations of IPTG. Approximately 50 – 200 CFUs were spotted on these plates and grown for 24 or 18 hours at 37 °C. Genes of interest and other genes in their operon, if present, are shown in plum and pink, respectively. **(B)** Summary of changes in the oxacillin MIC of *S. aureus* RN4220 carrying an IPTG-inducible CRISPRi system targeting fourteen essential genes, as determined by the checkerboard assays shown in panels (C-R). **(C-J)** Checkerboard MIC assays of oxacillin for *S. aureus* RN4220 carrying an IPTG-inducible CRISPRi system targeting seven essential genes or a non-targeting control. For each essential gene, a 5 x 4 checkerboard with varying IPTG concentrations on one axis was used to identify optimal transcriptional repression. Approximately 50 – 200 CFUs were spotted on TSA plates and incubated for 48 hours at 37 °C. Changes in MIC (ΔMIC) are shown in blue. **(L-R)** Same as (C-J), except CRISPRi targeting an additional seven essential genes was tested in a separate experimental batch. **(S)** *ligA* complementation. "*ligA*" denotes *S. aureus* RN4220 carrying a plasmid encoding an IPTG-inducible CRISPRi system targeting *ligA*. "*ligA-c*" denotes the same strain complemented with a second plasmid encoding an IPTG-inducible *ligA* allele containing synonymous mutations that negate crRNA-target base-pairing. Left: 0.3 mM IPTG and 0 μg/mL oxacillin. Right: 0.3 mM IPTG and 0.05 μg/mL oxacillin. Cells were grown on TSA plates for 24 hours at 37 °C and three serial dilutions are shown. **(T)** Same as (S), except ciprofloxacin was used. **(U)** Same as (R), except complementation was performed for *ruvB*. **(V)** Same as (U), ciprofloxacin was used. **(W)** Same as (C-J), except checkerboard assays measured MICs of ciprofloxacin for *S. aureus* RN4220 carrying an IPTG-inducible CRISPRi system targeting *coaA*, *ribF*, *ppnK*, or a non-targeting control. **(X)** Same as (C), except checkerboard assays measured MICs of erythromycin for *S. aureus* RN4220 carrying an IPTG-inducible CRISPRi system targeting *ruvB* or a non-targeting control. **(Y)** Checkerboard assays measuring the MICs of erythromycin, linezolid, and mupirocin for *S. aureus* RN4220 carrying CRISPRi targeting *rnr* or *rnjA*. Approximately 50 – 200 CFUs were spotted on TSA plates and grown for 48 hours at 37 °C.

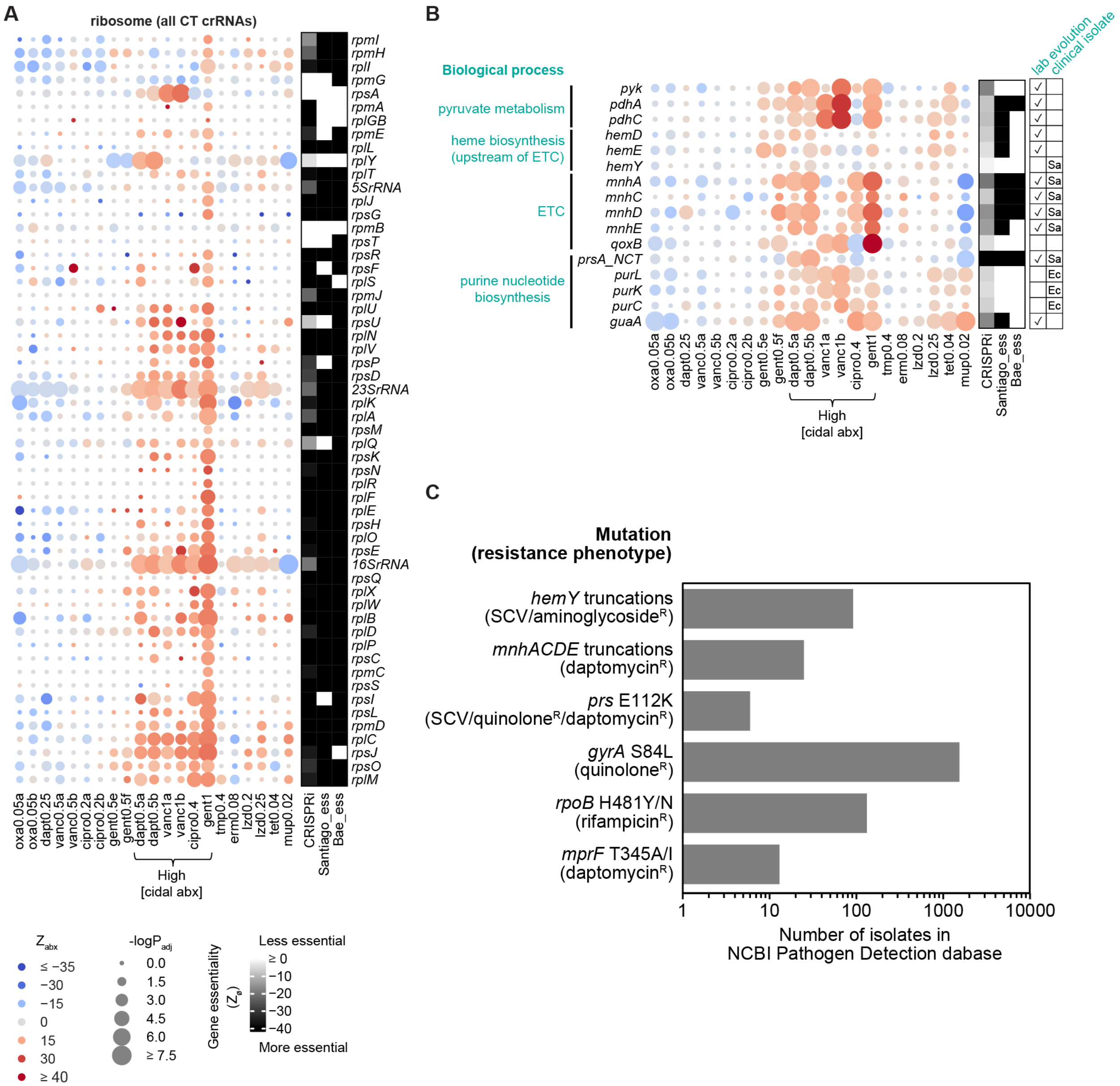

Fig. S6. Other desensitizing antibiotic-gene interactions. (A) Heatmap showing the relative fitness of genes in ribosome (GO:0005840) in ten antibiotics as quantified by all CT crRNAs in CALM-CRISPRi screens (Z_{abx}) in *S. aureus* RN4220. This quantification is incomplete (see "Quantification using NCT crRNAs" in Methods). Antibiotic conditions with replicates are labeled with letters. The only exception is "gent1", which shows Z_{abx} calculated from library treated with 1 µg/mL gent for 4.5 hours from a previous study (*15*). Gene names are annotated by KEGG orthology (ko). Gene knockdowns that decreased and increased relative fitness in antibiotics are shown in blue and red circles, respectively. Size of circle indicates the negative of log-transformed adjusted P-value, calculated by a Mann-Whitney U test between gene-specific crRNAs and null crRNAs (Methods). Gene essentiality quantified by CRISPRi (mean of $Z_{Ø}$ from triplicates) and qualified (binary) by Santiago's (*22*) and Bae's (*24*) Tn-seq studies are in black-white heatmap. **(B)** Same as (A) but showing the relative fitness of selected genes in pyruvate metabolism, heme biosynthesis, ETC, and purine nucleotide biosynthesis. The presence of mutations identified in prior laboratory evolution experiments or clinical isolates is also

indicated. Abbreviations: Sa, *S. aureus*; Ec, *E. coli*. **(C)** Numbers of *S. aureus* isolates (among 4,284 genomes from the NCBI Pathogen Detection database) harboring function-impairing mutations in *hemY*, *mnhACDE*, and *prs* that confer antibiotic resistance. The numbers of isolates harboring canonical antibiotic-resistance-associated mutations affecting *gyrA*, *rpoB*, and *mprF* are also shown. Accession numbers of genomes with identified mutations are provided in Table S18.

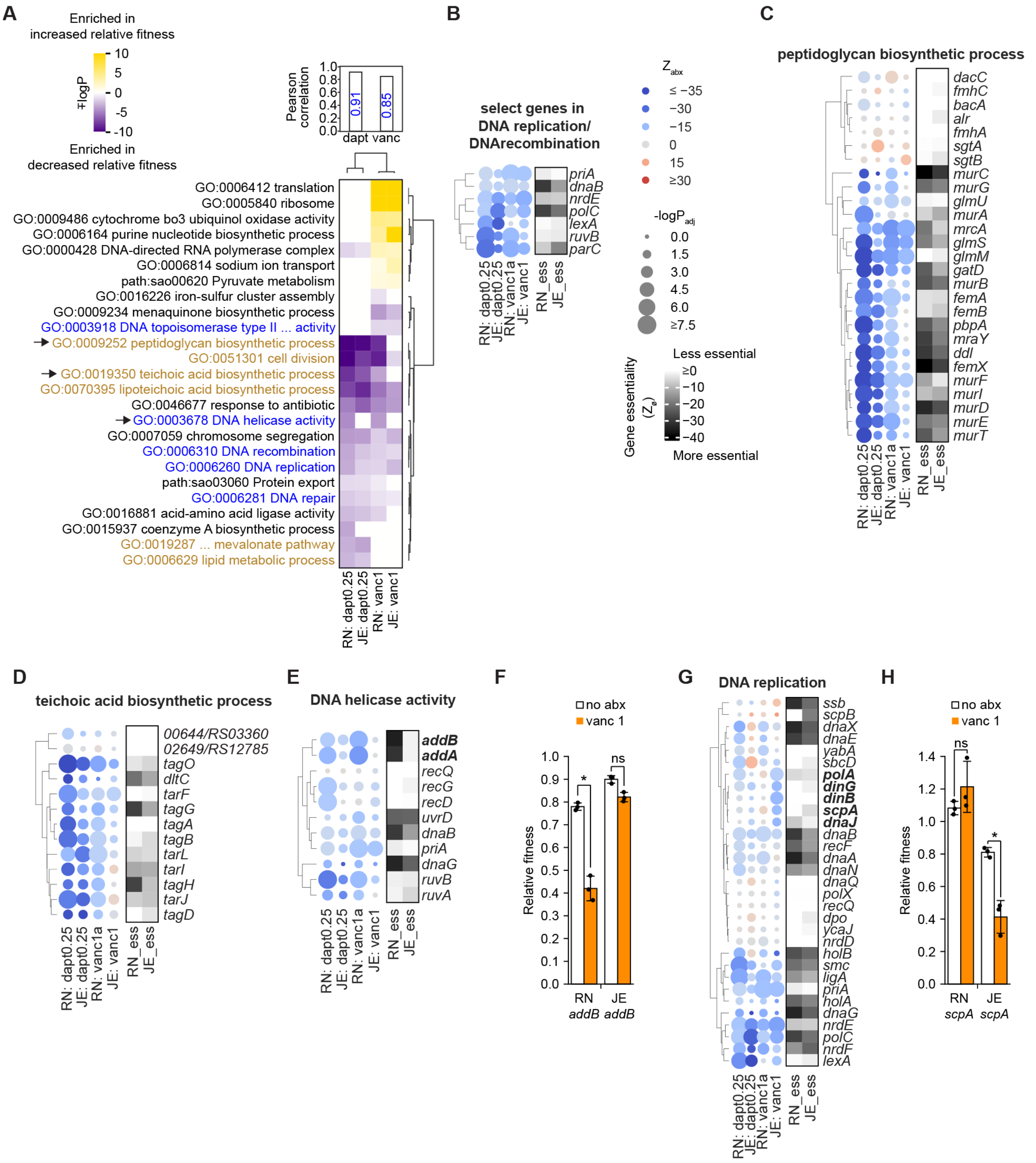

Fig. S7. Antibiotic-gene interactions in MRSA JE2. **(A)** Bottom: Functional enrichment analysis, followed by hierarchical clustering of significantly enriched non-redundant Gene Ontology (GO) terms and KEGG pathways in *S. aureus* RN4220 and JE2. The ∓log(P-value) of GO terms and KEGG pathways significantly enriched ($P < 0.001$) for genes whose repression increased and decreased relative fitness in antibiotics ($|Z_{abx}| \geq 9$ and $P_{adj} < 0.05$) are shown in yellow and purple, respectively. Top: Pearson correlation coefficient for each antibiotic condition between RN4220 and JE2. Clustering of GO terms and KEGG pathways is shown Table S19. **(B)** Heatmap showing the relative fitness of select genes (Z_{abx}) in the DNA

replication and DNA recombination processes under daptomycin and vancomycin in both *S. aureus* RN4220 and JE2. Gene names are annotated by KEGG orthology (ko). Gene knockdowns that decreased and increased relative fitness in antibiotics are shown in blue and red circles, respectively. Size of circle indicates the negative of log-transformed adjusted P-value, calculated by a Mann-Whitney U test between gene-specific crRNAs and null crRNAs (Methods). Gene essentiality quantified by CRISPRi (as $Z_{\emptyset}$ in this study) is also shown in black-white heatmap. $Z_{\emptyset}$ and Z_{abx} of all JE2 genes are shown in Tables S9 and S10, respectively. **(C-E)** Same as (B) but showing genes in other biological processes. **(F)** Pairwise competition assays (Methods) measuring the relative fitness of *S. aureus* RN4220 or JE2 carrying an IPTG-inducible CRISPRi system targeting *addB* under 1 μg/mL vancomycin or no-antibiotic condition. Error bars indicate the standard deviation from three biological replicates. For each strain, a paired t-test was performed between the antibiotic condition and the no-antibiotic condition. * indicates $P < 0.05$. **(G)** Same as (B) but showing genes in DNA replication. **(H)** Same as (F) but showing the relative fitness of repression of *scpA*.

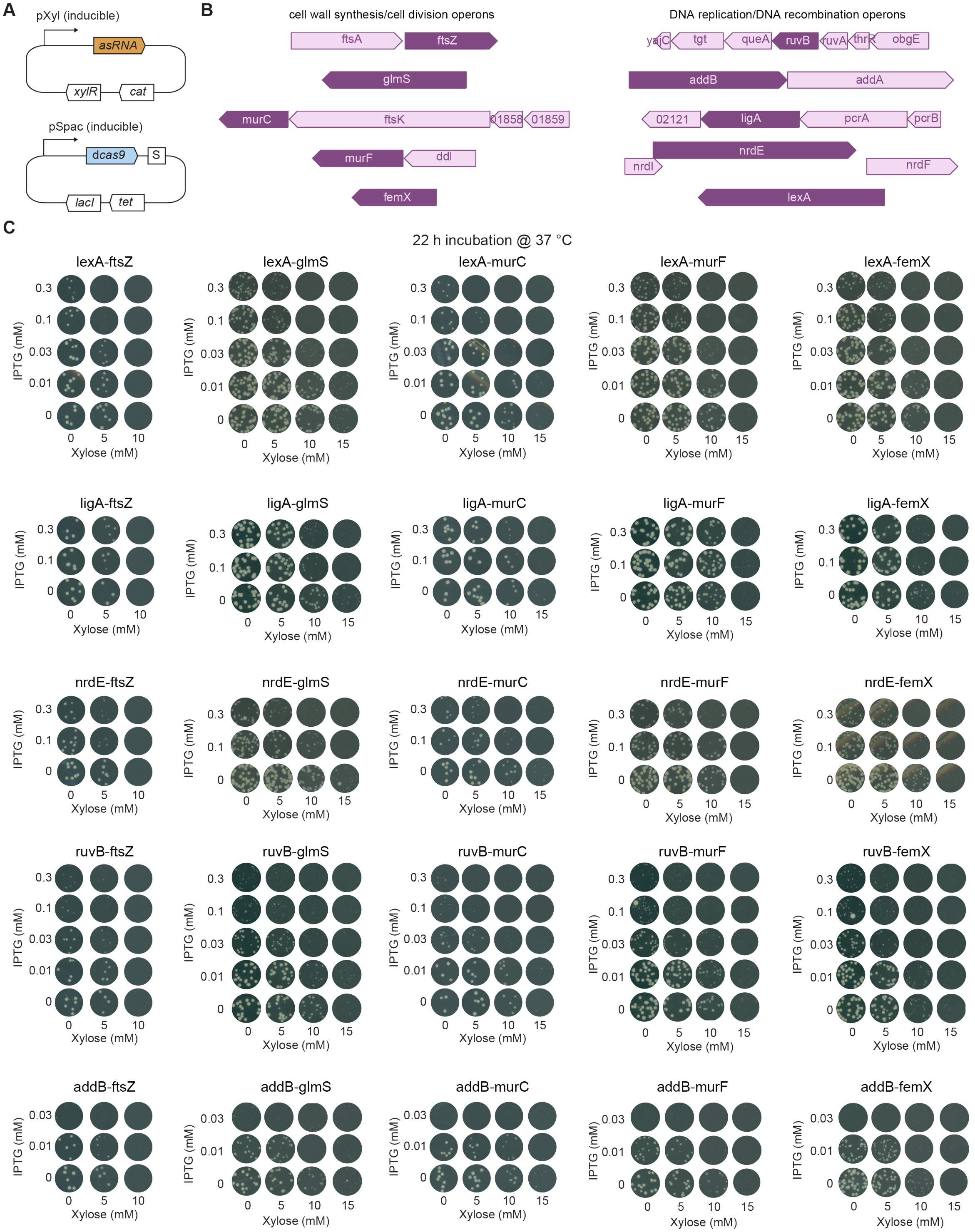
A
pXyl (inducible)
asRNA
xylR
cat
pSpac (inducible)
dcas9
S
lacI
tet
B
cell wall synthesis/cell division operons
ftsA
ftsZ
glmS
murC
ftsK
01858
01859
murF
ddl
femX
DNA replication/DNA recombination operons
tgt
queA
ruvB
obgE
addB
addA
02121
ligA
pcrA
pcrB
nrdE
nrdI
nrdF
lexA
C
22 h incubation @ 37 °C
lexA-ftsZ
lexA-glmS
lexA-murC
lexA-murF
lexA-femX
ligA-ftsZ
ligA-glmS
ligA-murC
ligA-murF
ligA-femX
nrdE-ftsZ
nrdE-glmS
nrdE-murC
nrdE-murF
nrdE-femX
ruvB-ftsZ
ruvB-glmS
ruvB-murC
ruvB-murF
ruvB-femX
addB-ftsZ
addB-glmS
addB-murC
addB-murF
addB-femX
IPTG (mM)
Xylose (mM)

D

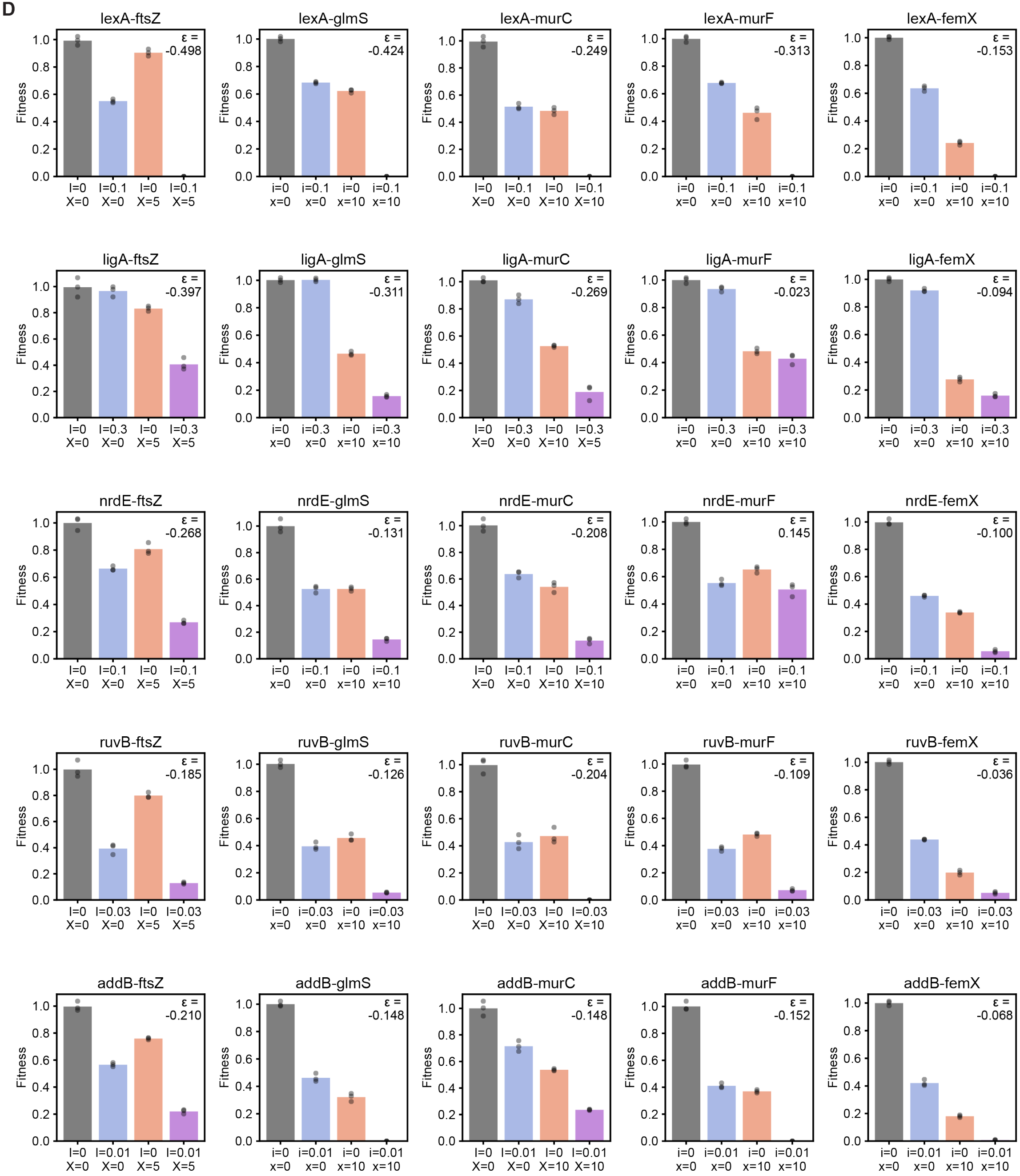

lexA-ftsZ
ε = -0.498
I=0 X=0
I=0.1 X=0
I=0 X=5
I=0.1 X=5
lexA-glmS
ε = -0.424
i=0 x=0
i=0.1 x=0
i=0 x=10
i=0.1 x=10
lexA-murC
ε = -0.249
I=0 X=0
I=0.1 X=0
I=0 X=10
I=0.1 X=10
lexA-murF
ε = -0.313
i=0 x=0
i=0.1 x=0
i=0 x=10
i=0.1 x=10
lexA-femX
ε = -0.153
i=0 x=0
i=0.1 x=0
i=0 x=10
i=0.1 x=10
ligA-ftsZ
ε = -0.397
I=0 X=0
I=0.3 X=0
I=0 X=5
I=0.3 X=5
ligA-glmS
ε = -0.311
i=0 x=0
i=0.3 x=0
i=0 x=10
i=0.3 x=10
ligA-murC
ε = -0.269
I=0 X=0
I=0.3 X=0
I=0 X=10
I=0.3 X=5
ligA-murF
ε = -0.023
i=0 x=0
i=0.3 x=0
i=0 x=10
i=0.3 x=10
ligA-femX
ε = -0.094
i=0 x=0
i=0.3 x=0
i=0 x=10
i=0.3 x=10
nrdE-ftsZ
ε = -0.268
I=0 X=0
I=0.1 X=0
I=0 X=5
I=0.1 X=5
nrdE-glmS
ε = -0.131
i=0 x=0
i=0.1 x=0
i=0 x=10
i=0.1 x=10
nrdE-murC
ε = -0.208
I=0 X=0
I=0.1 X=0
I=0 X=10
I=0.1 X=10
nrdE-murF
ε = 0.145
i=0 x=0
i=0.1 x=0
i=0 x=10
i=0.1 x=10
nrdE-femX
ε = -0.100
i=0 x=0
i=0.1 x=0
i=0 x=10
i=0.1 x=10
ruvB-ftsZ
ε = -0.185
I=0 X=0
I=0.03 X=0
I=0 X=5
I=0.03 X=5
ruvB-glmS
ε = -0.126
i=0 x=0
i=0.03 x=0
i=0 x=10
i=0.03 x=10
ruvB-murC
ε = -0.204
I=0 X=0
I=0.03 X=0
I=0 X=10
I=0.03 X=10
ruvB-murF
ε = -0.109
i=0 x=0
i=0.03 x=0
i=0 x=10
i=0.03 x=10
ruvB-femX
ε = -0.036
i=0 x=0
i=0.03 x=0
i=0 x=10
i=0.03 x=10
addB-ftsZ
ε = -0.210
I=0 X=0
I=0.01 X=0
I=0 X=5
I=0.01 X=5
addB-glmS
ε = -0.148
i=0 x=0
i=0.01 x=0
i=0 x=10
i=0.01 x=10
addB-murC
ε = -0.148
I=0 X=0
I=0.01 X=0
I=0 X=10
I=0.01 X=10
addB-murF
ε = -0.152
i=0 x=0
i=0.01 x=0
i=0 x=10
i=0.01 x=10
addB-femX
ε = -0.068
i=0 x=0
i=0.01 x=0
i=0 x=10
i=0.01 x=10
Fitness
0.0
0.2
0.4
0.6
0.8
1.0

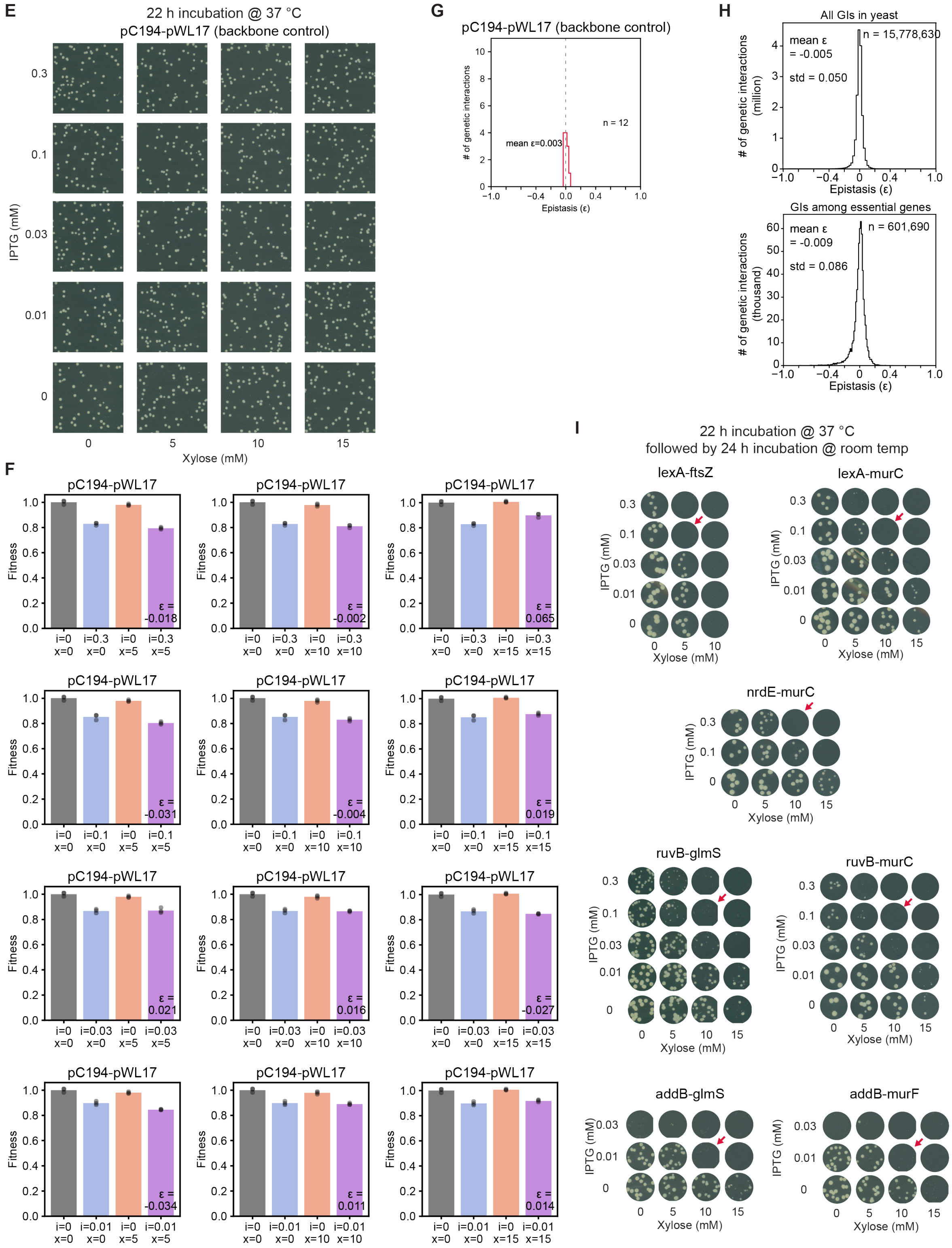
E
22 h incubation @ 37 °C
pC194-pWL17 (backbone control)
IPTG (mM)
0.3
0.1
0.03
0.01
0
0
5
10
15
Xylose (mM)
F
pC194-pWL17
Fitness
ε = -0.018
ε = -0.002
ε = 0.065
ε = -0.031
ε = -0.004
ε = 0.019
ε = 0.021
ε = 0.016
ε = -0.027
ε = -0.034
ε = 0.011
ε = 0.014
i=0 x=0
i=0.3 x=0
i=0 x=5
i=0.3 x=5
i=0 x=10
i=0.3 x=10
i=0 x=15
i=0.3 x=15
i=0.1 x=0
i=0.1 x=5
i=0.1 x=10
i=0.1 x=15
i=0.03 x=0
i=0.03 x=5
i=0.03 x=10
i=0.03 x=15
i=0.01 x=0
i=0.01 x=5
i=0.01 x=10
i=0.01 x=15
G
pC194-pWL17 (backbone control)
# of genetic interactions
mean ε=0.003
n = 12
Epistasis (ε)
H
All GIs in yeast
# of genetic interactions (million)
mean ε = -0.005
std = 0.050
n = 15,778,630
Epistasis (ε)
GIs among essential genes
# of genetic interactions (thousand)
mean ε = -0.009
std = 0.086
n = 601,690
Epistasis (ε)
I
22 h incubation @ 37 °C
followed by 24 h incubation @ room temp
lexA-ftsZ
lexA-murC
nrdE-murC
ruvB-glmS
ruvB-murC
addB-glmS
addB-murF
IPTG (mM)
Xylose (mM)

J

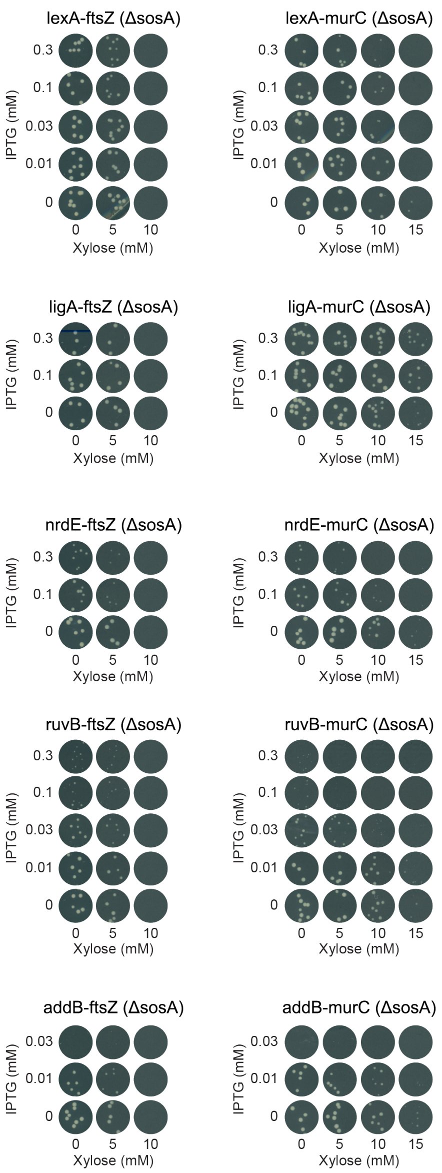
lexA-ftsZ (ΔsosA)
lexA-murC (ΔsosA)
ligA-ftsZ (ΔsosA)
ligA-murC (ΔsosA)
nrdE-ftsZ (ΔsosA)
nrdE-murC (ΔsosA)
ruvB-ftsZ (ΔsosA)
ruvB-murC (ΔsosA)
addB-ftsZ (ΔsosA)
addB-murC (ΔsosA)
IPTG (mM)
Xylose (mM)
0.3
0.1
0.03
0.01
0
0
5
10
15

K

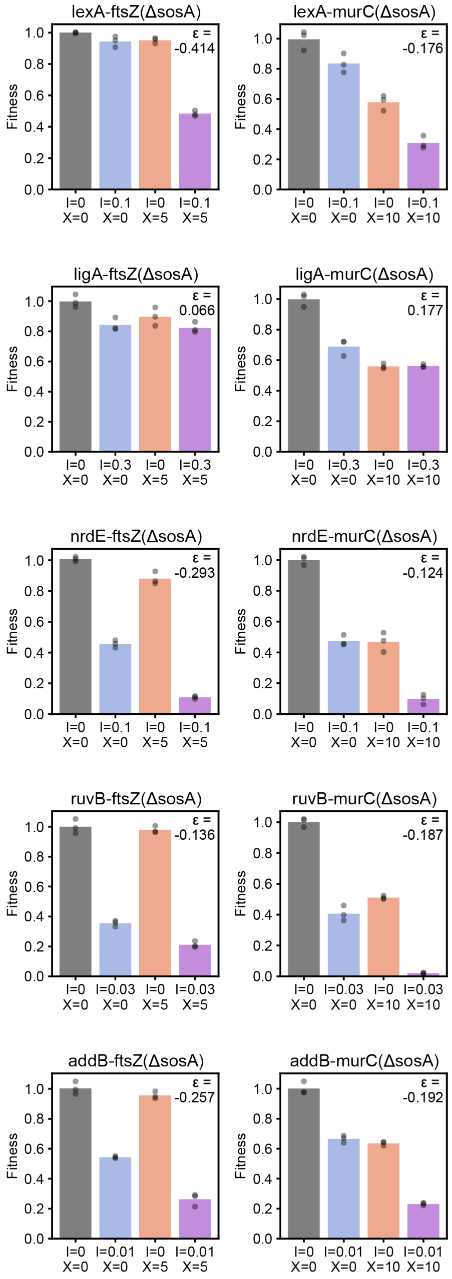
lexA-ftsZ(ΔsosA)
ε = -0.414
lexA-murC(ΔsosA)
ε = -0.176
ligA-ftsZ(ΔsosA)
ε = 0.066
ligA-murC(ΔsosA)
ε = 0.177
nrdE-ftsZ(ΔsosA)
ε = -0.293
nrdE-murC(ΔsosA)
ε = -0.124
ruvB-ftsZ(ΔsosA)
ε = -0.136
ruvB-murC(ΔsosA)
ε = -0.187
addB-ftsZ(ΔsosA)
ε = -0.257
addB-murC(ΔsosA)
ε = -0.192
Fitness
0.0
0.2
0.4
0.6
0.8
1.0
I=0 X=0
I=0.1 X=0
I=0 X=5
I=0.1 X=5
I=0 X=10
I=0.1 X=10
I=0.3 X=0
I=0.3 X=5
I=0.3 X=10
I=0.03 X=0
I=0.03 X=5
I=0.03 X=10
I=0.01 X=0
I=0.01 X=5
I=0.01 X=10

L

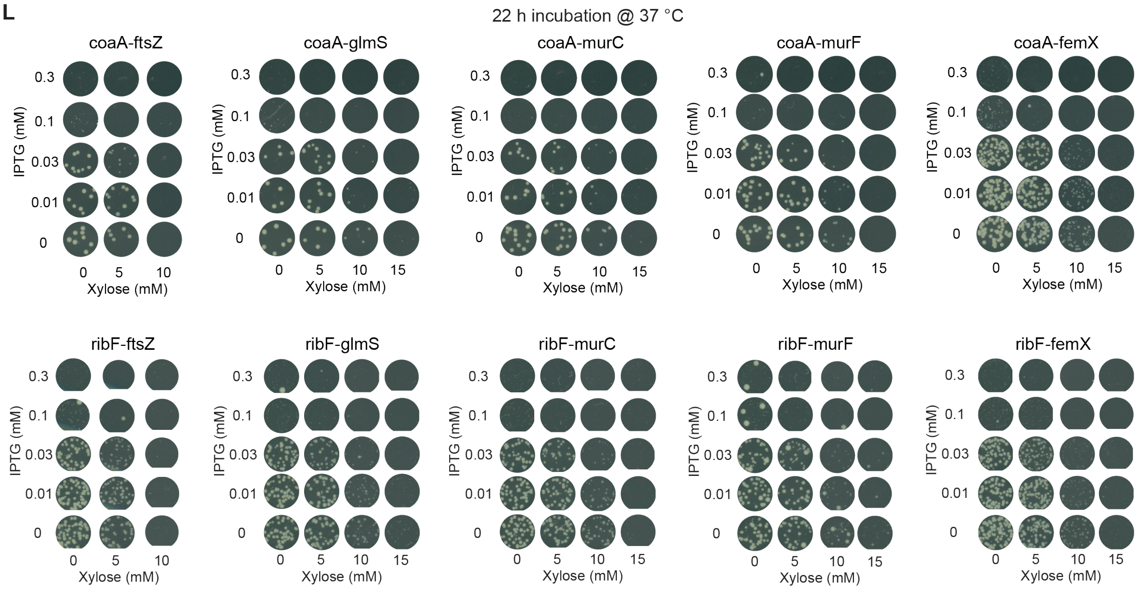
22 h incubation @ 37 °C
coaA-ftsZ
coaA-glmS
coaA-murC
coaA-murF
coaA-femX
ribF-ftsZ
ribF-glmS
ribF-murC
ribF-murF
ribF-femX
IPTG (mM)
0.3
0.1
0.03
0.01
0
Xylose (mM)
0
5
10
15

M

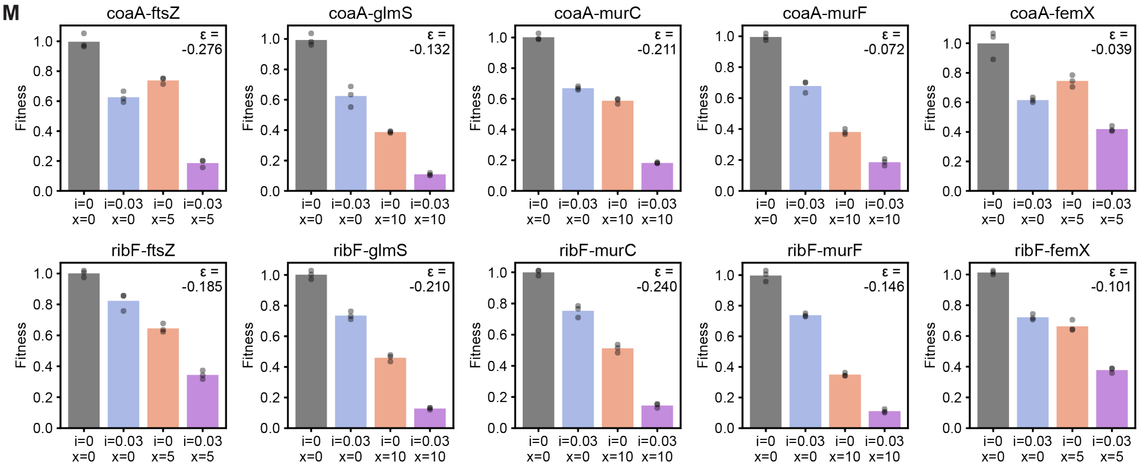
coaA-ftsZ
ε = -0.276
coaA-glmS
ε = -0.132
coaA-murC
ε = -0.211
coaA-murF
ε = -0.072
coaA-femX
ε = -0.039
ribF-ftsZ
ε = -0.185
ribF-glmS
ε = -0.210
ribF-murC
ε = -0.240
ribF-murF
ε = -0.146
ribF-femX
ε = -0.101
Fitness
0.0
0.2
0.4
0.6
0.8
1.0
i=0 x=0
i=0.03 x=0
i=0 x=5
i=0.03 x=5
i=0 x=10
i=0.03 x=10

Fig. S8. Quantification of genetic interactions. (**A**) The two-plasmid system used to quantify gene-gene interactions. pSpac and pXyl are IPTG- and xylose-inducible promoters driving the expression of dCas9/spacer (denoted as "S") and antisense RNA (asRNA), respectively. Spacer is the DNA precursor to crRNA. (**B**) Operons of five cell wall synthesis/cell division and five DNA replication/DNA recombination genes. Targeted genes and other genes in their operons (if present) are shown in plum and pink, respectively. (**C**) Checkerboard assays to quantify genetic interactions between five cell wall synthesis/cell division genes and five DNA replication/DNA recombination genes. In each panel, RN4220 cells carrying IPTG-inducible dCas9 targeting a DNA replication/DNA recombination gene and xylose-inducible asRNA targeting a cell wall synthesis/cell division gene were plated on TSA plates containing various combinations of IPTG and xylose and grown for 22 hours at 37 °C. The order of genetic pairs is the same as those shown in Fig. 7A. (**D**) Quantification of fitness and epistasis for checkerboard assays in (C). Fitness was quantified by selecting appropriate inducer concentrations and measuring colony sizes (Methods). Epistasis between gene A and gene B ($\varepsilon_{A,B}$) was calculated as $\varepsilon_{A,B} = W_{A,B} - W_A \bullet W_B$, where W stands for fitness. ε is shown as the mean of three biological replicates. (**E**) Checkerboard assay to quantify baseline epistasis between plasmid backbones pC194 and pWL17. RN4220 cells carrying both plasmid backbones, pC194 and pWL17, were plated on TSA containing various combinations of IPTG and xylose and grown for 22 hours at 37 °C. (**F**) Quantification of fitness and epistasis for checkerboard assays in (E). (**G**) Distribution of baseline epistasis (ε) among 12 IPTG-xylose combinations in (F). (**H**) To contextualize the epistasis measured in our study, we re-plotted the distribution of epistasis among ~15 million gene pairs (top) and 600,000 essential gene pairs (bottom) measured in a comprehensive genetic interaction network study in yeast (*5*). (**I**) Select checkerboard assays from (C) that were incubated for an additional 24 hours at room temperature. Arrows indicate combinations of IPTG and xylose that yielded few or no viable colonies. (**J**) Checkerboard assays to quantify genetic interactions between two cell wall synthesis/cell division genes and five DNA replication/DNA recombination genes in Δ*sosA* background. (**K**) Quantification of fitness and epistasis for checkerboard assays in (J). (**L**) Checkerboard assays to quantify genetic interactions between five cell wall synthesis/cell division genes and *coaA* and *ribF*. (**M**) Quantification of fitness and epistasis for checkerboard assays in (L).

A

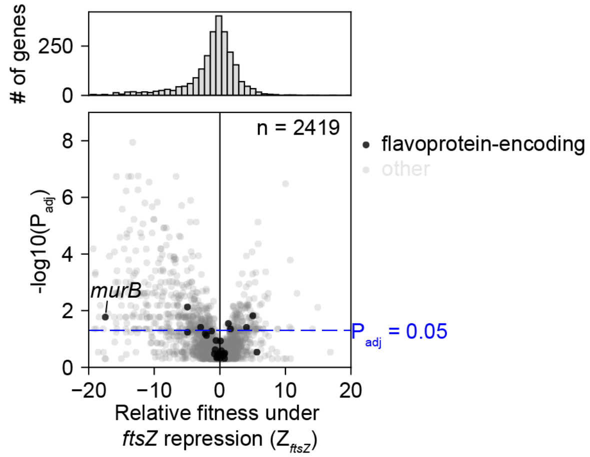

B

flavoprotein-encoding genes

Locus tag	Z_{ftsZ}	-log(P_{adj})
SAOUHSC_00752(*murB*)	-17.471	1.771
SAOUHSC_01043	-4.938	2.129
SAOUHSC_01499	-4.921	1.238
SAOUHSC_00878	-2.935	1.412
SAOUHSC_00364	-2.226	1.186
SAOUHSC_01884	-2.034	1.123
SAOUHSC_02654	-1.260	1.277
SAOUHSC_02579	-0.831	0.472
SAOUHSC_01278	-0.708	0.618
SAOUHSC_02927	-0.582	0.944
SAOUHSC_02932	-0.476	0.330
SAOUHSC_02684	-0.384	0.418
SAOUHSC_03052	-0.351	0.363
SAOUHSC_00875	-0.309	0.433
SAOUHSC_00908	-0.062	0.491
SAOUHSC_02881	0.068	0.324
SAOUHSC_01614	0.092	0.930
SAOUHSC_00699	0.127	0.428
SAOUHSC_00197	0.206	0.588
SAOUHSC_02647	0.443	0.407
SAOUHSC_00339	0.671	0.458
SAOUHSC_00581	0.710	0.310
SAOUHSC_01104	0.749	0.500
SAOUHSC_00037	1.289	1.543
SAOUHSC_02947	1.629	1.352
SAOUHSC_01960	4.066	1.411
SAOUHSC_01223	5.041	1.819
SAOUHSC_00785	5.662	0.533

Fig. S9. Relative fitness of flavoprotein-encoding genes under mild *ftsZ* repression in *S. aureus* RN4220. (A) Volcano plot showing the relative fitness of genes under mild *ftsZ* repression to plain media, Z_{ftsZ}. Flavoprotein-encoding genes are highlighted in black. **(B)** Table of Z_{ftsZ} and -log(P_{adj}) for all flavoprotein-encoding genes. List of flavoprotein-encoding genes was obtained from https://www.uniprot.org/uniprotkb?dir=ascend&query=%28organism_id%3A93061%29+AND+%28cc_cofactor_chebi%3A%22CHEBI%3A16238%22%29&sort=gene

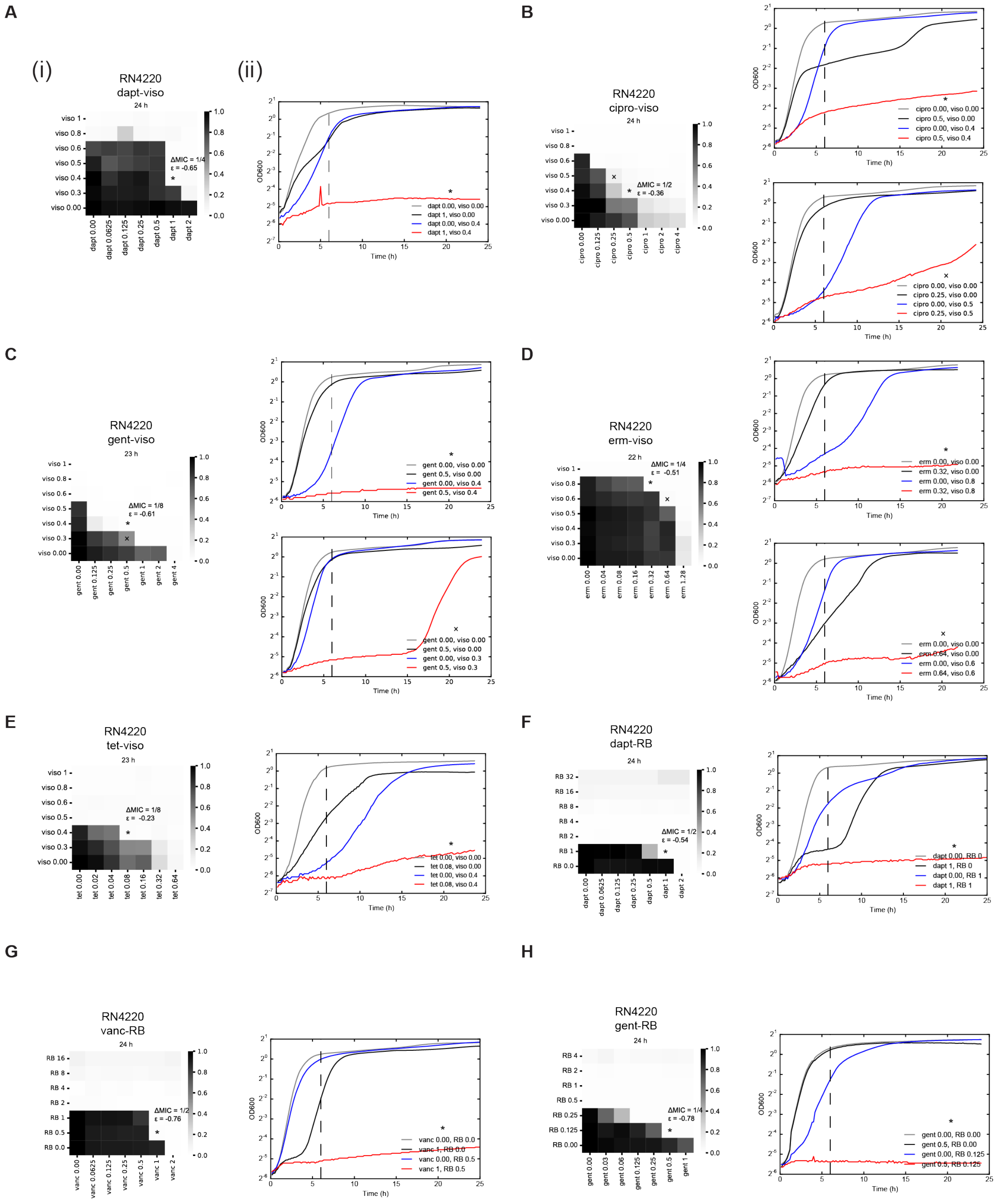

A
(i)
RN4220
dapt-viso
24 h
ΔMIC = 1/4
ε = -0.65
(ii)
OD600
Time (h)
dapt 0.00, viso 0.00
dapt 1, viso 0.00
dapt 0.00, viso 0.4
dapt 1, viso 0.4
B
RN4220
cipro-viso
24 h
ΔMIC = 1/2
ε = -0.36
cipro 0.00, viso 0.00
cipro 0.5, viso 0.00
cipro 0.00, viso 0.4
cipro 0.5, viso 0.4
cipro 0.00, viso 0.00
cipro 0.25, viso 0.00
cipro 0.00, viso 0.5
cipro 0.25, viso 0.5
C
RN4220
gent-viso
23 h
ΔMIC = 1/8
ε = -0.61
gent 0.00, viso 0.00
gent 0.5, viso 0.00
gent 0.00, viso 0.4
gent 0.5, viso 0.4
gent 0.00, viso 0.00
gent 0.5, viso 0.00
gent 0.00, viso 0.3
gent 0.5, viso 0.3
D
RN4220
erm-viso
22 h
ΔMIC = 1/4
ε = -0.51
erm 0.00, viso 0.00
erm 0.32, viso 0.00
erm 0.00, viso 0.8
erm 0.32, viso 0.8
erm 0.00, viso 0.00
erm 0.64, viso 0.00
erm 0.00, viso 0.6
erm 0.64, viso 0.6
E
RN4220
tet-viso
23 h
ΔMIC = 1/8
ε = -0.23
tet 0.00, viso 0.00
tet 0.08, viso 0.00
tet 0.00, viso 0.4
tet 0.08, viso 0.4
F
RN4220
dapt-RB
24 h
ΔMIC = 1/2
ε = -0.54
dapt 0.00, RB 0
dapt 1, RB 0
dapt 0.00, RB 1
dapt 1, RB 1
G
RN4220
vanc-RB
24 h
ΔMIC = 1/2
ε = -0.76
vanc 0.00, RB 0.0
vanc 1, RB 0.0
vanc 0.00, RB 0.5
vanc 1, RB 0.5
H
RN4220
gent-RB
24 h
ΔMIC = 1/4
ε = -0.78
gent 0.00, RB 0.00
gent 0.5, RB 0.00
gent 0.00, RB 0.125
gent 0.5, RB 0.125

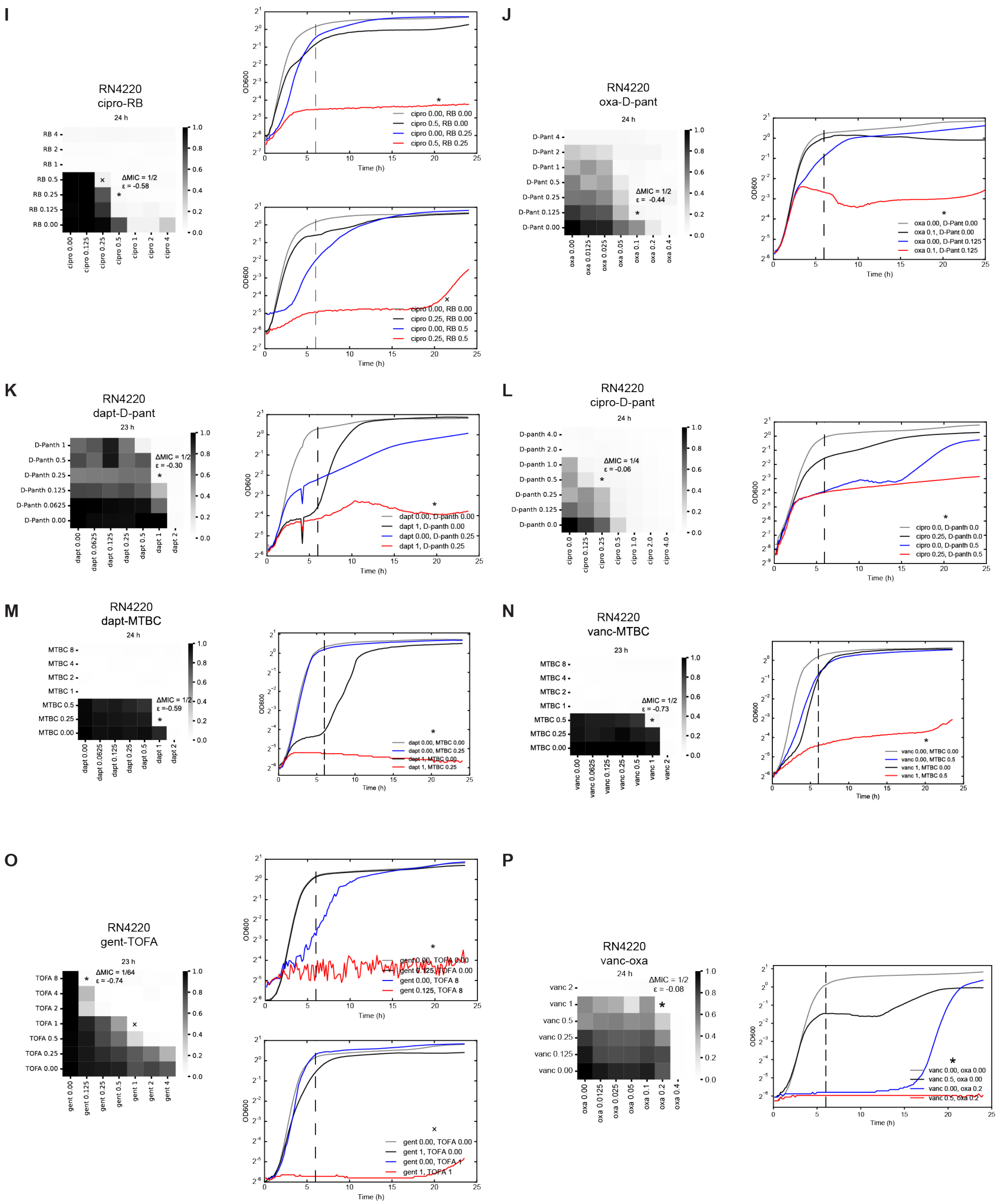
I
RN4220
cipro-RB
24 h
ΔMIC = 1/2
ε = -0.58
cipro 0.00, RB 0.00
cipro 0.5, RB 0.00
cipro 0.00, RB 0.25
cipro 0.5, RB 0.25
cipro 0.00, RB 0.00
cipro 0.25, RB 0.00
cipro 0.00, RB 0.5
cipro 0.25, RB 0.5
Time (h)
OD600
J
RN4220
oxa-D-pant
24 h
ΔMIC = 1/2
ε = -0.44
oxa 0.00, D-Pant 0.00
oxa 0.1, D-Pant 0.00
oxa 0.00, D-Pant 0.125
oxa 0.1, D-Pant 0.125
K
RN4220
dapt-D-pant
23 h
ΔMIC = 1/2
ε = -0.30
dapt 0.00, D-panth 0.00
dapt 1, D-panth 0.00
dapt 0.00, D-panth 0.25
dapt 1, D-panth 0.25
L
RN4220
cipro-D-pant
24 h
ΔMIC = 1/4
ε = -0.06
cipro 0.0, D-panth 0.0
cipro 0.25, D-panth 0.0
cipro 0.0, D-panth 0.5
cipro 0.25, D-panth 0.5
M
RN4220
dapt-MTBC
24 h
ΔMIC = 1/2
ε = -0.59
N
RN4220
vanc-MTBC
23 h
ΔMIC = 1/2
ε = -0.73
O
RN4220
gent-TOFA
23 h
ΔMIC = 1/64
ε = -0.74
gent 0.00, TOFA 0.00
gent 1, TOFA 0.00
gent 0.00, TOFA 1
gent 1, TOFA 1
P
RN4220
vanc-oxa
24 h
ΔMIC = 1/2
ε = -0.08
vanc 0.00, oxa 0.00
vanc 0.5, oxa 0.00
vanc 0.00, oxa 0.2
vanc 0.5, oxa 0.2

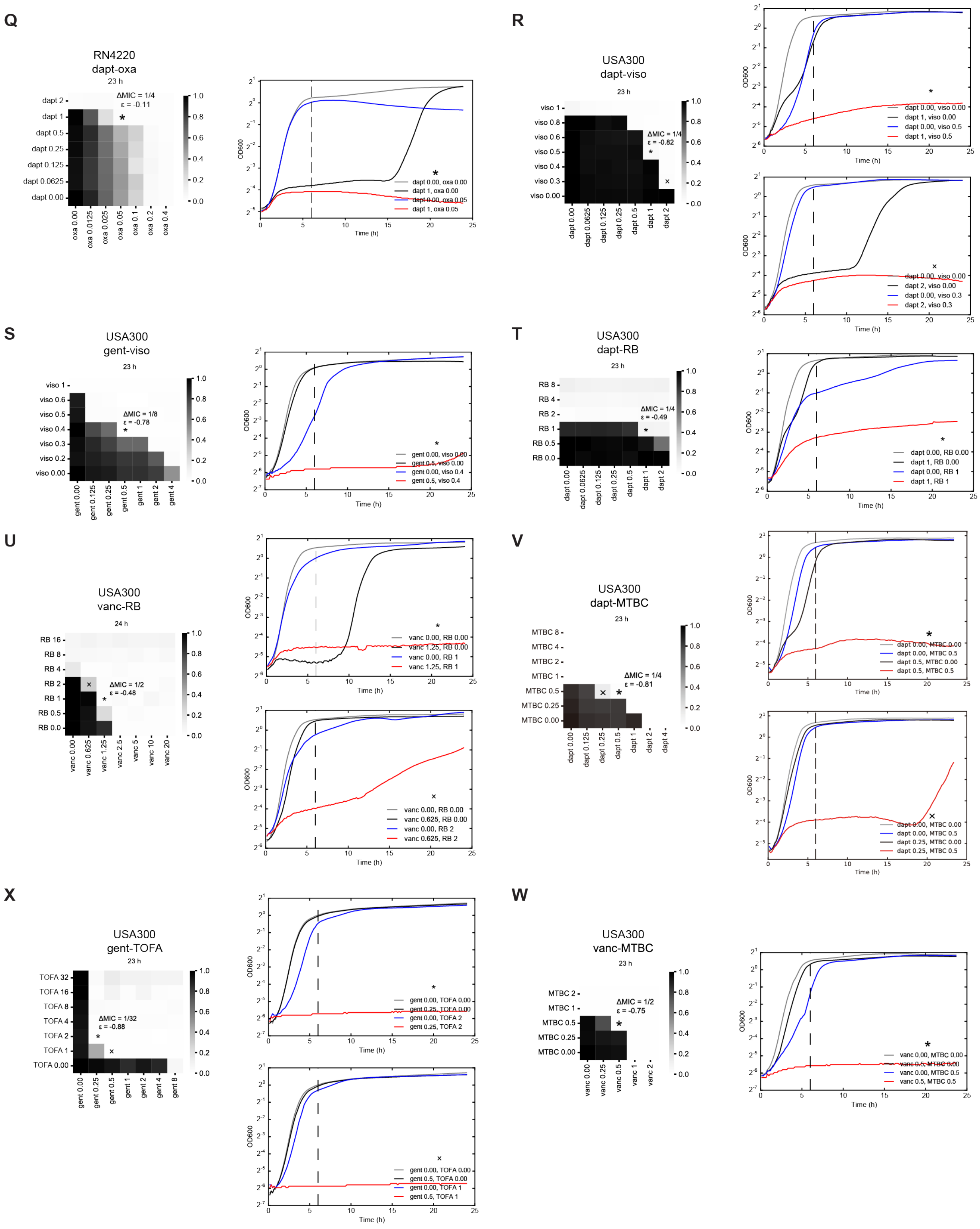

Q
RN4220
dapt-oxa
23 h
ΔMIC = 1/4
ε = -0.11
dapt 0.00, oxa 0.00
dapt 1, oxa 0.00
dapt 0.00, oxa 0.05
dapt 1, oxa 0.05
R
USA300
dapt-viso
23 h
ΔMIC = 1/4
ε = -0.82
dapt 0.00, viso 0.00
dapt 1, viso 0.00
dapt 0.00, viso 0.5
dapt 1, viso 0.5
dapt 0.00, viso 0.00
dapt 2, viso 0.00
dapt 0.00, viso 0.3
dapt 2, viso 0.3
S
USA300
gent-viso
23 h
ΔMIC = 1/8
ε = -0.78
gent 0.00, viso 0.00
gent 0.5, viso 0.00
gent 0.00, viso 0.4
gent 0.5, viso 0.4
T
USA300
dapt-RB
23 h
ΔMIC = 1/4
ε = -0.49
dapt 0.00, RB 0.00
dapt 1, RB 0.00
dapt 0.00, RB 1
dapt 1, RB 1
U
USA300
vanc-RB
24 h
ΔMIC = 1/2
ε = -0.48
vanc 0.00, RB 0.00
vanc 1.25, RB 0.00
vanc 0.00, RB 1
vanc 1.25, RB 1
vanc 0.00, RB 0.00
vanc 0.625, RB 0.00
vanc 0.00, RB 2
vanc 0.625, RB 2
V
USA300
dapt-MTBC
23 h
ΔMIC = 1/4
ε = -0.81
dapt 0.00, MTBC 0.00
dapt 0.00, MTBC 0.5
dapt 0.5, MTBC 0.00
dapt 0.5, MTBC 0.5
dapt 0.00, MTBC 0.00
dapt 0.00, MTBC 0.5
dapt 0.25, MTBC 0.00
dapt 0.25, MTBC 0.5
X
USA300
gent-TOFA
23 h
ΔMIC = 1/32
ε = -0.88
gent 0.00, TOFA 0.00
gent 0.25, TOFA 0.00
gent 0.00, TOFA 2
gent 0.25, TOFA 2
gent 0.00, TOFA 0.00
gent 0.5, TOFA 0.00
gent 0.00, TOFA 1
gent 0.5, TOFA 1
W
USA300
vanc-MTBC
23 h
ΔMIC = 1/2
ε = -0.75
vanc 0.00, MTBC 0.00
vanc 0.5, MTBC 0.00
vanc 0.00, MTBC 0.5
vanc 0.5, MTBC 0.5
OD600
Time (h)

Fig. S10. Checkerboard assays for antibiotic-SMI interactions. (**A**) Checkerboard assay of the daptomycin-visomitin combination in *S. aureus* RN4220. Panel (i) shows bacterial growth at various antibiotic and SMI concentrations, normalized to growth in plain media (ie, $\mathrm{OD}_{\mathrm{norm}}^{\mathrm{abx,\ SMI}} = \mathrm{OD}^{\mathrm{abx,\ SMI}} / \mathrm{OD}^{\mathrm{plain}}$) at 22-24 hours post-inoculation. An asterisk (*) marks the well used to calculate the change in MIC (ΔMIC) and epistasis (ε) (Methods). Panel (ii) shows the four growth curves related to the asterisk-marked well: plain media (gray), antibiotic alone (black), SMI alone (blue), and antibiotic-SMI combination (red). The dotted line indicates time = 6 h. In some cases, an additional well with similar growth profile to the asterisk-marked well was marked with "x", and its corresponding growth curves are also shown. Unless otherwise noted, concentrations of antibiotics and SMIs are in μg/mL. D-pantothenol concentrations are in mg/mL. (**B to Q**) Checkerboard assays for additional antibiotic-SMI combinations in *S. aureus* RN4220. (**R to X**) Checkerboard assays for antibiotic-SMI combinations in *S. aureus* USA300-LAC.

List of supplementary tables

Table S1. NCTC8325 operons annotated by BioCyc.

Table S2. RN4220 gene fitness in plain TSB media ($Z_{Ø}$).

Table S3. RN4220 gene relative fitness in antibiotics (Z_{abx}) quantified by CT crRNAs.

Table S4. RN4220 gene relative fitness in antibiotics (Z_{abx}) quantified by NCT crRNAs.

Table S5. Hierarchical clustering of 650 RN4220 genes significantly modulating antibiotic sensitivity.

Table S6. Functional enrichment and hierarchical clustering of significant GOs under antibiotic conditions in RN4220.

Table S7. Functional enrichment (using only the last gene in each operon) and hierarchical clustering of significant GOs under antibiotic conditions in RN4220.

Table S8. Functional enrichment and hierarchical clustering of significant KEGG pathways under antibiotic conditions in RN4220.

Table S9. JE2 gene fitness in plain TSB media ($Z_{Ø}$).

Table S10. JE2 gene relative fitness in antibiotics (Z_{abx}) quantified by CT crRNAs.

Table S11. JE2 gene relative fitness in antibiotics (Z_{abx}) quantified by NCT crRNAs.

Table S12. Pearson correlation matrix for 440 essential RN4220 genes, generated by 14 antibiotic-gene interaction profiles.

Table S13. Gene coordinates of the essential gene similarity network in RN4220.

Table S14. Correlation matrix of 27 biological processes.

Table S15. RN4220 gene relative fitness under mild *ftsZ* repression (Z_{ftsZ}) quantified by CT crRNAs.

Table S16. Epistasis and P-values for genetic interactions in RN4220.

Table S17. -log2(ΔMIC) and epistasis values for drug-drug interactions in RN4220 and USA300.

Table S18. Selected mutations of *S. aureus* genes detected in the NCBI Pathogen Detection database.

Table S19. Functional enrichment and hierarchical clustering of significant GOs under antibiotic conditions in RN4220 and JE2.

Table S20. Key reagents used in this study.

Table S21. Sequences of spacers, asRNAs, and oligos used in this study.
